# Proceedings of the 11th International Association of Veterinary Rehabilitation and Physical Therapy Symposium

**DOI:** 10.1186/s13028-023-00706-w

**Published:** 2023-12-15

**Authors:** 

## O.01 Does transcutaneous electrical nerve stimulation change the physical activity in dogs with pain from the locomotor apparatus?

### **Anja Pedersen**^1^, Anna Bergh^1^, Linn Dadell^1^, Anja Babra^1^

#### ^1^Swedish University of Agricultural Sciences SLU, Uppsala, Sweden

##### **Correspondence:** Anja Pedersen (anja.pedersen@slu.se)

*Acta Veterinaria Scandinavica* 2023, **65(Suppl 1)**:O.01

**Background:** Transcutaneous electrical nerve stimulation (TENS) is often used as a pain-relieving treatment in dogs. Despite its frequent use, the evidence for its clinical efficacy is sparse. However, it has been shown that treatment with TENS, on five dogs with arthrosis, increased weight bearing up to 120 min after treatment [1]. This randomized, controlled cross-over study aimed to investigate the effect of TENS on physical activity in dogs with chronic pain from the locomotor apparatus.

**Materials and methods:** The study included 14 dogs with a low to moderate degree of lameness and age over 1 year. The dogs were diagnosed with pain from the locomotor apparatus by clinical examination before inclusion in the study. The dogs were treated with high-frequency TENS set at a constant current of 80 Hz and 100 µs for 45 min, once daily for eight or ten consecutive days. The electrodes were placed on the skin adjacent to the most painful joint, assessed by clinical examination, and confirmed by journal records.

**Results:** There was a washout period between the randomized interventions active treatment and placebo (Fig. 1). The effect of TENS was evaluated by obtaining activity level data with an activity monitor (ActiGraph GT3X +) before and after treatment. Registrations were conducted for the full intervention (8 to 10 days) and a baseline (2–13 days).

**Figure 1 (abstract O.01) Figa:**
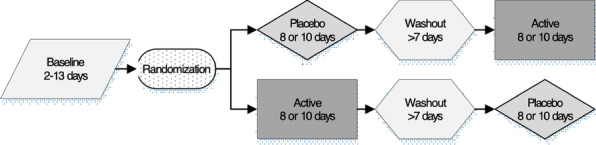
Study design

The activity monitor data was processed in Matlab by a person blinded to the order of treatments. Counts per minute were analyzed and the activity was divided into four categories: sedentary, light, moderate, and vigorous [2]. A one-sided paired t-test was performed in Excel and the significant P value was set to < 0.05. Preliminary results show significant changes were seen in the light activity category (P = 0.02). There were no significant changes before and after treatment for the other categories (sedentary P = 0.26, moderate P = 0.39, and vigorous P = 0.35).

**Conclusion:** The results indicate that TENS may change the time spent in the light activity category. Thus, TENS could have a pain-relieving effect and affect the activity levels of dogs in chronic pain. However, the results need to be confirmed by additional studies.

## References


Levine D, Johnston K, Price M, Schneider N, Millis D. The effect of TENS on osteoarthritic pain in the stifle of dogs. Proceedings of the 2nd International Symposium on Rehabilitation and Physical Therapy in Veterinary Medicine; 2002.Yam PS, Penpraze V, Young D, Todd MS, Cloney AD, Houston-Callaghan KA, et al. Validity, practical utility and reliability of Actigraph accelerometry for the measurement of habitual physical activity in dogs. J Small Anim Pract. 2011;52:86–91.

## O.02 Limb circumference measurements in standing dogs a reliability study

### Zebastian Cederblad^1^, Josefin Söder^1^, Erika Roman^2,3^, Laura Vossen^2^, **Anna Bergh**^1^

#### ^1^Department of Clinical Sciences, Swedish University of Agricultural Sciences, Uppsala, Sweden; ^2^Department of Anatomy, Physiology, and Biochemistry, Swedish University of Agricultural Sciences, Uppsala, Sweden; ^3^Department of Pharmaceutical Biosciences, Uppsala University, Uppsala, Sweden

##### **Correspondence:** Anna Bergh (anna.bergh@slu.se)

*Acta Veterinaria Scandinavica* 2023, **65(Suppl 1)**:O.02

**Background:** Muscle strength is an essential part of physical function and may affect daily physical activity. Evaluating muscle strength is vital to assess a dog´s general physical condition and to evaluate progress in physical training and rehabilitation programs. There are few ways to evaluate muscle strength directly in animals. Limb circumference, as assessed with a tape measure, has been shown to provide reliable measurement of the proximal antebrachium and possibly the thigh [1, 2]. For thigh circumference, the method's validity has been questioned [3]; furthermore, studies have not been conducted on standing dogs. Since the tape measure is a simple and affordable tool to measure the circumference, it is important to investigate its reliability. The present study aimed to determine, in a clinical setting, the inter- and intra-rater reliability of limb circumference measures in standing dogs.

**Material and methods:** Circumferential measurements were conducted on 37 healthy dogs of various breeds, sex, and age. The dogs were standing squarely, and measurements were performed with a tape measure (dynamometer) at four standardized locations: lower brachium, upper antebrachium, lower thigh, and upper crus (Fig. 1). Measurements were conducted the same day, in triplicates by two examiners who were blinded to the measurement results. Inter-rater reliability was calculated as the intra-class correlation (ICC) between observers for the exact measurement. In contrast, intra-rater reliability was the ICC) between subsequent measurements by the same examiner. ICCs with P < 0.05 were considered statistically significant.

**Results:** Results showed overall high inter-rater reliability (ICCs ≥ 0.091, P < 0.01) and very high intra-rater reliability (ICCs ≥ 0.99, P < 0.01). Inter-rater reliability was highest for antebrachium (ICC ≥ 0.99, P < 0.01) and somewhat lower for thigh (ICC ≥ 0.91, P < 0.01).

**Figure 1 (abstract O.02) Figb:**
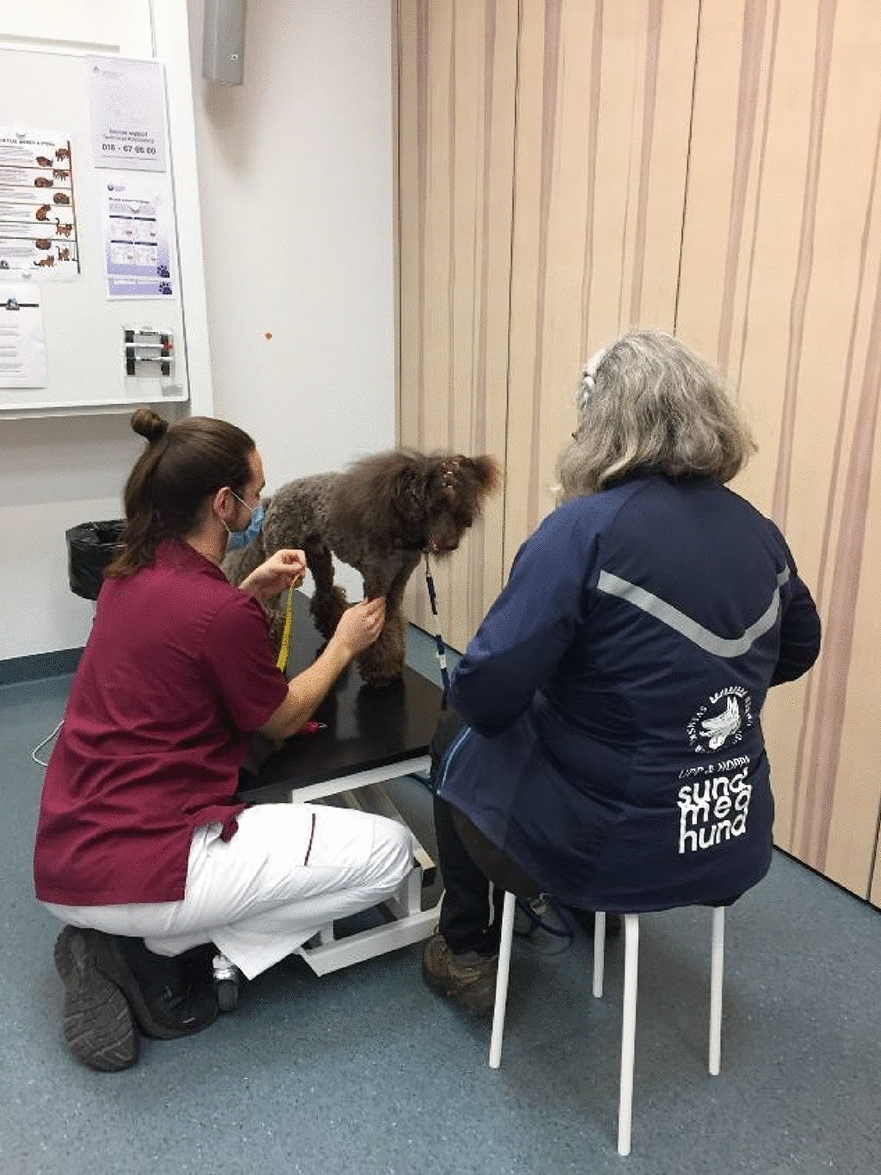
Limb circumference measurement in a standing dog

**Conclusion:** Results indicate that tape measures represent a promising tool for estimating limb circumference in standing dogs. We suggest that it may be used to estimate muscle mass when direct measurements are unavailable. Future studies should investigate the correlation between tape measurements of limb circumference and muscle mass in dogs.

## Acknowledgments

The authors want to express their sincere gratitude to the dog owners for letting their dogs participate in the study.

## References


McCarthy DA, Millis DL, Levine D, Weigel JP. Variables affecting thigh girth measurement and observer reliability in dogs. Front Vet Sci. 2018; 5:203Duerr FM, Bascuñán AL, Kieves N, Goh C, Hart J, Regier P, et al.. Evaluation of factors influencing thigh circumference measurement in dogs. Vet Evidence. 2016; doi:10.18849Smith TJ, Baltzer WI, Jelinski SE, Salinardi BJ. Inter-and intratester reliability of anthropometric assessment of limb circumference in Labrador retrievers. Vet Surg. 42:316–321

## O.03 Effect of kinesiology taping on gait and selected exercises in dogs

### **Darryl Millis**^1^, Rebecca Noel^1^, Krysta Janas^1^, Leann Shaw^1^, Nicholas Millis^1^

#### ^1^Department of Small Animal Clinical Sciences, University of Tennessee, Knoxville, Tennessee, USA

##### **Correspondence:** Darryl Millis (dmillis@utk.edu)

*Acta Veterinaria Scandinavica* 2023, **65(Suppl 1)**:O.03

**Background:** The use of kinesiology taping has increased in human [1] and veterinary medicine. Prospective evaluation of its effect on gait and mobility conditions is essential to evaluate its efficacy. The purpose of this study was to evaluate the effect of kinesiology taping on kinetic characteristics of the pelvic limb and kinematic characteristics of the tarsus while performing selected exercises in dogs.

**Materials and methods:** Ten clinically normal, healthy, adult mixed-breed dogs were recruited for this study. Reflective markers were applied to the skin of the left and right pelvic limbs for each participant. Eight infrared cameras were positioned around a 13 m platform containing a force platform. Dogs were walked, trotted, and led over cavaletti rails with and without kinesiology tape from the distal tibia, crossing the cranial surface of the tarsus, and extending to the dorsal surface of the metatarsals. The trial was repeated 2 h later. Maximum flexion and extension in the sagittal plane were measured for each dog. Peak vertical (Z_Peak_) was determined as a percent of body weight.

**Results:** There were no differences in kinematic or kinetic measurements related to the kinesiology tape application or time period (P > 0.05). Tarsal Joint flexion and tarsal range of motion were increased in trotting dogs and dogs stepping over cavaletti rails as compared with walking (P < 0.05), and ground reaction forces were greater in trotting dogs compared to walking dogs (P < 0.05).

**Conclusion:** Kinesiology tape had no apparent effect on the gait of normal dogs ue conditions of this study. Trotting increased peak vertical force and walking over cavaletti rails and trotting increased tarsal flexion and range of motion.


**Reference**
Drouin JL, McAlpine CT, Primak KA, et al. The effects of kinesiotape on athletic-based performance outcomes in healthy, active individuals: a literature synthesis. J Can Chiropr Assoc 2013;57:356-65.


## O.05 Rehabilitation applied to vestibulo-cerebellar syndrome in presumed cerebellar hypoplasia: a case report

### **Diane Grosjean**^1^, David Levine^2,3^, Ashley Wheeler^3^

#### ^1^Ghent University, Faculty of Veterinary Medicine, Merelbeke, Belgium; ^2^University of Tennessee at Chattanooga, Chattanooga, Tennessee, USA; ^3^Veterinary Care, and Specialty Group, Chattanooga, Tennessee, USA

##### **Correspondence:** Diane Grosjean (diane.grosjean@ugent.be)

*Acta Veterinaria Scandinavica* 2023, **65(Suppl 1)**:O.05

**Background:** Cerebellar hypoplasia (CH) is a congenital neurologic condition caused by the inadequate development of the cerebellum which is responsible for fine-tuning motor movements [1]. This condition may lead to head tilt, ataxia, nystagmus, strabismus, and other neurological deficits, including an inability to move or walk normally for patients [2]. This pain-free condition remains stable, so the patient can live an acceptable quality of life if able to maintain basic functions such as eating, drinking, moving, and resting comfortably [3]. These dogs can usually learn to compensate for their condibilitation represents an option for those patients to improve their quality of life.

**Materials and methods:** A 4-month-old Australian shepherd was presented for rehabilitation management with presumed CH following his evaluation by a neurologist. Clinical presentation included: head tilt towards the left side, intermittent positional nystagmus, non-ambulatory severe ataxia, and increased reflexes and extensor tone in all limbs with a lack of tonus inhibition on the left side (Fig. 1). The owner’s goal was to improve gait function and body orientation to ensure optimal quality of life. The rehabilitation protocol included mobilizations, balance exercises, assisted walking, and underwater treadmill (UWTM) sessions. Emphasis was placed on the ability to turn left for this patient since this was his larger deficit, and he turned right almost exclusively. Multiple rehabilitation strategies were employed to teach him how to turn left including direct stimulation using voice, toys, and food, and indirect techniques using obstacles. Facilitators such as gait exercises on a mild down slope had a positive effect to help this patient turn left. Sessions were repeated daily for a duration of 20 min. A four-wheeled cart was prescribed to promote independent mobility and trunk stabilization; modifications to the cart included side cushioning and straps to prevent skin breakdown due to tremors and exaggerated movements (Fig. 2).

**Results:** A Bailey chair and a soft diet prescription were provided to promote a safe eating setup in the context of megaesophagus (Fig. 3). During UWTM sessions, a life vest adaptation was provided to hold the head in an upper position (Fig. 4).

**Figure 1 (abstract O.05) Figc:**
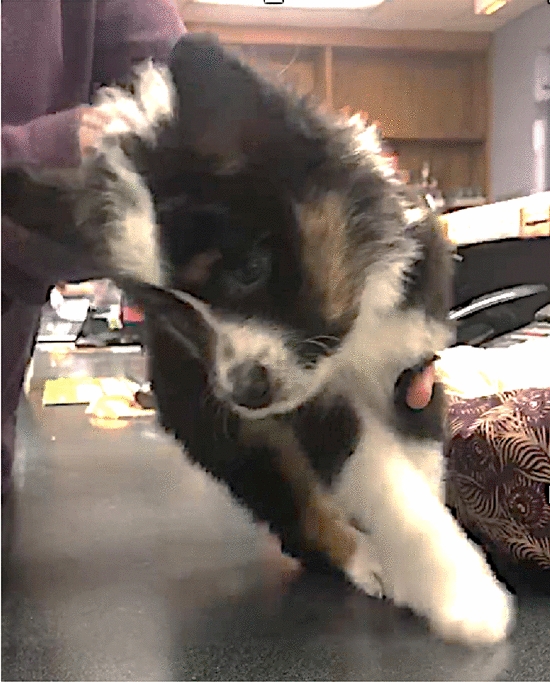
Initial neurologic evaluation

**Figure 2 (abstract O.05) Figd:**
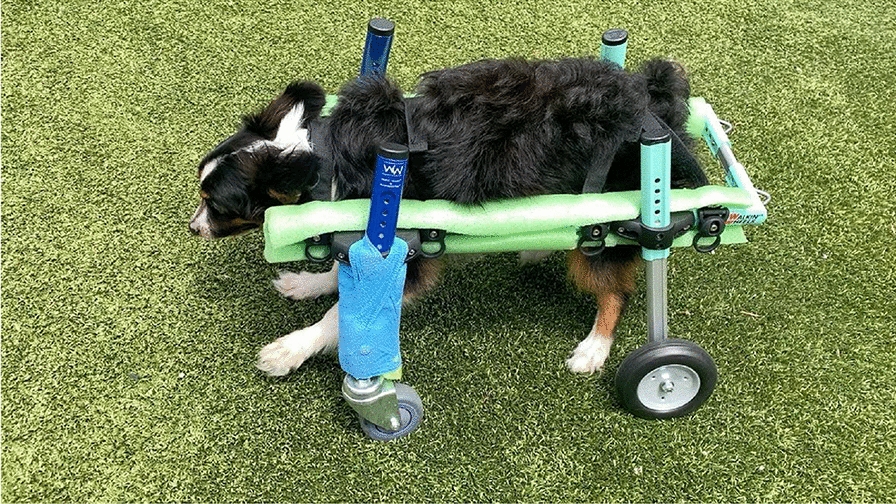
Cart improvements with straps and side cushioning

**Figure 3 (abstract O.05) Fige:**
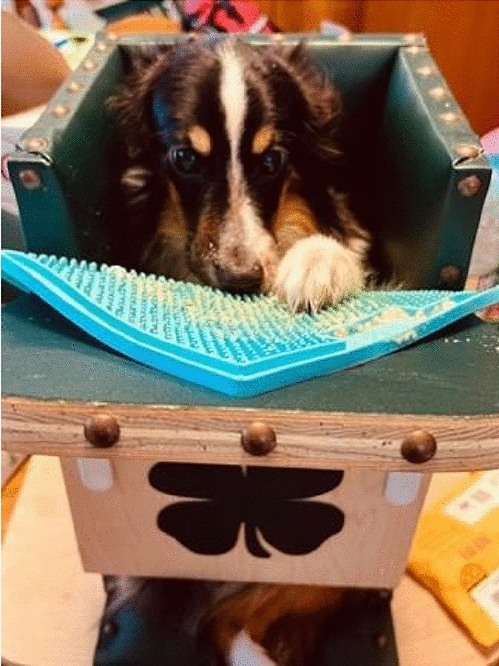
Bailey chair

**Figure 4 (abstract O.05) Figf:**
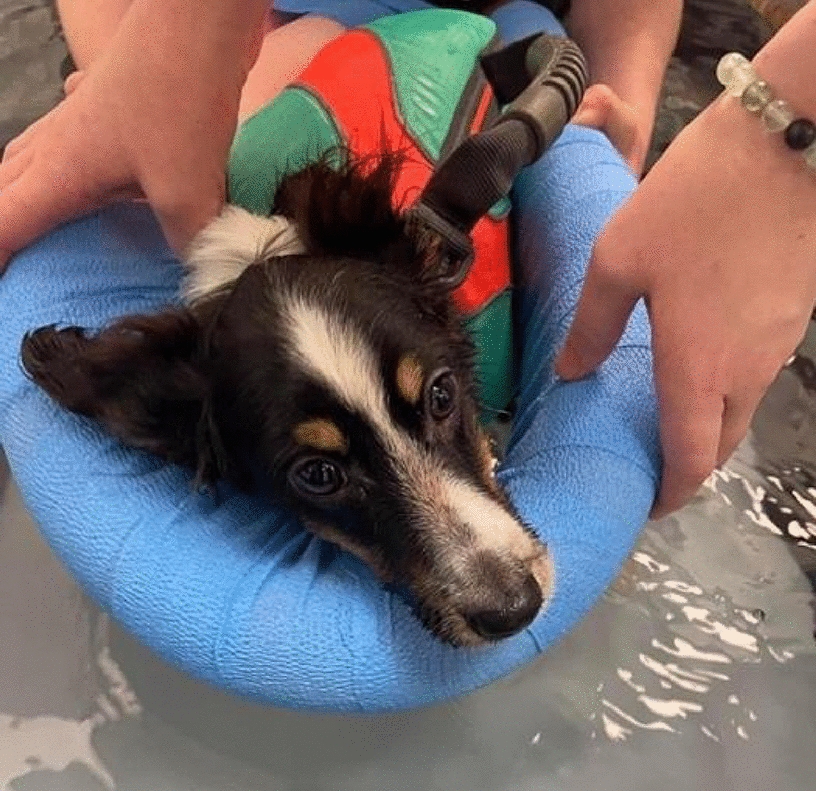
Life vest adaptations for head control

**Conclusion:** Outcome assessments gathered after 3 months of continuous rehabilitation plan included decreased head tilt, improved motor function, and independent ambulation achieved with a four-wheel cart for short distances (up to 15 feet). Therefore, rehabilitation should be considered for patients presenting CH to improve motor function and quality of life.


**References**
Dewey Curtis W, Costa Ronaldo C. Practical guide to canine and feline neurology. 3rd ed. Wiley Blackwell; 2016; 672Prikryl M, Caine A, Palus V. Transient postural vestibulo-cerebellar syndrome in three dogs with presumed cerebellar hypoplasia. Front Vet Sci. 2020;7:453.Mariani Christopher, Chrisman Cheryl, Platt Simon. Neurology for the small animal practitioner. Vet Clin North Am Small Anim Pract. 2002; 353.


## O.06 Evaluation of class IV laser treatment for the management of stifle osteoarthritis in dogs

### **Darryl Millis**^1^, Marti Drum^1^, Jennifer Carr^1^

#### ^1^Department of Small Animal Clinical Sciences, University of Tennessee, Knoxville, Tennessee, USA

##### **Correspondence:** Darryl Millis (dmillis@utk.edu)

*Acta Veterinaria Scandinavica* 2023, **65(Suppl 1)**:O.06

**Background:** Osteoarthritis (OA) of the stifle is common. Despite good conservative management, many dogs continue to suffer from pain associated with OA. Therapeutic laser is popular as a potential adjunct therapy, but there is little documentation of efficacy in clinical patients, although it reportedly reduces pain and lameness in arthritic elbow joints [1]. The purpose of this study was to evaluate the use of a Class IV laser on OA of the stifle using objective force platform evaluation. We hypothesized that daily treatment with a Class IV laser would reduce lameness and increase weight bearing compared to sham treatment in a paired crossover design.

**Materials and methods:** Nine dogs with experimentally induced stifle OA were used in this study. All dogs had surgical transection of one cranial cruciate ligament and immediate repair with either a lateral fabella tibial suture or a tibial plateau leveling osteotomy and were chronically affected with stifle OA for greater than 12 months. To qualify for the study, dogs must have had a difference of peak vertical force (PVF) between sides of > 5%. Dogs were randomized to receive either initial laser or sham treatments. Weekly ground reaction force measurements were made for 28 days. After the initial 28-day assessment, a 28-day wash-out period was given, and dogs switched groups. Measurements were again made weekly for another 28 days. Treatment with the laser or sham treatments were performed five out of seven days each week, using a 980 nm laser. Limbs were clipped, and the laser dosage was 10 J/cm^2^, using 3 watts of power.

**Results:** Statistical analysis using a 2-way ANOVA did not reveal any significant differences in peak vertical force between treatment and control groups (Table 1) or symmetry index between the two pelvic limbs over the 28-day study period (P > 0.05).

**Table 1 (abstract O.06) Taba:** Peak vertical force of dogs receiving laser or sham laser treatment 5 days per week for 4 weeks

	Day 0	Day 7	Day 14	Day 21	Day 28
Laser Treatment	58.3	58.6	61.7	62.1	61.7
Sham Treatment	59.5	58.2	60.0	62.8	61.5

**Conclusion:** Class IV laser therapy, used in the treatment protocol described here, did not provide benefits regarding improved ground reaction forces. Further studies evaluating different treatment protocols, including other doses or frequency of treatment, are warranted.


**Reference**
Looney AL, Huntingford JL, Blaeser LL, Mann S. A randomized blind placebo-controlled trial investigating the effects of photobiomodulation therapy (PBMT) on canine elbow osteoarthritis. Can Vet J 2018;59:959–66.


## O.07 A preliminary investigation into laterality differences of range of motion during flexion and extension of canine proximal limb joints

### **Suzy Soper**^1^, Sally Charlton^2^, Adrian Hunnisett^2^

#### ^1^Mctimoney Animal Association/Private practice, Tonbridge, United Kingdom; ^2^McTimoney College of Chiropractic, Abingdon, UK

##### **Correspondence: ** Suzy Soper (suzy@suzysoper.co.uk)

*Acta Veterinaria Scandinavica* 2023, **65(Suppl 1)**:O.07.

**Background:** The study's aim was to assess and compare left and right-side flexion and extension of the glenohumeral, humeroulnar/humeroradial, coxofemoral, and femorotibial joints and for laterality ROM differences. Research into musculoskeletal imbalances and differences between left and right-hand side joint ROM in *Canis familiaris* is limited [1]. Relationships between thoracic and pelvic limb structure and function have been observed, however, there is little peer-reviewed research [2]. Passive joint ROM provides proof that particular joints can move in their physiological planes of motion without the influence of muscle activity.

**Materials and methods:** Siberian huskies (n = 18) from one kennel were selected to minimize genetic and environmental effects. Dogs were of mixed gender (55.5% males (*n* = *10*) and 45.5% females (*n* = *8*)), aged > 1 year (mean ± S.D. (range)): 5.1 ± 3.2 (1.4–11.8). Joint ROM was measured using goniometry, a validated, non-invasive method in dogs [3], by the same investigator previously tested for acceptable repeatability of measurement. Dogs were conscious and placed in a standing position [4], specific bony landmarks were identified before triplicate measures of joint flexion and extension were taken on both sides of each dog for shoulder, elbow, hip, and stifle joints. Mean values of the measures were computed and tests Pearson Correlation Coefficient compared laterality of joint ROM, gender, and ages effects.

**Results:** There was no significant difference (P > 0.05) between left and right side flexion and extension measures. Gender had no significant effect (P > 0.05) on joint ROM measures for flexion or extension. Age (< 6yrs vs > 6yrs) had a significant effect on right hip flexion (P = 0.0009) and for both left and right sides for shoulder flexion (P = 0.0002 and P = 0.0004), elbow flexion (P = 0.001 and P = 0.0006), hip extension (P = 0.02 and P = 0.009) (Fig. 1). The shoulder joint showed greatest ROM asymmetry (SI = 3.63%). Joint asymmetry was minimal for elbow (SI = 0.1%), stifle (SI = 0.63%) and hip (SI = 1%) joints.

**Figure 1 (abstract O.07) Figg:**
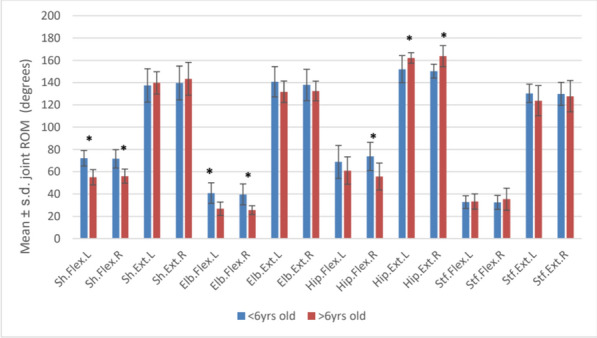
Bar chart of mean joint flexion and extension ROM for age groups (*denotes significance difference between groups)

**Conclusion:** Bilateral ROM measures of fore and hind quarters are important to consider in joint movement assessment. Joints can show different preferences in the asymmetry of passive ROM. This warrants further investigation with larger cohorts of defined age groups and could have implications in monitoring limb joint function of dogs.

## References


Adamokova J, Benediktova K, Svoboda J, Bartos L, Vynikalova L, Novakova P, et al. Turning preference in dogs: north attracts while south repels. PLoS One.2021;16:1Zink C, Schlehr, M. Working dogs structure: evaluation and relationship to function. Front Vet Sci. 2020; 7.Jaegger G, Dennis M, Levine D. Reliability of goniometery in Labrador Retrievers. Am J Vet Res. 2002; 63:979–86.Sabanci S, Ocal M. Comparison of goniometric measurements of the stifle joint in seven breeds of normal dogs. Vet Comp Orthop Traumatol. 2016; 29:214–9

## O.08 Bacterial contamination of the environment of veterinary rehabilitation clinics

### **Nick Millis**^**1**^, David Levine^1,3^, Darryl Millis^1^, Henry Spratt^2^

#### ^1^University of Tennessee, College of Veterinary Medicine; ^2^University of Tennessee at Chattanooga, Chattanooga, Tennessee, USA; ^3^Veterinary Care and Specialty Group, Chattanooga, Tennessee, USA

##### **Correspondence:** David Levine (David-Levine@utc.edu)

*Acta Veterinaria Scandinavica* 2023, **65(Suppl 1)**:O.08

**Background:** The presence of potentially pathogenic bacteria on surfaces in veterinary clinics is problematic for animals and people. Animals in these clinics often touch contaminated surfaces with their feet, noses, and mouths [1]. Fecal matter likely contaminates these surfaces, which presents the opportunity for shedding of *Clostridium perfringens* and *Clostridium difficile* [2]. There is evidence that bacterial strains originally thought to be pathogenic in animals, such as *Staphylococcus intermedius*, may also cause infections in humans [3]. Studies of Methicillin-resistant Staphylococcus aureus (MRSA) present the possibility of pets harboring MRSA contracted from humans that could lead to cross-transmission back to humans [4]. The focus of this study was to determine the contamination of environmental surfaces by potentially pathogenic bacteria in five veterinary rehabilitation clinics.

**Materials and methods:** Sampling involved using 30 double transport swabs (Fisherbrand, with Stuart’s liquid medium) from 13 different locations in each clinic (Table 1). The swabs were then placed on ice and transported to a microbiology lab (within three hours of collection) for processing. One of the two swabs was used to inoculate Hardy’s Cdiff Banana broth (for *Clostridium difficile*). The second swab was used to inoculate (line inoculation) Hardy CHROM MRSA agar (methicillin-resistant *Staphylococcus aureus* [MRSA], and *S. intermedius*), Mannitol Salt Agar (*S. aureus*), Eosin Methylene Blue agar (enterics), Pseudomonas Isolation Agar (*Pseudomonas aeruginosa*), and Tryptic Soy Agar. Water samples were diluted and spread directly onto the media.

**Results:** The most prominent species cultured from the clinics was *C. difficile* (Table 1). Enteric bacteria were the second most encountered bacteria, found on 33% of swabs from the sample sites. Both MRSA and *S. intermedius* were found on approximately 10% of swabs collected. Of the clinic sites sampled, the largest number of positive swabs was from the floor and air ventilation sites (HVAC) (Table 1). For water samples collected from the underwater treadmills, total bacterial counts ranged from an average of 380 to 2,880 CFU/ml.

**Table 1 (abstract O.08) Tabb:** Percentage of positive cultures on rehabilitation equipment and environment

Site	C. Diff	Enterics	SA	SIM	MRSA	PA
UWTM Belt	21.4	7.1	7.1	7.1	7.1	0.0
UWTM Jets	12.5	50.0	0.0	12.5	0.0	25
Exercise Equip	74.1	29.6	18.5	0.0	11.1	3.7
Floor	94.7	52.6	52.6	15.8	15.8	0.0
HVAC	83.3	100.0	83.3	16.7	33.3	0.0
US Heads	16.7	0.0	0.0	0.0	0.0	0.0
US Gel	25.0	0.0	0.0	0.0	0.0	0.0
Laser Probes	33.3	11.1	11.1	11.1	0.0	0.0
Life Jackets	60.0	40.0	0.0	10.0	0.0	0.0
Harnesses	75.0	12.5	25.0	25.0	0.0	0.0
Scale	66.7	83.3	83.3	33.3	50.0	16.7
Land Treadmill	80.0	40.0	40.0	0.0	40.0	0.0

C. Diff = *Clostridium difficile*, Enterics = Enterobacteriaceae, SA = Staphylococcus aureus, SIM = *Staphylococcus intermedius*, MRSA = Methicillin-resistant *Staphylococcus aureus*, PA = *Pseudomonas aeruginosa*

**Conclusions:**
*Clostridium difficile* was the most prominent bacterial species on environmental surfaces in these clinics, with clinic floors and HVAC systems having the highest levels of contamination. Despite posing few risks, these bacteria may be major pathogens for humans. *S. intermedius* tends to be more pathogenic for dogs, and notable levels of *Staphylococcus* species were present throughout the clinics sampled. Targeted cleaning and disinfecting, along with frequent monitoring of veterinary rehabilitation facilities, may reduce risks of infection in both animals and humans in clinics.


**References**
Murphy CP, Reid-Smith RJ, Boerlin P, et al.. *Escherichia coli* and selected veterinary and zoonotic pathogens isolated from environmental sites in companion animal veterinary hospitals in southern Ontario. Can Vet J. 2010;51:963–72.Álvarez-Pérez S, Blanco JL, Harmanus C, Kuijper EJ, García ME. Data from a survey of *Clostridium perfringens* and *Clostridium difficile* shedding by dogs and cats in the Madrid region (Spain), including phenotypic and genetic characteristics of recovered isolates. Data Brief. 2017;14:88–100Kelesidis T, Tsiodras S. *Staphylococcus intermedius* is not only a zoonotic pathogen but may also cause skin abscesses in humans after exposure to saliva. Int J Infect Dis. 2010;14:838–41.Morris DO, Lautenbach E, Zaoutis T, Leckerman K, Edelstein PH, Rankin SC. Potential for pet animals to harbour methicillin-resistant *Staphylococcus aureus* when residing with human MRSA patients. Zoonoses Publ Health. 2012;59:286–93.


## O.09 Preservation of temporospatial gait parameters in sound dogs fitted with a patient-specific stifle orthotic

### **Kirsten Häusler**^1^, Diane Messum^2^, Ben Blecha^3^, Katja Söhnel^4^, Matthew Allen^5^

#### ^1^Tierphysiotherapie Dr. Häusler &Team, Lambertweg 36, 70565 Stuttgart, Germany; ^2^Davies Therapy and Fitness Centre, Davies Veterinary Specialists, Manor Farm Business Park, Higham Gobion, Hitchin, SG5 3HR, UK; ^3^Hero Braces, 419 Eagle St., Benkelman NE 69021, United States; ^4^Friedrich-Schiller-Universität Jena, Institut für Zoologie und Evolutionsforschung, Erbertstraße 1, 07743 Jena; ^5^Department of Veterinary Medicine, Cambridge University, Cambridge, United Kingdom of Great Britain and Northern Ireland.

##### **Correspondence:** Kirsten Hausler (info@dr-haeusler.com)

*Acta Veterinaria Scandinavica* 2023, **65(Suppl 1)**:O.09

**Background:** Controversy remains over the potential benefits of orthotics for managing dogs with confirmed cranial cruciate ligament (CCL) rupture. The study aimed to investigate the effects of a patient-specific stifle brace on gait parameters in dogs.

**Materials and methods:** Five sound, skeletally mature Border Collies were analyzed at the walk and trot using an instrumented treadmill. Recordings were obtained at baseline (before placement of the orthotic); with the dog wearing the orthotic, and following removal of the orthotic. Gait parameters included lateral and anterior–posterior skewness, symmetry index, step length, and peak loading for each of the four limbs. The orthotic was fitted to the left hind limb on all dogs.

**Results:** When comparing walking with or without the brace, significant changes could only be detected for lateral and posterior-anterior skewness and maximum average load hindlimb on the right side before and while wearing the brace. Trotting showed highly significant changes for maximum average load hindlimb on both sides before, while, and after wearing the brace. The impact of the braces showed improved loading on the hind limbs and no changes to loading the front limb nor any symmetry changes.

**Conclusion:** Clinical trials in dogs with CCL rupture are now indicated. The data confirms that a custom-made orthotic can support an injured knee without limiting the step length or symmetry of the dog. No adverse or compensatory changes to the gait pattern could be identified.

## O.10 Back pain on epaxial muscle palpation as a risk factor for lameness elimination during endurance competitions

### **Fiona Bloom**^1^, Euan Bennet^2^, Stephen Draper^1^, Gillian Tabor^1^, David Marlin^3^, Jane Williams^1^

#### ^1^Hartpury University, Gloucester, UK; ^2^Glasgow University, Glasgow, UK; ^3^AnimalWeb Ltd, Cambridge, UK

##### **Correspondence:** Fiona Bloom (fiona.bloom@hartpury.ac.uk)

*Acta Veterinaria Scandinavica* 2023, **65(Suppl 1)**:O.010

**Background:** Lameness is the leading cause of elimination from endurance competitions [1–8], and within British national competitions, hindlimb lameness is more common than forelimb lameness [9]. Evidence supports the co-existence of thoracolumbar back pain and hindlimb lameness in horses [10]. This study aimed to identify if thoracolumbar back pain during endurance competition was a risk factor associated with elimination, more specifically lameness elimination, from competition.

**Materials and methods:** The study occurred across eight days of British national endurance competitions across five different venues in 2021. During the veterinary inspection, in addition to assessing lameness, when palpating the epaxial musculature, veterinarians were asked to grade the horses backs based on a categorical scale [11]. Pearson's chi-squared tests were used to identify significant differences between back palpation scores and successful or unsuccessful outcomes. Univariable models were constructed for risk factors for two results (a) eliminated vs. not eliminated and (b) lame vs. not lame. Risk factors were considered significant for multivariable analysis with a P value of ≤ 0.1. Multivariable logistic regression models were constructed using a backward stepwise process with an omnibus test of model coefficients applied at each step.

**Results:** Across all rides, 44 horses started with an asymmetrical back palpation score; of those, 29.5% (n = 13) were subsequently eliminated for lameness. In comparison, 8.5% (n = 32) of the horses which started with a symmetrical back palpation score (n = 377) were eliminated lame. A significant difference was found (P < 0.001) between horses who palpated a score of 0–2/5 and horses who palpated a higher score of > 3/5, whether they were eliminated or not and whether they were lame or not. Horses who presented with an asymmetrical back palpation score throughout the ride were at increased odds of elimination from the competition (Odds ratio 2.31, 95% CI 1.15–4.62, P = 0.018) and increased odds of lameness (Odds ratio 4.16, 95% CI 1.92–9.01, P < 0.001).

**Conclusions:** Asymmetrical back palpation scores during endurance competitions are a significant risk factor for elimination, specifically lameness elimination during British national endurance competitions. Higher palpation scores, associated with pain response, were found to be significantly different in terms of elimination and lameness when compared to palpation scores considered normal or slightly hypertonic. This indicates back pain in endurance horses during competition should be further investigated to minimize the risk of lameness eliminations and optimize welfare.


**References**
Nagy A, Murray J Dyson S. Elimination from elite endurance rides in nine countries: a preliminary study. Equine Vet J Suppl. 2010; 38:637–43Fielding CL, Meier CA, Balch OK, Kass PH. Risk factors for eliminating endurance horses from competition. J Vet Med Educ. 2011; 239:493–8.Nagy A, Dyson SJ, Murray JK. A veterinary review of endurance riding as an international competitive sport. Vet J. 2012, 194: 288–93.Nagy A, Murray JK, Dyson SJ. Descriptive epidemiology and risk factors for elimination from Fédération Equestre Internationale endurance rides due to lameness and metabolic reasons (2008–2011). Equine Vet J Suppl. 2014; 46:38–44Younes M, Barrey E, Cottin F, Robert C. Elimination in long-distance endurance rides: insights from the analysis of 7032 starts in 80-160 km competitions. Comparative Exercise Physiology 2016; 12: 157–167Nagy A, Dyson SJ, Murray JK. Veterinary problems of endurance horses in England and Wales. Prev Vet Med 2017; 140: 45–52.Bennet ED, and Parkin TDH. Fédération Equestre Internationale endurance events: risk factors for failure to qualify outcomes at the level of the horse, ride, and rider (2010–2015). Vet J. 2018; 236: 44–48.Bennet ED, Parkin TDH. Fédération Equestre Internationale endurance events: riding speeds as a risk factor for elimination (2012–2015). Vet J. 2018; 236:37–43:Bloom F, Draper, S, Bennet E, Marlin D, Williams J. A description of veterinary eliminations within British national endurance rides in the competitive season of 2019. Comparative Exercise Physiology 2022;18:1–10.Landman MA, de Blaauw JA, van Weeren PR, Hofland LJ. Field study of the prevalence of lameness in horses with back problems. Veterinary Record 2004; 155: 165–8Merrifield-Jones M, Tabor G. Williams J. Inter- and intra-rater reliability of soft tissue palpation scoring in the equine thoracic epaxial region. J Eq Vet Sci 2019; 83.


## O.11 Inter-rater reliability of grading soft tissue palpation of the thoracolumbar epaxial musculature of endurance horses during competition

### **Fiona Bloom**^**1**^, Euan Bennet^2^, Stephen Draper^1^, Gillian Tabor^1^, David Marlin^3^, Jane Williams^1^

#### ^1^Hartpury University, Gloucester, UK; ^2^Glasgow University, Glasgow, UK; ^3^AnimalWeb Ltd, Cambridge, UK

##### **Correspondence:** Fiona Bloom (fiona.bloom@hartpury.ac.uk)

*Acta Veterinaria Scandinavica* 2023, **65(Suppl 1)**:O.011

**Background:** During an endurance competition, horses must pass a series of veterinary inspections to complete the ride [1, 2]. Within these veterinary inspections, the horse’s epaxial musculature is palpated. There have been no studies looking at the inter-rater reliability of back palpation during any equestrian competition. This study aimed to test the inter-rater reliability of back palpation during an endurance competition using a categorical grading scale [3].

**Materials and methods:** Nineteen horses of mixed breeding entered into a pleasure ride (13–24 km) run by Endurance GB were included in the study. All horses were presented to a veterinary inspection consisting of a trot in hand, 30 m away from and back to two licensed veterinarians. Having been declared fit to start the competition, the horses had their thoracolumbar epaxial musculature palpated by the veterinarian. They graded based on the scale described by Merrifield-Jones et al. [3]. The veterinarian gave their palpation score for both left and right sides of the epaxial musculature to a scribe. A fourth-year veterinary student blinded from the veterinary surgeon’s scores then palpated the musculature and gave their scores to the recorder. The horses completed their ride (13–34 km), and the veterinary inspection, including epaxial palpation, was repeated. Inter-rater reliability of palpation scores was tested with a Fleiss’ kappa analysis series.

**Results:** At the start of the competition, inter-rater reliability between veterinarian 1 and the veterinary student was significant and ‘moderate’ K = 0.60 (95% CI 0.59- 0.6) P = 0.004). The agreement between veterinarian 2 and the student at the start was significant K = 0.72 (95% CI 0.71–0.73, P < 0.001). At the end of the ride, the veterinary student had a total agreement with each of the veterinary surgeons, K = 1.00 (95% CI 0.99–1.01, P < 0.001). Overall, the agreement between veterinarian 1 and the veterinary student was excellent K = 0.89 (95% CI 0.88–0.89), P < 0.001. The overall agreement between veterinarian 2 and the veterinary student was also excellent, K = 0.82 (95% CI 0.81–0.83), P < 0.001.

**Conclusion:** This study identified that the categorical rating scale used for manual palpation of the equine epaxial musculature during endurance competition has excellent inter-rater reliability between veterinary surgeons and veterinary students. To establish the validity of the scale during competition, further information and testing is necessary.


**References**
Endurance GB Rule Book. 2022; https://egb.myclubhouse.co.uk/Cms/Spaces/RULES/01+General+and+FAQs; accessed 08 March 2022.Fédération Equestre Internationale. FEI Endurance Rules 2022. Available at: https://inside.fei.org/fei/disc/endurance/rules; accessed 06 April 2022.Merrifield-Jones M, Tabor G, Williams J. Inter- and intra-rater reliability of soft tissue palpation scoring in the equine thoracic epaxial region. J Equine Vet Sci. 2019; 83:102812. https://doi.org/10.1016/j.jevs.2019.


## O.12 Osteopathic sacroiliac joint manipulation improves locomotion in horses with SI dysfunction

### **Toni Ramon**^1^, Marta Prades^1^, Constanza Gómez Álvarez^2^

#### ^1^Universitat Autònoma de Barcelona, Catalunya, Spain; ^2^University of Cambridge, Cambridge, UK

##### **Correspondence:** Toni Ramon (toni@fisiovet.com)

*Acta Veterinaria Scandinavica* 2023, **65(Suppl 1)**:O.012

**Background:** Pelvic dysfunctions are commonly treated by osteopaths. There have been a few published studies on the effects of osteopathic treatment on equine backs, but not on the effects of osteopathic therapy of pelvic problems.

The hypothesis of our study is that osteopathic treatment of Sacroiliac joint (SI) dysfunction would improve locomotion, especially during walk and canter.

**Material and methods:** 21 patients presenting with an osteopathic blockage of one or both (SI) were treated using thrust techniques. Eight patients were in the control group. Videos of all horses were clinically evaluated before the treatment, immediately after, at 3 days, and at 15 days by two blinded veterinarians, and by means of 9 Equimoves® inertial sensors to evaluate gait parameters. Scores were given for canter quality (ability to maintain the canter in circles, taking off with the correct trailing hind limb, switching leads or not and presence of 3 beat canter).

**Results:** Significant improvement was noticed in pain scores of SI area P < 0.001 and in both hind limb lameness scores (right hindlimb P = 0.009 and left hindlimb P < 0.001) in the trot at lunge. Quality of galop also improved (evaluated by correct takeoff score: to the right P = 0.017 and left P = 0.038 and ability to maintain canter in circles P = 0.007). This was corroborated by objective gait parameters measured with Equimoves® inertial sensors.

**Conclusion:** Treated horses improved their locomotion, especially on day 3, and the effects were maintained for at least 15 days.

## O.14 A case series of 11 horses diagnosed with bone spavin treated with high-intensity laser therapy (HILT)

### **Paulina Zielińska**^**1**^ Maria Soroko^2^, Karolina Śniegucka^1^

#### ^1^Department of Surgery, Wroclaw University of Environmental and Life Sciences, Wroclaw, Poland; ^2^Institute of Animal Breeding, Wroclaw University of Environmental and Life Sciences, Wroclaw, Poland

##### **Correspondence:** Paulina Zielińska (paulina.zielinska@upwr.edu.pl)

*Acta Veterinaria Scandinavica* 2023, **65(Suppl 1)**:O.014

**Background:** Bone spavin (BS) is chronic tarsus osteoarthritis that involves the tarsometatarsal joint and the distal intertarsal joint with involvement of the proximal intertarsal joint [1]. Horses can suffer from bone spavin and it is the most common cause of hind limb lameness [2]. HILT is able to bring a high amount of light energy to deep tissues in a short time [4], and stimulate areas such as the large joint [5]. HILT has been used for pain management in human musculoskeletal disorders such as osteoarthritis [6].

**Materials and methods:** Case records of 11 horses of mean age 12.5 ± 2.7 years diagnosed with BS were included in the study. None of the horses had been treated or shoed for BS in the last 6 months. Involved tarsal joints were treated using class IV laser with 808 nm and 980 nm wavelengths delivered simultaneously. Each horse received 10 treatments during a 14-day period with the same HILT parameters. Pre-treatment and post-treatment orthopedic and radiographic examinations were performed to assess changes in lameness grade, spavin test grade, and bone structure. Lameness was scored using the American Association of Equine Practitioners 0–5 scale [7] and spavin test was scored according to Stashak [8].

**Results:** At post-treatment orthopedic examination 36% of the horses had improved two lameness grades, 36% of the horses had improved one lameness grade and 28% of the horses did not show any improvement (Fig. 1). For post-treatment spavin test results one grade improvement was observed in 45% of the horses and 55% of the horses showed the same spavin test grade as before treatment (Fig. 2). Follow-up radiographs when compared to initial radiographs did not show any significant changes.

**Figure 1 (abstract O.14) Figh:**
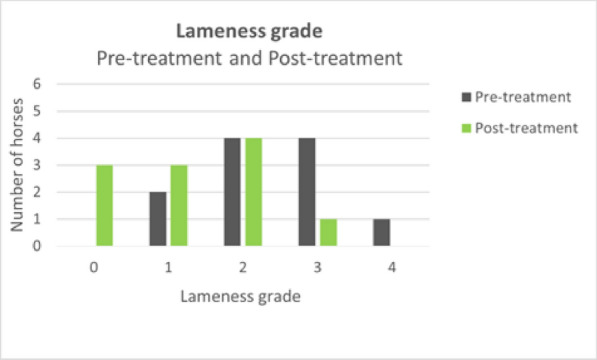
The lameness grade for each horse was assessed in pre-treatment and post-treatment orthopedic examinations

**Figure 2 (abstract O.14) Figi:**
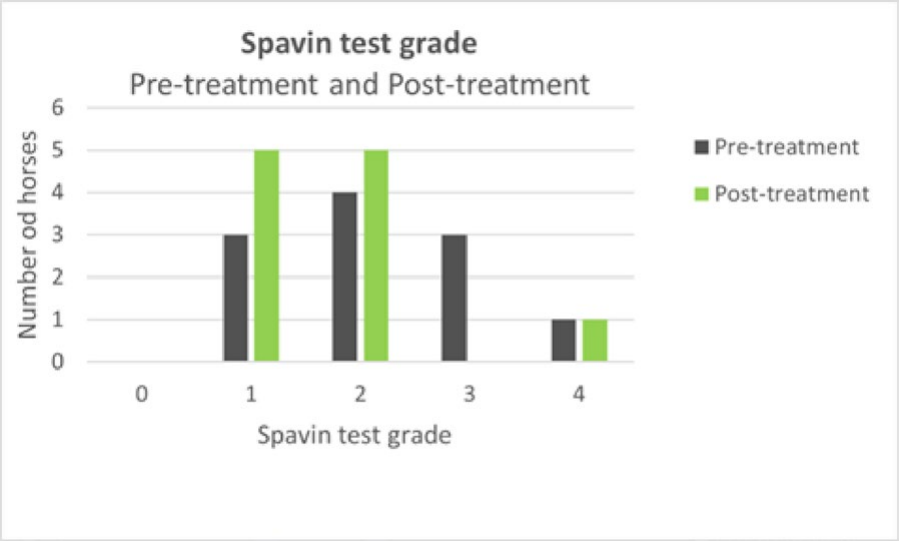
The spavin test grade for each horse was assessed in pre-treatment and post-treatment orthopedic examination

**Conclusions:** HILT reduces joint pain and lameness grade but poorly limits joint discomfort after a flexion test in horses suffering from BS. The case series suggest that it is too early to determine if HILT is good as an alternative, non-invasive and non-pharmacological agent in the management of chronic joint pain in horses. The outcome can help in obtaining more insight into the photobiostimulation effect of HILT on horse joint pain accompanying osteoarthritis.


**References**
Adams OR. Surgical arthrodesis for treatment of bone spavin. J Am Vet Med Assoc 1970;157:1480–5.Rose R, Hodgson D. Bone spavin. In: Manual of Equine Practice. W.B. Saunders, Philadelphia; 1993; 113–4.Fortuna D. High-intensity laser therapy for the equine patient. In Laser Ther in Vet Med Photobiomodulation. 2017;415–21.Boyraz I, Yildiz A, Koc B, Sarman H. Comparison of high-intensity laser therapy and ultrasound treatment in the patients with lumbar discopathy. Biomed Res Int.; 2015:304–28.Santamato A, Solfrizzi V, Panza F, Tondi G, Frisardi V, Leggin BG, et al. Short-term effects of high-intensity laser therapy versus ultrasound therapy in the treatment of people with subacromial impingement syndrome: a randomized clinical trial. Phys. Ther. 2009;89:643–52.Kim SH, Kim YH, Lee HR, Choi YE. Short-term effects of high-intensity laser therapy on frozen shoulder: a prospective randomized control study. Man. Ter. 2015;20:751–7.AAEP. Guide to veterinary services for horse shows, 7th ed.; AAEP: Lexington, KY, USA, 1999.Stashak TS. The tarsus in: Diagnosis of lameness. In: Adams’ Lameness in Horses, 4th edition, Ed: Lea and Febiger, Philadelphia: USA, 1987; 694–704.


## O.15 Association between clinical signs of back pain and epaxial muscle asymmetry in flat and national hunt racehorses

### Stella Zhang^1^, **Catherine McGowan**^1^

#### ^1^University of Liverpool, Leahurst, United Kingdom

##### **Correspondence:** Catherine McGowan (cmcgowan@liv.ac.uk)

*Acta Veterinaria Scandinavica* 2023, **65(Suppl 1)**:O.015.

**Background:** The *mm multifidus* cross-sectional area is decreased on the side and level of symptoms or pathology [1, 2]. Muscle asymmetry, coupled with clinical signs that are level- or side-specific, could provide vital information about the presence of undetected symptomatic pathology [3]. The study aimed, to define the prevalence of asymmetry in Flat and National Hunt Racehorses; secondly, to determine if this differed between disciplines. Finally, to determine if there is a relationship between asymmetry and evidence of back pain or dysfunction indicative of presumptive underlying spinal pathology.

**Materials and methods:** Thoroughbred racehorses in full training from Flat and National Hunt trainers were recruited. Injured, resting, or recently retired horses were excluded. Clinical assessment of the back included observation (evidence of saddle misfit, conformation, and epaxial muscle atrophy), palpation, and spinal range of motion. Horses were assigned a back pain score and clinical assessment cumulative score each. A Flexible Curve Ruler was used to measure thoracolumbar shape at T13, T18, and L4. Each shape was traced, photographed, and imported into an image analysis program. Each side was traced three times, the mean of these being used to calculate the percentage difference between left and right sides at each vertebral level. Horses that fell between 0 and 2.99% were considered symmetrical; ≥ 3% was considered asymmetrical.

**Results:** 178 Thoroughbreds (n = 161 Flat; n = 17 National Hunt Racehorses) were recruited. The prevalence of left-to-right epaxial muscle asymmetry at each level is described below (Table 1). At T18, more Flat Racehorses than National Hunt Racehorses were asymmetrical (42.86%; [95% CI 0, 35.30] vs. 17.65%; [95% CI 35.40, 50.30]; P < 0.04, respectively). There were no significant differences between discipline at T13 and L4, or in the distribution of back pain scores (i.e., no back pain vs. back pain), and cumulative scores (i.e., normal horses vs. horses with clinical signs of back dysfunction /pathology), between horses classified as symmetrical compared to asymmetrical at T13, T18 or L4.

**Conclusion:** Asymmetry cannot be used as a standalone marker to determine the presence of current or potential symptomatic pathology. It is likely a large percentage of racehorses will have incidental pathological spinal findings; it cannot be assumed that every pathology is or will be, symptomatic or clinically significant [2, 4]. Asymmetry measurements coupled with clinical examination should form part of a battery of functional and performance tests of individual racehorses.

**Table 1 (abstract O.15) Tabc:** Percentage of horses (n = 178) showing left/right epaxial muscle asymmetry

	T13 T18 L4					
	%	95% CI	%	95% CI	%	95% CI
Symmetrical	62.90	[55.10, 69.70]	59.60	[52.80, 66.90]	55.60	[47.80, 62.90]
Asymmetrical	37.10	[30.30, 44.90]	40.40	[33.10, 48.30]	44.4	[37.10, 51.70]


**References**
Hides J, Gilmore C, Stanton W, Bohlscheid E. Multifidus size and symmetry among chronic LBP and healthy asymptomatic subjects. Man Ther. 2008; 1:43–9.Stubbs NC, Riggs CM, Hodges PW, Jeffcott LB, Hodgson DR, Clayton HM. Osseous spinal pathology and epaxial muscle ultrasonography in Thoroughbred racehorses. Equine Vet J. 2010; 42:654–61.Battié MC, Niemelainen R, Gibbons LE, Dhillon S. Is level- and side-specificmultifidus asymmetry a marker for lumbar disc pathology? Spine J. 2012; 12:932–9.Kasch R, Truthmann J, Hancock MJ, Maher CG, Otto M, Nell C. Association of lumbar MRI findings with current and future back pain in a population-based cohor study. Spine. 2022; 47:201–11.


## PO. 01 Physical activity and sport-specific training patterns in Swedish working trial dogs; a questionnaire survey

### **Ann Essner**^1,2^, Amie Hesbach^3^, Helena Igelström^2^, Catarina Kjellerstedt^4^, Kristina Svensson^5^, Helga Westerlind^6^

#### ^1^Djurkliniken Gefle, IVC Evidensia, Gävle, Sweden; ^2^Department of Women’s and Children’s Health, Uppsala University, Uppsala, Sweden; ^3^EmpowerPhysio, The Hague, The Netherlands; ^4^Veterinär Catarina Kjellerstedt, Vallentuna, Sweden; ^5^Tolleruds Gård 116, Karlstad, Sweden; ^6^Clinical Epidemiology Division, Department of Medicine Solna, Karolinska Institutet, Stockholm, Sweden

##### **Correspondence:** Ann Essner (ann.essner@evidensia.se)

*Acta Veterinaria Scandinavica* 2023, **65(Suppl 1)**:PO. 01

**Background:** Physical requirements vary among working dog trial disciplines, where bite work (e.g., protection) involves tasks that require muscle strength and power [1–3]. Other working trials (e.g., searching for people) demand cardiorespiratory and muscular endurance [4–6]. Physical activity, including exercise, and sport-specific training, prepares dogs for the requirements of sporting and working tasks to reach full athletic performance and possibly decrease the risk of injury [7, 8]. This study aimed to explore physical activity and sport-specific training patterns among Swedish dogs participating in working trials.

**Materials and methods:** Dog handlers provided information on competition-level dogs through an internet-based survey on physical activity, sport-specific training, and management variables.

**Results:** We received 1615 replies to the questionnaire. After data cleaning, 1582 dogs (98%) were left for analysis. Of these, 847 dogs had competed in working trials, i.e., messenger, protection, search, and tracking. The vast majority of the dogs (n = 589, 70%) received more than one hour of physical activity, e.g., walks, per day, and only 4 (2.5%) were never exercised off-leash. Preferred self-selected gait for the dogs was trot (n = 478, 56%) and gallop (n = 274, 32%). About a fourth (n = 225, 27%) of the dogs never played with other dogs. Most dogs (n = 722, 85%) received more than one hour of vigorous physical exercise per week. Three-quarters of the participants (n = 602, 71%) added conditioning, i.e., cardiorespiratory, musculoskeletal, or a combination thereof. Two-thirds of the dogs (n = 565, 67%) participated in at least three hours of sport-specific training per week, and only a small portion (n = 8, 1%) trained in their specific sport less than once per week. The median total workload for the dogs, i.e., daily exercise, vigorous exercise, and sport-specific training, was 16.8 h per week. Of the dogs practicing one discipline, 5.2% (n = 83) were considered specialized since they actively trained in that one discipline for ≥ 10 months per year.

**Conclusion:** Our findings provide valuable insights into cultural differences between Swedish working trial dogs and previous studies on agility dogs; based on the physical activity and sport-specific training patterns, the working trial dogs in Sweden were moderate to highly active. Further studies regarding the risk and protective factors of injuries in competing working dogs are required.


**References**
Zink MC, Dyke JBV. Canine sports medicine and rehabilitation. 1st ed. Hoboken, NJ, USA: Wiley-Blackwell; 2013.Söhnel K, Rode C, de Lussanet MHE, Wagner H, Fischer MS, Andrada E. Limb dynamics in agility jumps of beginner and advanced dogs. J Exp Biol. 2020;223.Hyytiäinen HK, Blomvall L, Hautala M, Lappalainen AK. Reliability of a new bite force measure and biomechanics of modified long attack in police dogs. Animals (Basel). 2021;11:874.Diverio S, Barbato O, Cavallina R, Guelfi G, Iaboni M, Zasso R, et al. A simulated avalanche search and rescue mission induces temporary physiological and behavioral changes in military dogs. Physiol Behav. 2016;163:193–202.Rovira S, Munoz A, Benito M. Effect of exercise on physiological, blood and endocrine parameters in search and rescue-trained dogs. Vet Med. 2008;53:333–46.Spoo JW, Zoran DL, Downey RL, Bischoff K, Wakshlag JJ. Serum biochemical, blood gas, and antioxidant status in search and rescue dogs before and after simulated fieldwork. Vet J. 2015;206:47–53.


## PO. 02 Sport-specific training patterns and specialization amongst Swedish sporting and working trial dogs – a questionnaire survey

### **Ann Essner**^1,2^, Amie Hesbach^3^, Helena Igelström^2^, Catarina Kjellerstedt^4^ Kristina Svensson^5^, Helga Westerlind^6^

#### ^1^IVC Evidensia, Djurkliniken Gefle, Gävle, Sweden; ^2^Department of Women's and Children's Health, Uppsala University, Uppsala, Sweden; ^3^EmpowerPhysio, The Hague, Netherlands; ^4^Veterinär Catarina Kjellerstedt, Vallentuna, Sweden; ^5^Tolleruds Gård 116, Karlstad, Sweden; ^6^Clinical Epidemiology Division, Department of Medicine Solna, Karolinska Institutet, Stockholm, Sweden

##### **Correspondence:** Ann Essner (ann.essner@evidensia.se)

*Acta Veterinaria Scandinavica* 2023, **65(Suppl 1)**:PO. 02.

**Background:** Knowledge regarding specific training towards various disciplines and specialization in sporting and working trial dogs is lacking. This study aimed to explore sport-specific training patterns among Swedish dogs competing in agility, obedience, rally obedience, and working trial disciplines.

**Materials and methods:** Dog handlers provided information on competition-level dogs through an internet-based survey on physical activity, sport-specific training, and management variables.

**Results:** We received 1615 replies to the questionnaire. After data cleaning, 1582 dogs (98%) were left for analysis. The number of sports performed by each dog varied from one to five. Most common was participation in one (n = 762, 48%) or two (n = 548, 29%) sports). Three dogs competed in five sports disciplines, 51 dogs in four disciplines, and 222 dogs (14%) in three disciplines. Of the dogs practicing only one discipline, 39% (n = 297) were considered specialized as they actively trained in that discipline for ≥ 10 months per year. Similar proportions of working dogs (n = 83, 5.2%) and agility dogs (n = 83, 5.4%) were specialized. These proportions decreased slightly among obedience dogs (n = 65, 4.1%) and rally obedience dogs (n = 60, 3.8%).

**Conclusion**: Our findings provide valuable insights into sport-specific training patterns among active sports dogs in Sweden. Sport specialization may positively and negatively affect movement skills. Further studies regarding the risk and protective factors of injuries in the sporting and working dogs are required.

## PO. 03 Factors affecting the incidence of injury in agility dogs

### **Jenny Coates**^1^

#### ^1^School of Veterinary Science and Medicine, University of Nottingham, UK

##### **Correspondence:** Jenny Coates (jenny.coates@nottingham.ac.uk)

*Acta Veterinaria Scandinavica* 2023, **65(Suppl 1)**: PO. 03

**Background:** Dog agility is growing in the UK; as the sport progresses and agility dogs perform closer to their physical limits, the risk of injury increases. There is little research on the mechanisms of injury and the factors that influence whether a dog will sustain an injury at some point in their career. Owners are currently not encouraged to make choices that prevent injury. To shed light on potential causative factors, identify future avenues of investigation, and assist owners with increasing the physical resilience of their dog, this study aims to investigate variables that impact on the incidence of injury. Injuries may have a detrimental effect on the dogs’ agility careers as injured dogs will miss training and competition during the rehabilitation and recovery phase [1], and there is no guarantee that they will fully return to sport.

**Materials and methods:** A retrospective internet-based survey collected data from 280 owners of 357 dogs in the UK. The data was analyzed using a Pearson Chi-Squared test via two-by-two contingency tables to calculate the relative risk of injury for different breeds and the age at which agility training commenced.

**Results:** Soft tissue injuries are more common in agility dogs than joint injuries, with the shoulder area most vulnerable to damage. Border Collies have an increased risk of sustaining an injury compared with other breeds but are less likely to injure their shoulder area. Dogs who began their agility preparation in the age range of 5 to 10 months compared with dogs of the age range of 20 to 25 months experienced a reduced risk of injury.

**Conclusion:** To impact the incidence of injury, more research is needed in this area to ascertain the mechanics of how dogs negotiate an agility course. Owners, Veterinary Surgeons, and other members of the multidisciplinary team may have a positive effect on injury reduction by increasing the length of time spent on the physical preparation of the dog before starting the competition.


**Reference**
Levy I, Hall C, Trentacosta N, Percival M. A preliminary retrospective survey of injuries in dogs participating in canine agility. Vet Comp Orthop Traumatol. 2009; 22: 321–24.


## PO. 04 Improvements in pain and stiffness in dogs with osteoarthritis using a therapeutic dog mattress

### **Matthew Brunke**^1^, Kimberly Christie^1^, Jennifer Barnhard^1^, Heather Scavello^2^

#### ^1^Veterinary Surgical Centers Rehabilitation, Vienna, Virginia, USA; ^2^University of Pennsylvania, School of Veterinary Medicine, Veterinary Clinical Investigations Center, Philadelphia, Pennsylvania, ISA

##### **Correspondence:** Matthew Brunke (drmattbrunke@gmail.com)

*Acta Veterinaria Scandinavica* 2023, **65(Suppl 1)**:PO. 04

**Background:** Osteoarthritis is a chronic, debilitating condition that can affect the quality of life in dogs [1]. Previous studies have shown improved overall comfort with a modified foam mattress [2]. To the authors’ knowledge, this has not been studied in canines. This study objected to determine if the therapeutic mattress improved joint mobility, increased activity, and enhanced the quality/quantity of sleep in dogs with confirmed osteoarthritis.

**Materials and methods:** 40 dogs over 3 years old were enrolled in the study. Veterinary examination and radiographs were used to confirm osteoarthritis. Client-specific outcomes were obtained, with activity monitors placed ten days before the initiation of the mattress to establish baseline activity. The dogs returned on day 0, and the therapeutic bed was initiated. Dogs returned for evaluation on Day 28. Clients completed the Canine Orthopedic Index (COI), Canine Brief Pain Inventory (CBPI), Global Assessment of Change questionnaire (GAC), and Canine Symptom Assessment Scale (CSAS) [3].

**Results:** 40 dogs, 17 females, and 23 males, were included in the study analysis. The mean age for the dogs was 8.48 years (range from 3 to 12 years). The mean body weight was 41.1 kg (94.5 lbs.) with a capacity of 30.2 kg (66.4 lbs.) to 83.2 kg (183 lbs.). The most represented breeds were mixed breed (n = 14) and Labrador Retrievers (n = 7). The COI questionnaire evaluated the stiffness, gait, function, and quality of life of the participant dogs. All domain assessed scores were compared using t-tests, or Wilcoxon signed rank depending on the data type and were statistically significant ( P < 0.05). Owners reported a 12.5% improvement in joint stiffness and a 17.6% improvement in joint function. A modification in gait and the quality of life was also noted at 9.6% and 15.1%, respectively. Pain severity, pain interference, and quality of life were assessed through the use of the CBPI questionnaire. A 21.6% improvement was noted for pain severity, as well as a 14.3% improvement for pain interference. The activity monitor data did not show a statistically significant change compared to the baseline.

**Conclusion:** Owners of osteoarthritic dogs that had access to the therapeutic mattress for 28 days reported significant improvement in their dog’s joint stiffness, joint function, gait, pain, pain severity, pain interference, pacing, panting, and quality of life.

## Acknowledgments

The authors wish to thank Kimberly Maciejczyk, DVM, for assistance with statistical analysis.


**References**
1. Sanderson RO, Beata C, Flipo RM, Genevois J-P, Macias C, Tacke S, et al. Systematic review of the management of canine osteoarthritis. Vet Rec. 2009;164:418–24. https://doi.org/10.1136/VR.164.14.4182. Brown DC, Boston RC, Coyne JC, Farrar JT. Ability of the canine brief pain inventory to detect response to treatment in dogs with osteoarthritis. J Am Vet Med Assoc. 2008;233:1278–83; https://doi.org/10.2460/javma.233.8.12783. Bredesen IM. Interface pressures of new and worn standard and viscoelastic hospital mattresses: a comparative study. Wound Manag Prev. 2020;66:26–31.


## PO. 05 Profile identification of Canicross practitioners and their dogs in Brazil

### **Daniela Henrique**^1^, Moisés Martins^1^, Julia Lima^2^, Willian Oliveira^3^, Luís Murgas^1^

#### ^1^Department of Veterinary Medicine, Federal University of Lavras, Lavras, Brazil; ^2^Animal World Clinic, Varginha, Brazil; ^3^Founding Member of the Brazilian Association of Canicross and Similar Sports, Rio de Janeiro, Brazil

##### **Correspondence:** Daniela Henrique (daniela@pataepatela.com.br)

*Acta Veterinaria Scandinavica* 2023, **65(Suppl 1)**:PO. 05

**Background:** Canicross, a cross country race practiced by a man-dog pair, is a relatively new sport in Brazil and is attracting more and more fans. The study analyzed the profile of Canicross practitioners in Brazil. A semi-structured questionnaire consisting of 33 questions was used: seven related to the handler and 26 to the dogs.

**Materials and methods:** A semi-structured questionnaire consisting of 33 questions was used: seven related to the handler and 26 to the dogs.

**Results:** Of the total handlers, 53.3% are women, 64% have practiced the sport for at least 5 years and 52.3% trained with a personal trainer. Of the dogs, 62.6% were females, 60% were between one and four years old and the Mixed Breeds were the majority with 34.6%; as for the health of the animals, 80% of the handlers stated that they did not carry out veterinary monitoring aiming at Canicross. 6.6% of the animals had sports-related injuries and 80% of these cases received veterinary treatment. The percentage of animals that were not subjected to any type of specialized monitoring aiming at the sport was quite high (80%).

**Conclusion:** Handlers are concerned with the prevention and treatment of injuries and that these precautions are widely known, this is not reflected when it comes to dogs. More studies should be carried out, but the originality of this work was essential for both sports lovers and veterinary professionals to develop strategies so that dogs can practice this sport with more safely.

## PO. 06 Exploring the information and support needs of owners caring for a dog with canine cognitive dysfunction

### Daisy de Meester^1^, **Gillian Tabor**^1^

#### ^1^Hartpury University, United Kingdom

##### **Correspondence:** Gillian Tabor (gillian.tabor@hartpury.ac.uk)

*Acta Veterinaria Scandinavica* 2023, **65(Suppl 1)**:PO. 06

**Background:** Canine cognitive dysfunction (CCD) is an age-related neurodegenerative disease, sharing similar symptoms to Alzheimer’s disease in humans [1]. While it is recognized that CCD has a large impact on the owner and the dog [2], there is a lack of research identifying support and information provided to those caring for dogs with CCD and its effectiveness in enabling owners to provide care. The objectives of this qualitative study were to explore the information and support provided to dog owners at the time of CCD diagnosis. Additionally, this study aimed to critically evaluate how different sources have impacted owners, how they support their dogs, and make recommendations to improve the support offered.

**Materials and methods:** Sixty-six owners of dogs with CCD completed an online survey, gaining dog and owner demographic information, exploring participant's experience of CCD, support, and information options at and following diagnosis. Data were transcribed and analyzed using descriptive statistics and inductive grounded theory. Semi-structured interviews were completed with six participants, focusing on the owner’s experiences of diagnosis, and the effectiveness of strategies for providing information and support. Results were transcribed and analyzed using thematic analysis.

**Results:** Owners report many stressors when caring for a dog with CCD. While 98.48% (n = 65) of respondents reported feeling that improving their understanding of CCD had been beneficial in supporting their dog, it was often a challenging and frustrating experience to gain accurate information and consistent support. The interviews identified six key themes: impacts of CCD, the knowledge of diagnosis, experience following diagnosis, seeking answers, approach matters, and awareness and options (Fig. 1). A multimodal approach is required to support owners of dogs with CCD effectively; key areas include symptom awareness and planning for a decline in health.

**Figure 1 (abstract PO. 06) Figj:**
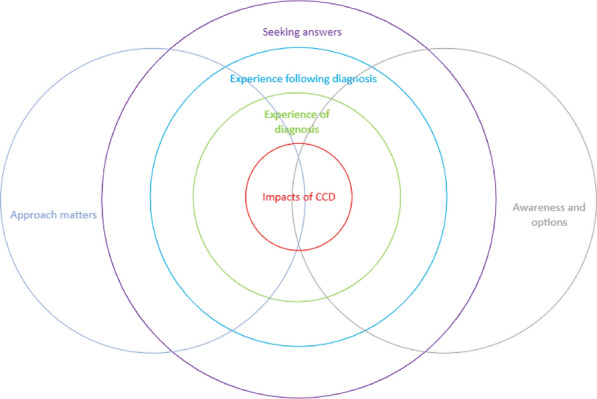
Schematic diagram of interlinking themes

**Conclusion:** Practical support and information are essential in helping owners care for dogs with CCD, including managing symptoms that can lead to caregiver strain and associated health implications for the owner. This research highlighted that owners struggle to get accurate information and support; however, when appropriate and easily accessible, support and information can be effective at helping them to navigate the stressors of CCD. Providing accessible and focused ‘multimodal’ resources is vital to ensuring the well-being of both the owner and the dog with CCD.


**References**
Adams B, Chan A, Callahan H, Milgram NW. The canine as a model of human cognitive aging: recent developments. Prog Neuropsychopharmacology Biol Psychiatry. 2000; 24:675–92.Fast R, Schütt T, Toft N, Møller A, Berendt M. An observational study with long-term follow-up of canine cognitive dysfunction: clinical characteristics, survival, and risk factors. J Vet Intern Med. 2013.


## PO. 07 The use of laser class IIIB as an aid in injury healing

### **Letícia Nosdeo**^1^, Fernanda Vituri^1^, Daniela Loureiro Henrique^2^

#### ^1^Instituto Amarelo Vet, São Paulo, São Paulo, Brazil; ^2^Universidade Federal de Lavras, Minas Gerais, Brazil

##### **Correspondence:** Letícia Nosdeo (nosdeovet@gmail.com)

*Acta Veterinaria Scandinavica* 2023, **65(Suppl 1)**:PO. 07

**Background:** Photobiomodulation is a therapy of choice for injury treatment because it can considerably stimulate the formation of new capillaries, leading to better tissue oxygenation, which in turn accelerates the healing process [1, 2]. The three phases present in the process of tissue repair suffer positive influence after using a laser. Besides the therapy having a powerful analgesic effect, several studies indicate an essential role of photobiomodulation in reducing inflammation and edema [3, 4].

**Materials and methods:** Male canine patient, Labrador Retriever, 10 years old, was seen with a previous history of excision of a nodule with unknown characteristics without histopathological examination, in the right pelvic limb region, without enough surgical margin for suture, thus occurring healing by second intention. The wound that was formed extended throughout the medial region of the thigh, causing the patient a lot of discomforts. Photobiomodulation was used as an auxiliary technique to accelerate the healing process and bring greater quality of life and comfort to the patient. The therapy began with the application of a therapeutic laser, through the DMC—Theravet® device, using the red laser, with a wavelength of 660 nm, at a dose of 4J per point, at equidistant points every 2 cm, along the entire length of the lesion, with intervals between applications of 2 to 3 days, until complete closure.

**Results:** After 22 applications of the therapeutic laser, within 3 months, complete closure of the wound was observed.

**Figure 1 (abstract PO. 07) Figk:**
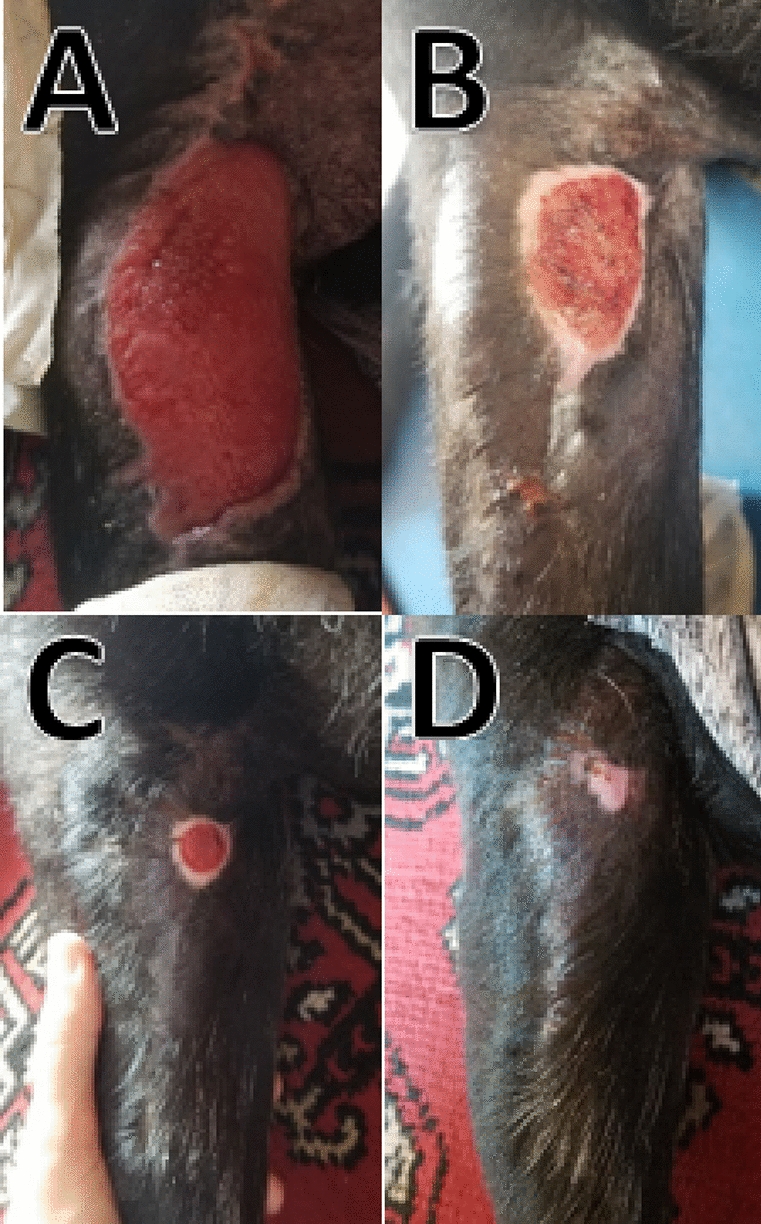
Evolution of the wound after the laser applications. Being A on day 0, B after 8 applications, C after 17 applications, and D after 22 applications

**Conclusion:** The use of laser therapy is a highly effective tool when used to assist in cases of open wound healing. Due to its biological effects, such as granulation stimulation, epithelialization, and other cell growth factors, this technique accelerates the healing time in relation to the average time when there is no associated technique.


**References**
Feng J et al. Low-power laser irradiation (LPLI) promotes VEGF expression and vascular endothelial cell proliferation through the activation of ERK/Sp1 pathway. Cell Signal. 2012; 24:1116–25.Schindl A et al. Systemic effects of low-intensity laser irradiation on skin microcirculation in patients with diabetic microangiopathy. Microvasc Res. 2002; 64:240–6.Enwemeka CS et al. The efficacy of low-power lasers in tissue repair and pain control: a meta-analysis study. Photomed Laser Surg, 2004; 4: 323–9.Pryor B, Millis DL. Therapeutic laser in veterinary medicine. Vet Clin North Am Small Anim Pract. 2015; 45: 45–56.


## PO. 09 Rehabilitation and physical therapy's role in a multidisciplinary team in veterinary palliative care service

### **Fernanda Vituri**^1^, Giselle Ayres Razera Rossa^1^, Vinícius Perez^2^, Bruna Bianchini^2^

#### ^1^Instituto Amarelo Vet, São Paulo, Brazil, ^2^Instituto Kairós, São Paulo, Brazil

##### **Correspondence:** Fernanda Vituri (fervit@gmail.com)

*Acta Veterinaria Scandinavica* 2023, **65(Suppl 1)**:PO. 09

**Background:** Palliative care is a clinical approach that aims to take care of patients with life-threatening illnesses, through appropriate symptom control, rehabilitation, and family support. This kind of care should be present from the disease diagnostic simultaneously to the curative treatment, and its relevance increases along with disease progression and the proximity of the final period of life. To provide this care in a global and holistic way, the multi-professional approach with an interdisciplinary focus is necessary to build the patient and family care plan. Rehabilitation contributes to maintaining patient functionality and autonomy, which often declines with the disease progress and directly affects the disease experience. In this context, physical therapy promotes improving mobility, blood flow, and motor coordination, treating functional limitations, accelerating healing, and reducing pain through physical and manual treatments. Besides its crucial role in controlling symptoms such as dyspnea, fatigue, and anorexia-cachexia syndrome beyond other frequent symptoms in cancer patients.

**Methods and methods:** We developed a Veterinary Palliative Care Service, with simultaneous sessions with physical therapists, acupuncturists, and palliative veterinary veterinarians, with the goal of increasing offerings to animals with severe illnesses and injuries, such as cancer, advanced arthritis, and mobility concerns, kidney disease, heart disease beyond others. Beyond clinical treatment and controlling symptoms, our service is also focused on supporting caretakers in their experiences through the disease process of their pets.

**Results:** In our experience with multidisciplinary veterinary palliative care service, since April 2019, patients experienced less frequent and intense symptoms, less frequent and shorter hospital internment, and longer lifetime, compared with patients we treat in conventional services and compared to literature reports.

**Conclusion:** Even though more objective measures, such as the quality of life, mobility, and pain scales, must be implemented to improve the assessment of measurable results achieved through multi-disciplinary veterinary palliative care service, our experience and caretaker’s feedback strongly encourage us to expand our service and spread our expertise, contributing to provide softer experiences to caretakers and pets in their end-of-life period.

## PO. 10 Caregiving burden and the role of veterinary physiotherapy in the management of canine non-surgical neurological disease

### **Lucy Dawson**^1^, Melanie Haine^1^, Louise Corah^1^, Kate Cobb^1^

#### ^1^School of Veterinary Medicine and Science, University of Nottingham, Nottingham, UK

##### **Correspondence:** Lucy Dawson (lucy.dawson123@hotmail.com)

*Acta Veterinaria Scandinavica* 2023, **65(Suppl 1)**:PO. 10

**Background:** It was recently suggested that individuals caring for sick companion animals experience similar stressors as those caring for a friend or relative – a phenomenon known as ‘caregiving burden’ [1]. Assistance for these individuals does not currently exist in veterinary medicine in the same way as in human medicine. Stress for the owner, alongside the reduced quality of life for their pet, increases the consideration of end-of-life decisions, with neurological disease cited as a leading cause of euthanasia in dogs over 3 years of age [2]. Veterinary physiotherapy has the potential to offer the required support for owners.

**Materials and methods:** This study used qualitative methods to examine the impact on owners of caring for a dog with a non-surgical neurological condition, and how Veterinary Physiotherapy influenced their experience. Owners of dogs with relevant medical conditions were invited via social media groups and email to complete an online survey. Respondents were further invited to partake in semi-structured interviews to explore their experiences in more depth. Interviews were transcribed and thematic analysis was performed to identify important themes.

**Results:** 53 UK dog owners completed the survey. Data showed owners attending more appointments with a Veterinary Physiotherapist were significantly more likely to agree it helped their pet. Interactions with vets played a key role in owners’ decisions to attend physiotherapy with their pets. Two owners were interviewed following survey completion. Three themes were developed; the physical, mental, and emotional challenges to caring for a disabled dog, how owners often relied upon their own research and social media support groups, and the positive experiences of utilizing veterinary physiotherapists.

**Conclusions:** This study supports previous research that the caregiving burden exists for owners of sick companion animals. Owners benefit from seeking support from those with shared experience and knowledgeable professionals. Reputable online resources to direct owners to are vital. Veterinary Physiotherapists should be incorporated into a multidisciplinary veterinary team when managing non-surgical neurological diseases in dogs and veterinary surgeons should support owners as well as their canine patients when managing these cases.


**References**
Spitznage MB, Jacobson DM, Cox MD, Carlson MD. Caregiver burden in owners of a sick companion animal: a cross-sectional observational study. Veterinary Record. 2017; 181(12).O’Neill DG, Church DB, McGreevy PD, Thomson PC, Brodbelt DC. Longevity and mortality of owned dogs in England. Vet J. 2013; 198: 638–43.


## PO. 11 Audioarthrology – a potentially useful tool for assessing joint disease

### **Darryl Millis**^1^, Molly Werder^1^, Elizabeth Sutherland^1^, Sang Woo^1^, Cassio Ferrigno^1^, Marti Drum^1^

#### ^1^Department of Small Animal Clinical Sciences, University of Tennessee, Knoxville, Tennessee, USA

##### **Correspondence:** Darryl Millis (dmillis@utk.edu)

*Acta Veterinaria Scandinavica* 2023, **65(Suppl 1)**:PO. 11

**Background:** Recent studies in human medicine have evaluated sounds produced by the knee and the temporomandibular joints, but similar technology has yet to be evaluated in veterinary medicine [1, 2]. The use of sound heard through a stethoscope placed on joints has the potential to be useful as a diagnostic tool for veterinarians. The application of a stethoscope to characterize joint pathology may decrease the cost of diagnostics for joint injury and disease and may be a minimally invasive tool for the diagnosis and monitoring of joint disease. It is possible that distinct audible sounds such as popping, clicking, or grinding may be auscultated in joints and aid in assessment. We report cases of audioarthrology evaluation of hip joint pathology.

**Materials and methods:** A digital stethoscope (3 M™ Littmann® CORE stethoscope, Eko Device Inc, Oakland, CA) with Bluetooth technology was used to record sounds from two hip joints while performing an Ortolani sign. One dog had a palpable Ortolani sign with crisp reduction and no radiographic evidence of osteoarthritis. A second dog was similarly evaluated, but this dog had the sensation of crepitus during joint reduction and had radiographic evidence of osteoarthritis. Digital recordings were evaluated for possible differences.

**Results:** Graphical representations of both recordings were evaluated. The dog with crepitus had a wider and higher amplitude graphical representation of joint sound compared to the dog without osteoarthritis, especially during reduction (Figs. 1 and 2).

**Figure 1 (abstract PO. 11) Figl:**
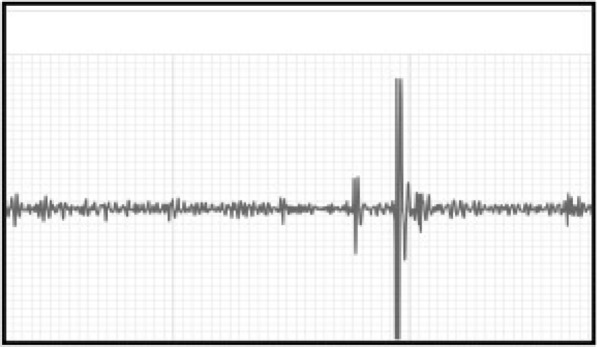
Acoustic recording from a dog with an Ortolani sign and no radiographic osteoarthritis. The smaller first peak represents subluxation of the hip, and the second larger peak represents reduction of the joint

**Figure 2 (abstract PO. 11) Figm:**
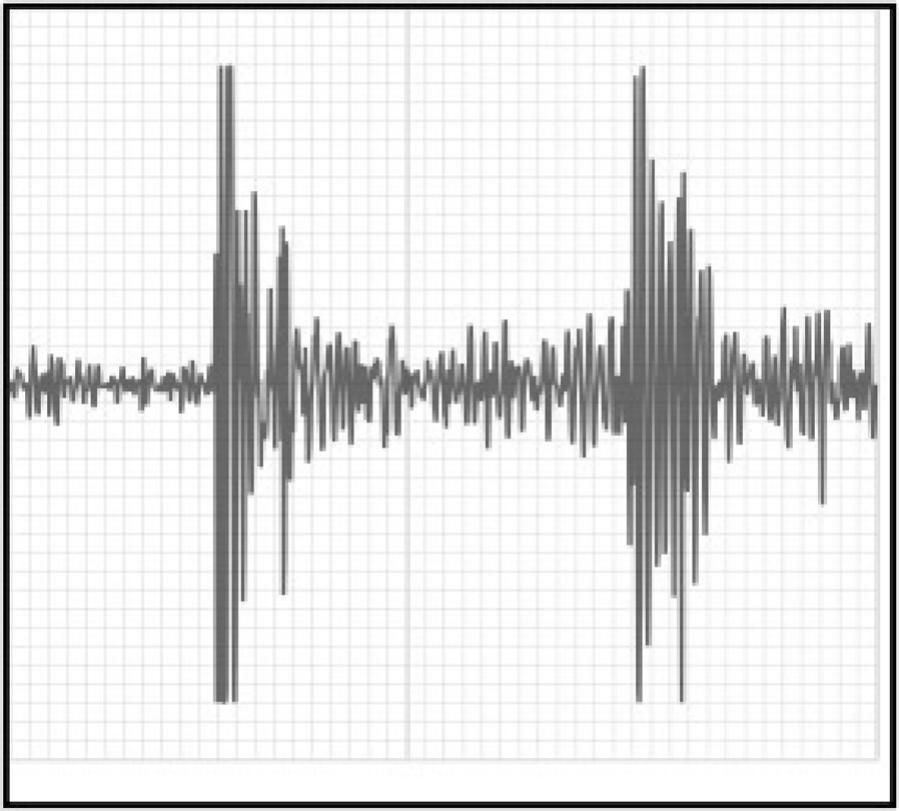
Acoustic recording from a dog with an Ortolani sign and radiographic osteoarthritis. The first peak represents subluxation of the hip, and the second larger peak represents reduction of the joint. Notice the larger and wider acoustic deflection of the first peak associated with subluxation, and the wider deflection of the second peak associated with joint reduction as compared to the dog without hip osteoarthritis (see Fig. 1)

**Conclusion:** Based on these very preliminary results, audioarthrology may be a useful tool to characterize joint disease in a noninvasive, cost-effective manner. A clinical study of 50 canine joints is currently in progress to further characterize clinical applications of audioarthology. Normal and diseased elbow, stifle, and hip joints will be evaluated for acoustic waveforms.


**References**
Cheng YT, Tai CC, Chou W, Tang ST, Lin JH. Analyzing the audio signals of degenerative arthritis with an electronic stethoscope. Rev Sci Instrum. 2018;89:085111. https://doi.org/10.1063/1.5018006Dagar SR, Turakiya V, Pakhan AJ, Jaggi N, Kalra A, Vaidya V. Modified stethoscope for auscultation of temporomandibular joint sounds. J Int Oral Health. 2014;6:40–4.


## PO. 12 Physiotherapy protocol applied in case of quadriceps muscle contracture without previous surgical treatment

### **Daniela Loureiro Henrique**^1^, Beatriz Ribeiro Gaspar2, Júllia de Almeida Lima^3^, Fernanda Vituri^4^, Daniel Chechinatto^1^, Luis David Solis Murgas^5^

#### ^1^PhD student in Animal Physiology and Metabolism at Federal University of Lavras; ^2^Student at the University of São Paulo (USP). São Paulo-SP Brazil; ^3^Student at the Federal University of Lavras (UFLA) Lavras- MG Brazil; ^4^Veterinary Physician with specialization in Physiotherapy and Veterinary Orthopedics; ^5^Titular Professor Department of Veterinary Medicine at the Federal University of Lavras (UFLA) Lavras- MG Brazil

##### **Correspondence:** Daniela Loureiro Henrique (daniela@pataepatela.com.br)

*Acta Veterinaria Scandinavica* 2023, **65(Suppl 1)**:PO. 12

**Background:** Orthopedic conditions in dogs and cats include fractures of long bones which commonly require surgical repair for optimal outcomes [1, 2]. However, [1] depending on the patient's size, age, type of activity, fractured bone, and type of fracture, the orthopedist may opt for conservative treatment such as splints or bandages. With immobilization, disuse of the limb, muscle atrophy, joint blockage, and contraction of muscles, tendons, and ligaments are common [3], when joint changes are severe enough that they prevent the dog from functioning, arthrodesis and limb amputation are recommended [3, 4]. In these cases, the use of physiotherapy can be tried before surgery as it provides pain relief, muscle relaxation, reduces the inflammatory process, helps with bone healing, and improves joint movement, among other benefits [5, 6]. Quadriceps contractures are one of these conditions and are most frequently seen in puppies with distal femoral fractures [3, 4].

**Materials and methods:** A six month old female mixed breed dog presented with a lesion between the digits of the right foot secondary to a quadriceps contracture and dragging the foot. This was due to the utilization of an immobilizing splint for two months due to a fracture of the tibia (stifle was immobilized). The diagnosis of muscle contracture was based on the clinical examination (atrophy [circumference of the right thigh was 31 cm while the left thigh was 38 cm]), goniometry was 50° flexion on the left knee, 170° extension, and left tarsus: 60° flexion, 140° extension. In the injured limb, the knee flexion angle was 150°, extension, 180°; in the tarsus was: flexion 153°, extension 180° (Fig. 1). Examining the two limbs together using a counter-extension, a height difference of 8 cm was found. At the beginning of treatment, massage therapy techniques, pulsed electromagnetic field therapy (PEMF) to promote analgesia and muscle relaxation, joint mobilization were used to increase joint amplitude, in this case, decrease the flexion of the right knee and tarsus. Therapeutic exercise and hydrotherapy (Fig. 2) were included started at the 10th session.

**Results:** After 60 physiotherapy sessions (Fig. 3), the goniometry of the joints was stifle: 85° flexion (43%) tarsus flexion 90° (33.5%), and the difference in circumference was 3.0 cm between the limb healthy and the injured limb (Table 1).

**Figure 1 (abstract PO. 12) Fign:**
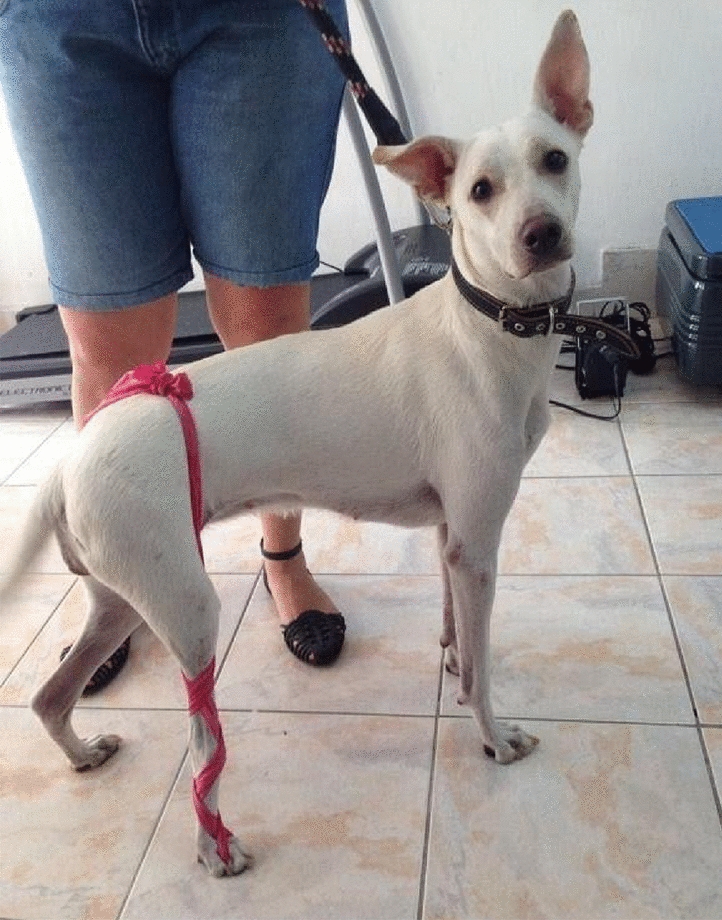
Patient at beginning of physiotherapy treatment

**Figure 2 (abstract PO. 12) Figo:**
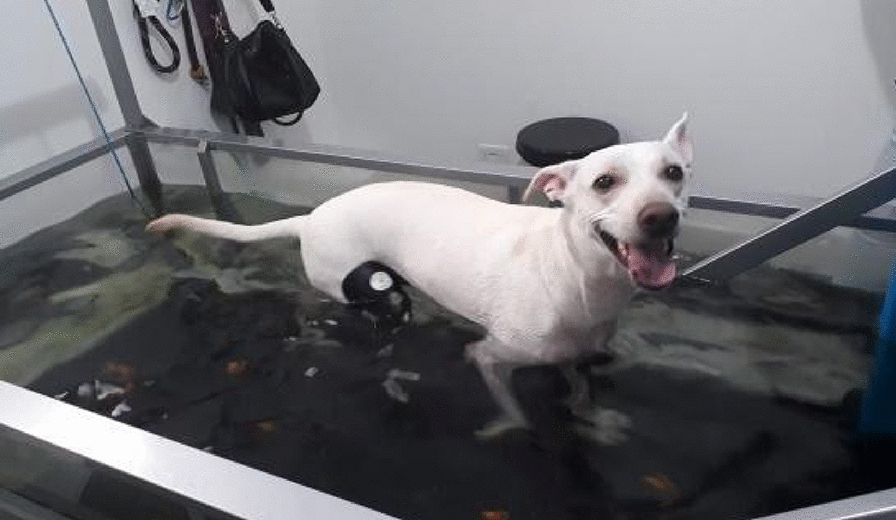
Patient performing hydrotherapy

**Figure 3 (abstract PO. 12) Figp:**
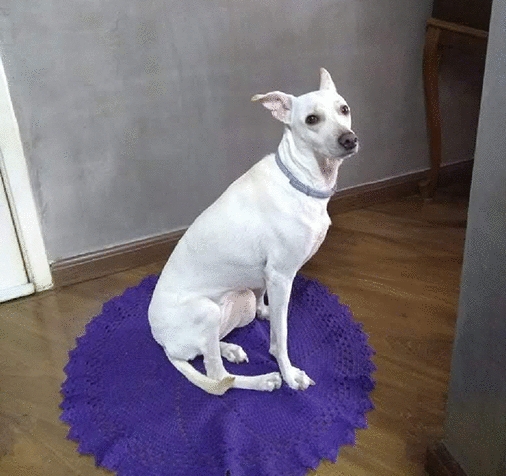
Patient after 60 sessions

**Table 1 (abstract PO. 12) Tabd:** Comparison of the values obtained in flexion of the right stifle and tarsus joints

Goniometry	Initial Assessment	After 1 month:	After 1 year:
FTP flexion	150º	140º	85º
TT flexion	153º	149º	90º

**Conclusion:** Physiotherapy was fundamental for a better return to limb functionality and quality of life for the patient.


**References**
Millis DL. Quadriceps Contracture In: Griffon D, Hamaide A: Complication in small animal surgery;2016; p.692–6.Johnson AL 2007 Tratamento de fraturas específicas in: Fossum, T. W. Cirurgia de pequenos animais. Mosby Elsevier, 4 ed., 2007, 3122–435.Taylor J, Tangner CH, Acquired muscle contractures in the dog and cat: a review of the literature and case report; Vet Comp Orthop Traumatol; 2007; 20:79–85.Ulusan S, Captug-Ozdemir O, Sul-Sancak I, Bilgili H. Treatment techniques of femoral quadriceps muscle contracture in ten dogs and two cats. Kafkas Univ Vet Fak Derg; 2011; 17: 401–408.Manning AM. Physical rehabilitation for the critically injured veterinary patient. In Millis DL, Levine D, Taylor RA. Canine rehabilitation & physical therapy. Saunders; 2004; p.404–10.Flaherty MJ. Rehabilitation therapy in perioperative pain management. In Barr, C, Gianotti, G.; Alternative to opioid analgesia in small animal anesthesia; Elsevier 2019; p.1143–59.


## PO. 13 Extracorporeal focused shock wave treatment for nonunion fracture of the tibia in a dog

### **Kirsten Häusler**^1^

#### ^1^Tierphysiotherapie Team Häusler, Stuttgart, Deutschland

##### **Correspondence:** Kirsten Hausler (info@dr-haeusler.com)

*Acta Veterinaria Scandinavica* 2023, **65(Suppl 1)**:PO. 13

**Background:** Extracorporeal shock wave treatment (ESWT) is a non-invasive treatment that has been shown to stimulate bone regeneration. Bone fractures classified as non-union are challenging in humans as in animals. They carry a high risk of infection and failure of osteosynthesis. This case report shows an alternative treatment strategy for the veterinary field.

**Materials and methods:** A small mixed-breed dog was presented with off-loading the right hind leg. X-rays were taken and showed a non-union of the tibia. The dog’s gait was initially evaluated on the instrumented treadmill to get a baseline before being treated with extracorporeal shockwave (focused) for three treatments weekly. Follow-up x-rays were taken four weeks after the last shockwave treatment. Gait analysis was performed before, after the third, final treatment, and four weeks after the previous treatment. The x-rays showed substantial bony changes after three treatments, the fracture gap had closed entirely.

**Results:** Extracorporeal shock wave treatment proved to help heal this non-union of the tibia within seven weeks. It is a non-invasive treatment, and it does not require anesthesia and, therefore, should be considered a conservative option to treat non-unions in dogs.

**Conclusion:** These findings need to be followed by clinical studies to fully understand the biological mechanisms provoked by using extracorporeal-focused shock waves.

## PO. 14 Long-term follow-up in osteoarthritis dogs after a multimodal functional rehabilitation protocol with intra-articular allogenic stem cells

### **Débora Gouveia**^1^, Inês Roque do Vale^2^, Ana Cardoso^2^, Carla Carvalho^1^, Oscár Gamboa^4^, António Ferreira^4,5^, Rute Canejo-Teixeira^3^, Ângela Martins^2,3^

#### ^1^Arrábida Veterinary Hospital—Arrábida Animal Rehabilitation Center, 2925-538, Azeitão, Setúbal, Portugal; ^2^Superior School of Health, Protection and Animal Welfare, Polytechnic Institute of Lusophony, Campo Grande, 1950-396 Lisboa, Portugal; ^3^Faculty of Veterinary Medicine, Lusófona University, Campo Grande 376, 1749-024 Lisboa, Portugal; ^4^Faculty of Veterinary Medicine, University of Lisbon, 1300–477 Lisboa, Portugal; ^5^CIISA—Centro Interdisciplinar-Investigação em Saúde Animal, Faculdade de Medicina Veterinária, Av. Universidade Técnica de Lisboa, 1300-477 Lisboa, Portugal

##### **Correspondence:** Débora Gouveia (vetarrabida.lda@gmail.com)

*Acta Veterinaria Scandinavica* 2023, **65(Suppl 1)**:PO. 14

**Background:** Osteoarthritis is a degenerative osteoarticular disease with long-term progression. Multimodal protocols in daily practice are essential for extending life expectancy and promoting well-being in geriatric dogs. The present preliminary prospective cohort study intends to prove if the association of functional rehabilitation multimodal protocol with intra-articular administration of stem cells [1] is tolerable, safe, repeatable, and effective.

**Materials and methods:** There were selected 9 dogs with age 2 years old, weight 15 kg, severe chronic pain 3, lameness score of 5, radiological exam classified as grade 3, and osteoporosis 2. All dogs, on day 1 (T0), underwent an intra-articular administration of allogenic stem cells from the synovial membrane, each dose with 5 × 10^6^ cells (98% viability and after microbiological evaluation) (Fig. 1), followed by shock-waves (1000 pulses per joint, energy E5/E6) (Fig. 2) and laser therapy class IV (940 nm, 5 days in a row), performed on the coxofemoral and femoro-tibia-patellar joints. Re-evaluations were implemented on T1 (7 days), T2 (14 days), F1 (30 days), F2 (90 days), F3 (180 days), F4 (270 days), F5 (365 days), and F6 (480 days), always performing the pain score, lameness score, range of motion (ROM) measure with goniometry and muscle mass measure with Gulick II tape.

**Results:** In 100% of the population, it was shown reduction from severe pain (T0) to mild pain (F6). Also, it was demonstrated recovery of muscle mass, starting in T1, and improvement of flexion/extension ROM of both joints, always with progressive evolution, similarly to other authors [2–4]. Follow-up consultations over time were key in demonstrating the continuity of this improvement [5–7]. Comparing the present study with Cuervo et al. [8] by the one-sample t-test, it was shown a strong significance (t(7) = 2.518, P = 0.04), allowing comparison. Both studies had positive outcomes, although in the same study the long-term follow-ups were until 6 months, while in our study 4 dogs achieved 16 months.

**Conclusions:** The protocol has fulfilled all the mentioned objectives and showed to be effective in dogs with osteoarthritis, with records of long-term follow-ups. It would be interesting to continue this study, achieving a higher number of dogs.

**Figure 1 (abstract PO. 14) Figq:**
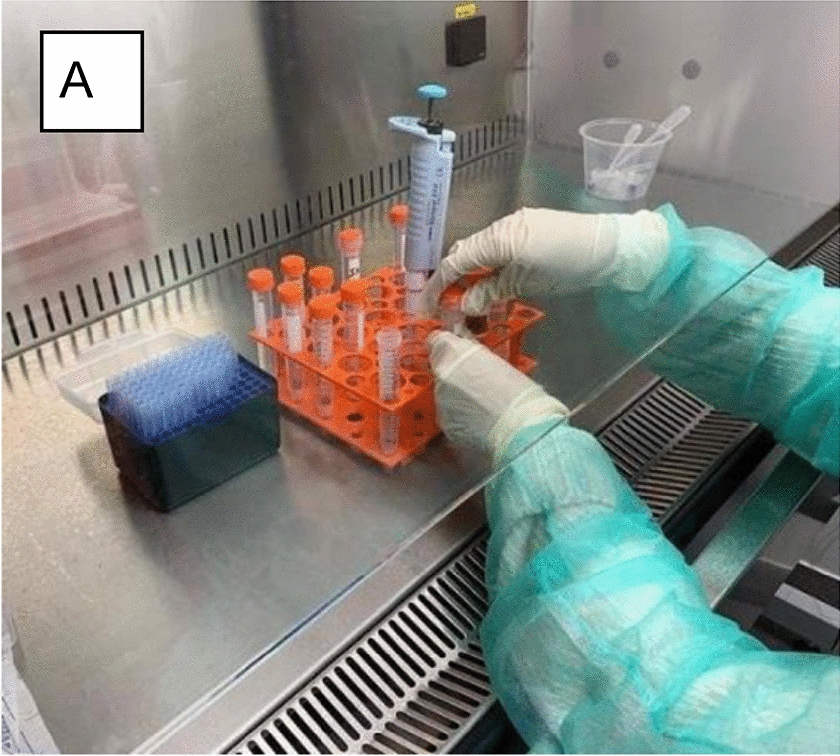

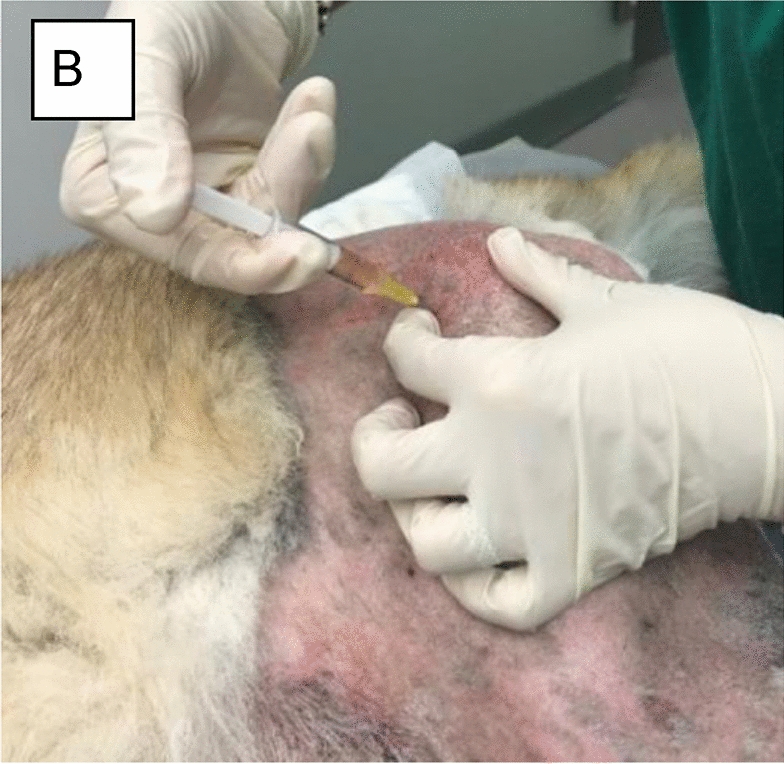
Regenerative Medicine with stem cells. A: Stem cell preparation; B: Stem cell administration

**Figure 2 (abstract PO. 14) Figs:**
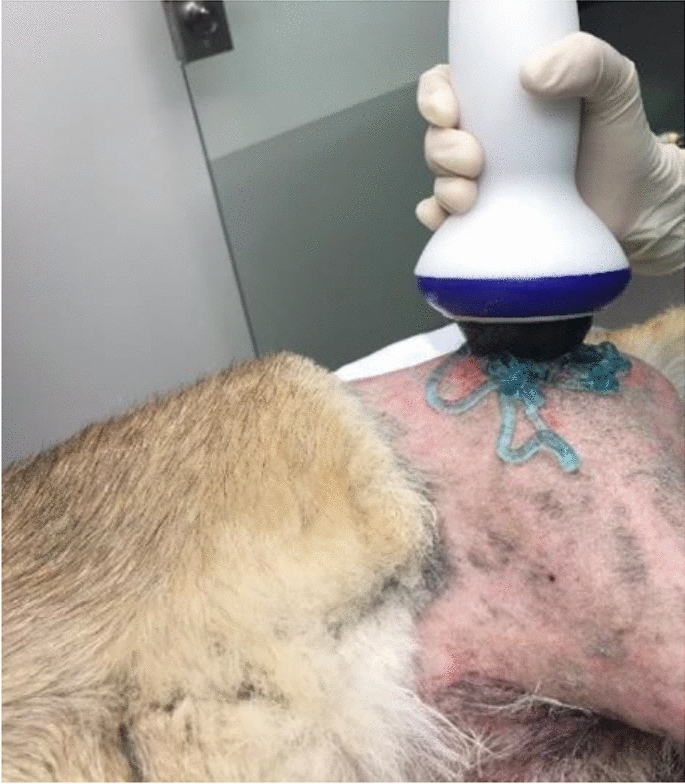
Shock waves administration in the coxofemoral joint of a dog


**References**
Orozco L, Munar A, Soler R, Alberca M, Soler F, Huguet M, et. al. Treatment of knee osteoarthritis with autologous mesenchymal stem cells. Transplantation. 2013. 95: 1535–41.Zellner J, Pattappa G, Koch M, Lang S, Weber J, Pfeifer CG, et. al. Autologous mesenchymal stem cells or meniscal cells: what is the best cell source for regenerative meniscus treatment in an early osteoarthritis situation? Stem Cell Res Ther. 2017. 8: 225.Kondo S, Muneta T, Nakagawa Y, Koga H, Watanabe T, Tsuji K, et. al. Transplantation of autologous synovial mesenchymal stem cells promotes meniscus regeneration in aged primates. J Orthop Res. 2017. 35: 1274–82.Kim SE, Pozzi A, Yeh JC, Lopez-Velazquez M, Yong JAU, Townsend S, et. al. Intra-articular umbilical cord-derived mesenchymal stem cell therapy for chronic elbow osteoarthritis in dogs: a double-blinded, placebo-controlled clinical trial. Front Vet Med. 2019. 6:474.Matas L, Orrego M, Amenabar D, Infante C, Tapia-Limonchi R, Cadiz MI, et. al. Umbilical cord-derived mesenchymal stromal cells (MSCs) for knee osteoarthritis: repeated MSC dosing is superior to a single MSC dose and to hyaluronic acid in a controlled randomized phase I/II trial. Stem Cells Transl Med. 2019. 8:215–24.Lee W, Kim HJ, Kim K, Kim GB, Jin W. Intra-articular injection of autologous adipose tissue-derived mesenchymal stem cells for the treatment of knee osteoarthritis: a phase IIb, randomized, placebo-controlled clinical trial. Stem Cells Transl Med. 2019. 8: 504–11.Cuervo B, Rubio M, Sopena J, Dominguez J, Vilar J, Morales M, et. al. Hip osteoarthritis in dogs: a randomized study using mesenchymal stem cells from adipose tissue and plasma rich in growth factors. Int J Mol Sci. 15: 13,437–60.


## PO. 15 Rehabilitation of a pygmy goat with hindlimb paralysis

### **David Levine**^1^, Sean Byrd^2^

#### ^1^University of Tennessee at Chattanooga and Veterinary Care and Specialty Group, Chattanooga, United States; ^2^Skyview Animal Clinic, Cape Girardeau, United States

##### **Correspondence:** David Levine (David-Levine@utc.edu)

*Acta Veterinaria Scandinavica* 2023, **65(Suppl 1)**:PO. 15

**Background:** American pygmy goats (*Capra aegagrus hircus*) are a breed of achondroplastic goats that are small and stocky with an adult weight of 23–39 kg [1]. As they are good-natured and trainable, they are found as companion animals and are common in petting zoos in the United States. There is a paucity of literature on formal rehabilitation in pygmy goats.

**Materials and methods:** The patient was a 6-month-old, male, neutered pygmy goat (11.4 kg) that was bitten by a mule resulting in a thoracolumbar fracture at T11 (Fig. 1) with gas opacity seen and draining tract lesions bilaterally at T11-L1 with resultant bilateral paraplegia, which has also been documented in pygmy goats resulting from lymphosarcoma [2]. Interventions included flunixin meglumine (Banamine) 0.2 ml SID, enrofloxacin (Baytril) 100 mg/mL, 1.0 ml sid, and dexamethasone 0.7 mL SID, all for 3 days at time of injury. The patient was a household pet.

**Results:** At initial rehabilitation evaluation, passive range of motion (ROM) using the normal convention for dogs [3] is reported in Table 1. The front limbs were normal. Neurological examination found an absence of conscious proprioception, motor function, and superficial and deep pain in the hindlimbs bilaterally. Panniculus reflex was absent from the T12 distal. Rear limbs were stiff when the range of motion test (Table 1) was performed, and contractures were developing in the hips, stifles, and tarsal joints. Cranial nerve examination was WNL. By discharge, the wounds were healed, the patient gained significant rear limb range of motion (Table 2), had no discomfort in any limb and was able to ambulate independently.

**Figure 1 (abstract PO. 15) Figt:**
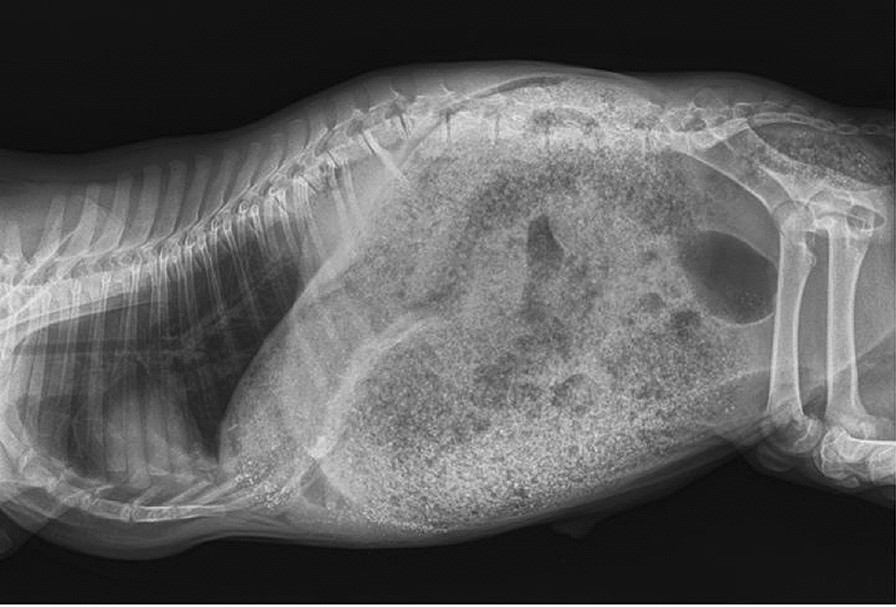
Fracture at T11 with draining tracts

**Table 1 (abstract PO. 15) Tabe:** Passive range of motion at initial evaluation

	L Flex	L Ext	R Flex	R Ext	L Total ROM	R Total ROM
Hip	30°	67°	50°	75°	37°	25°
Stifle	15°	90°	20°	85°	75°	65°
Tarsus	90°	150°	70°	140°	60°	70°

Left = L, Right = R, Flex = Flexion, Ext = Extension

**Table 2 (abstract PO. 15) Tabf:** Passive range of motion at discharge (3 months after initial evaluation)

	L Flex	L Ext	R Flex	R Ext	L Total ROM	R Total ROM
Hip	30°	140°	25°	105°	110°	80°
Stifle	15°	100°	15°	110°	85°	95°
Tarsus	30°	152°	25°	140°	122°	115°

Left = L, Right = R, Flex = Flexion, Ext = Extension

**Figure 2 (abstract PO. 15) Figu:**
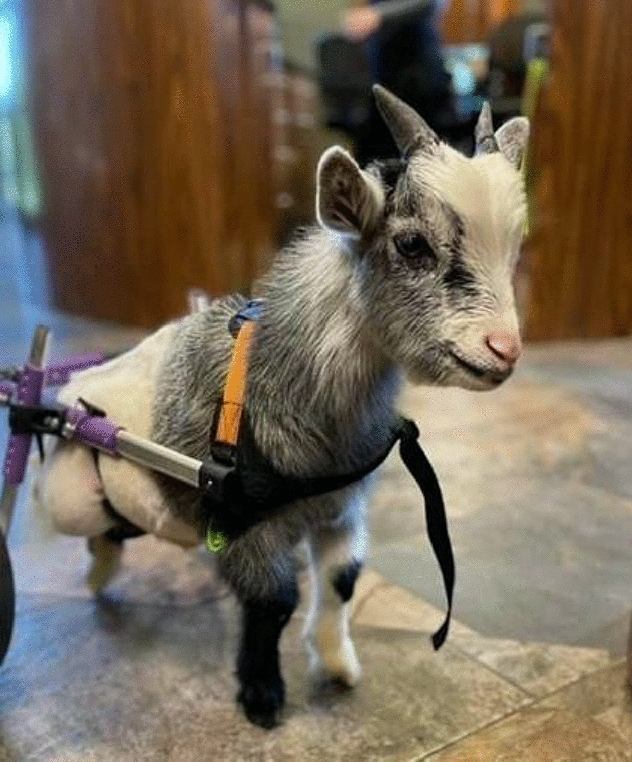
Patient in wheelchair

**Conclusion**: This case discusses adaptations in rehabilitation within the clinic and home exercises utilized. Carprofen (1.0 mg/kg po) once daily was used to decrease pain for a 3-week period at the onset of rehabilitation. Modalities included on-contact Class 4 laser (Companion Animal Health) for the wounds over the spine at the injury site (4 J/cm^2^). Exercises included thermotherapy for 15 min prior to passive ROM and stretching in the hindlimbs, assisted standing over peanuts, and cart fitting (Fig. 2). The cart allowed for independent ambulation with the rear limbs held in stirrups. As pygmy goats are not fully grown until approximately 3 years of age, readjustments and refitting of the cart will continue. The primary modification of the cart was lowering the leg rings to allow the patient to be in more a sitting position, widening the cart to allow for the width of the barrel of the patient, and padding to prevent skin breakdown.


**References**
American pygmy goat. AZ Animals. https://a-z-animals.com/animals/american-pygmy-goat/. Published July 12, 2021. Accessed June 11, 2022.Gygi M, Kathmann I, Konar M, Rottenberg S, Meylan M. Paraparese bei einer zwergziege: abklärung mittels magnetresonanztomographie [Paraparesis in a dwarf goat: clarification by means of magnetic resonance imaging]. Schweiz Arch Tierheilkd. 2004;146:523–8. doi:10.1024Jaegger G, Marcellin-Little DJ, Levine D. Reliability of goniometry in Labrador Retrievers. Am J Vet Res. 2002;63:979–86; doi:10.2460


## PO. 16 Evaluation of unusual equine hindlimb lameness in the field

### **Helen Robartes**^1^, Michael Fitzgerald^2^

#### ^1^Helen The Physio, New Zealand; ^2^Vet Services Hawke's Bay, New Zealand

##### **Correspondence:** Helen Robartes (helen@vetphysio.co.nz)

*Acta Veterinaria Scandinavica* 2023, **65(Suppl 1)**:PO. 16

**Background:** Peroneus Tertius rupture in the horse is an uncommon diagnosis. The appearance of the lameness and the tests associated with this injury are pathognomic [1, 2], but maybe missed by clinicians who have not seen this type of case before.

**Materials and methods:** This case report focuses on the multidisciplinary management of a horse diagnosed with peroneus tertius rupture by an equine veterinarian and physiotherapist working together to evaluate an unusual lameness observed in the field, and retrospective considerations on how to manage such a case in the future. Video and photographic footage are available to supplement the verbal description of the lameness and rehabilitation. The veterinarian and physiotherapist used published resources as well as advice from experienced colleagues to cooperate during the horse’s management and provide the best guidance to the owner.

**Results:** Video and photographic footage are available to supplement the verbal description of the lameness and rehabilitation. The veterinarian and physiotherapist used published resources as well as advice from experienced colleagues to cooperate during the horse’s management and provide the best guidance to the owner.

**Conclusion:** Collaboration between an equine veterinarian and a physiotherapist is useful for the management of horses, especially for the evaluation of unusual lameness in the field. These clinicians’ different approaches to assessment and rehabilitation, underpinned by published evidence and with expert input, can provide the owner with the best management advice.


**Acknowledgments**


Thanks to the owner of the horse in this case report for allowing this footage and information to be shared.


**Consent to publish**


Written informed consent to publish has been obtained by the owner of the horse in this case report.


**References**
Ross M, Dyson S. Lameness, other soft tissue injuries; in The horse. 2nd ed. Missouri: Saunders Elsevier; 2011, p. 802–3;Koenig J, Cruz A, Genovese R, Fretz P, Trostle, S. Rupture of the peroneus tertius tendon in 25 horses. In AAEP Proceedings [Internet]. 2002 48:326–328. Available from: https://www.ivis.org/sites/default/files/library/aaep/2002/910102000326.PDF


## PO. 17 Preliminary investigation into the effects of chiropractic treatment, myofascial release, and combined treatment on mechanical nociceptive thresholds of horses

### **Elizabeth Wenman**^1^, Nikki Routledge^2^, Adrian Hunnisett^2^

#### ^1^McTimoney Animal Association/Private Practice, Farnham, Surrey, UK; ^2^McTimoney College of Chiropractic, Abingdon, Oxford, UK

##### **Correspondence:** Elizabeth Wenman (diane.grosjean@ugent.be)

*Acta Veterinaria Scandinavica* 2023, **65(Suppl 1)**:PO. 17

**Background:** Credible evidence supports that chiropractic treatment (CT) of equine spinal misalignments helps restore joint range of motion (ROM) [1] improving soft tissue function whilst diminishing pain [2]. Myofascial release (MFR) acts by reducing myofascial constrictions [3], increasing spinal vertebrae joint ROM [4]. The study aimed to objectively investigate the effect of CT and MFR treatments over time and to ascertain if combining these two therapeutic modalities produced a synergistic effect. The established method of Pressure Algometry (PA) was used to measure the mechanical nociceptor thresholds (MNTs), an indicator of musculoskeletal tenderness [1], of the thoracolumbar musculature of riding school horses.

**Materials and methods:** This repeated measure study used 20 riding school horses with no known back pathologies, aged between 5 and 15 years (mean age 11.25 years ± 2.9 years). Random allocation to four groups (n = 5), control group (no intervention), chiropractic, myofascial release, and combined treatment groups. With Veterinary consent, all treatments were undertaken by a single qualified McTimoney animal practitioner. While the horses stood square, Research Assistant A measured MNTs bilaterally in triplicate, using an FDK-60 pressure algometer on the epaxial muscles located 10 cm ventral to the spine at T9, T13, L3, and L6. The results were recorded by Research Assistant B. Both research assistants were blinded to the treatment groups. The time points used were pre-treatment, post-treatment, Day 1, and Day 7 post-treatment. Data was tested for normality by the Kolmogorov–Smirnov test. Group data was analyzed over time points using the Kruskal–Wallis H test with post hoc tests.

**Results:** The median range of all measuring points for the three consecutive measurements was 1–3 kg/cm^2^. There was no significant difference (P > 0.05) pre-treatment to Day 7 for neither the control nor MFR group. There was a significant increase in mean MNTs pre-treatment to Day 7 for both the CT and combined MFR & CT treatment groups, of 9.25% and 32.34% respectively.

**Conclusion:** This study provides positive evidence that a single CT treatment and combined treatment of MFR and CT, illicit statistically significant reductions in musculoskeletal tenderness. Indicated by increased MNTs of specific equine thoracolumbar musculature, for up to 7 days, when compared to no treatment and MFR groups. Equine chiropractic practitioners may enhance their therapeutic outcome by delivering MFR immediately prior to CT. Further investigation is warranted with larger cohorts and over longer time periods.


**References**
Haussler K, Erb H. Mechanical nociceptive thresholds in the axial skeleton of horses. Equine Vet J Suppl. 2006; 38:70–5.Gómez Álvarez C, L’Ami J, Moffatt D, Back W, van Weeren, P. Effect of chiropractic manipulations on the kinematics of back and limbs in horses with clinically diagnosed back problems. Equine Vet J Suppl. 2008; 40:153–9.Shah S. Bhalara A. Myofascial release. J Health Sci. 2012; 2: 69.Mauntel T. Clark M. Padua D. Effectiveness of myofascial release therapies on physical performance measurements: a systematic review. ATSHC 2014; 6:189–96.


## PO. 18 Low-temperature thermoplastic splints in canine rehabilitation

### **Melanie Bruder**^1^

#### ^1^Phoenix Veterinary Physiotherapy and Rehabilitation, Sarah Smith Cardiology, Etwall, United Kingdom

##### **Correspondence:** Melanie Bruder (melbruder@uwclub.net)

*Acta Veterinaria Scandinavica* 2023, **65(Suppl 1)**:PO. 18

**Background:** This paper will describe and illustrate, how the use of custom-made, low-temperature thermoplastic splints, can form part of the management of dogs with musculoskeletal and neurological diseases. There is a paucity of literature on the use of splinting as a part of the rehabilitation of small animals, and there is a need to disseminate the skills required to make custom-made splints.

**Materials and methods:** Case descriptions will be used to illustrate different splints used in the management of a variety of pathologies. These will include Achilles tendinopathies and ruptures, carpal instability, peripheral nerve lesions, joint contractures, atlantoaxial instability, spinal surgery, and joint hypermobility. Material and design selection will be discussed in relation to specific cases. The manufacturing process of low temperature thermoplastic splints will be explained, as well as how the different properties of the materials give versatility in achieving differing outcomes for different cases. Use of neoprene in conjunction with thermoplastics will be included.

**Results:** The selection of suitable cases will be contrasted with guidance around unsuitable cases, including the advantages and disadvantages of using splints against more traditional forms of cooptation. Wearing regimes and follow-up will be covered.

**Conclusion:** The use of bespoke splints in veterinary rehabilitation is a relatively new area for most practitioners. This paper suggests that there is a significant role in the appropriate use of splints, but that it is necessary to develop specific skills in relation to assessment for, selection, and manufacture. Further research and training opportunities will be required in order to establish splint making as a regular tool in the rehabilitation armory.

## PO. 19 The effects of direct and indirect microcurrent therapy on mechanical nociceptive thresholds in the thoracolumbar region of the ridden horse

### Lucinda Munchhausen, **Sarah Roberts***

#### Animal Health, Behaviour and Welfare Department, Harper Adams University, Edgmond, Shropshire, TF10 8NB, UK

##### **Correspondence:** Sarah Roberts (broberts@harper-adams.ac.uk)

*Acta Veterinaria Scandinavica* 2023, **65(Suppl 1)**:PO. 19

**Background:** Ridden horses have increased muscle soreness, swelling and soft tissue lesions, particularly in the thoracolumbar region [1, 2], leading to reduced mechanical nociceptive thresholds (MNT) [3]. Not only is back pain performance limiting, it also compromises the animal’s welfare [4]. Electrotherapies have been shown to increase MNT values in horses [5], although there is emerging evidence to support the use of microcurrent in small animal and human studies [6–9] there is limited published evidence for use in horses. Pressure algometer (PA) is a reliable quantitative method to measure MNT values in horses with back pain [10–12], although it is known circadian rhythms affect PA readings [13], the effects of time and environmental factors on MNT’s are still relatively unknown. The objective of this study was to evaluate the effects of direct and indirect microcurrent therapy on MNT’s in the healthy ridden horse and test the hypothesis that MCT can improve healing by increasing MNT values.

**Materials and methods:** Baseline PA readings were taken from 10 bilateral soft issue landmarks [3] (Fig. 1) from 16 healthy ridden horses then the same day weekly between 3.30–5.30 pm. Horses were randomly assigned to one of two treatment groups. Treatment group one indirect-ArcEquine microcurrent device, applied to alternate lower hind limbs daily for three hours per day/6 weeks following manufactures guidelines. Treatment group two direct- MT-330 microcurrent device placed on the thoracolumbar region for one hour per day/ 4 weeks. Both devices require wetting of the coat and applying a water based conductive gel to the site of the electrode. The PA was operated by the authors, data recording was collected blind by a second person.

**Results:** One horse was withdrawn from treatment group one due a skin reaction at the site of the electrodes. Sites six and eight had lower mean ± s.d baseline readings. Figure 2 shows MNT values increased over the weeks, however RM-ANOVA showed there was no statistical significance for treatment/time p.22 or between treatments p. 94. One – way ANOVA showed time was significant P < 0.001 with the absolute values of change from baseline to end of intervention shown in Fig. 3. Ambient temperature confounded the results P < 0.001 with a positive correlation, Table 1 shows the interaction of temperature on MNT value per site. Figure 4 shows treatment group two appears to be less influenced by temperature.

**Figure 1 (abstract PO. 19) Figv:**
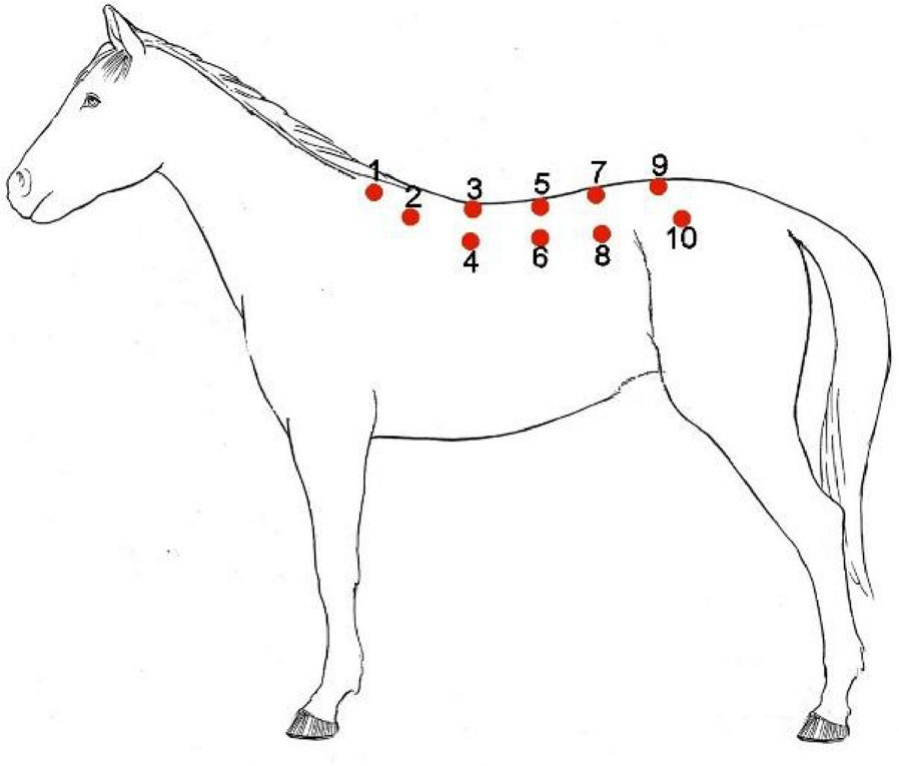
A left side schematic representation of the 10 bilateral thoracolumbar sites

**Figure 2 (abstract PO. 19) Figw:**
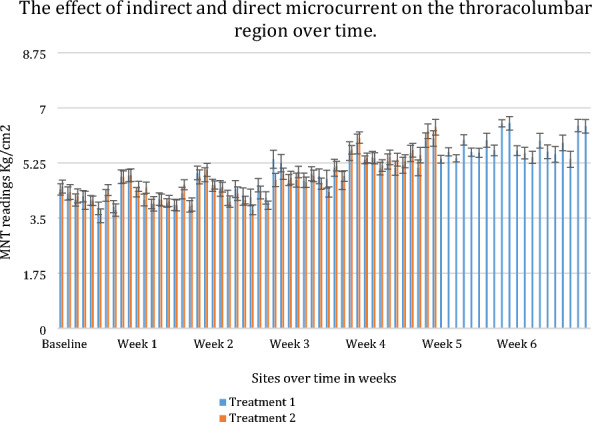
The effect of treatment 1 (indirect) and treatment 2 (direct) on all sites over the assessment time in weeks. MNT readings (kg/cm2) per site, values displayed as mean ± s.e obtained through RM-ANOVA

**Figure 3 (abstract PO. 19) Figx:**
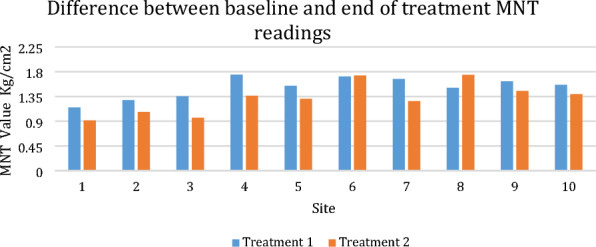
Absolute values of change from baseline to end of intervention MNT readings (pooled) per treatment group. One-way ANOVA showed time was significant P < 0.001 and treatments not significant P.964. Treatment/time P.225

**Table 1 (abstract PO. 19) Tabg:** The interaction of temperature on MNT values per site per degree increase, s.e and P value

Site	MNT + per ^o^C	s.e	P value
1	0.11	0.020	< 0.001
2	0.13	0.024	< 0.001
3	0.14	0.023	< 0.001
4	0.16	0.027	< 0.001
5	0.12	0.023	< 0.001
6	0.11	0.026	< 0.001
7	0.13	0.026	< 0.001
8	0.14	0.025	< 0.001
9	0.11	0.028	< 0.001
10	0.12	0.029	< 0.001

**Figure 4 (abstract PO. 19) Figy:**
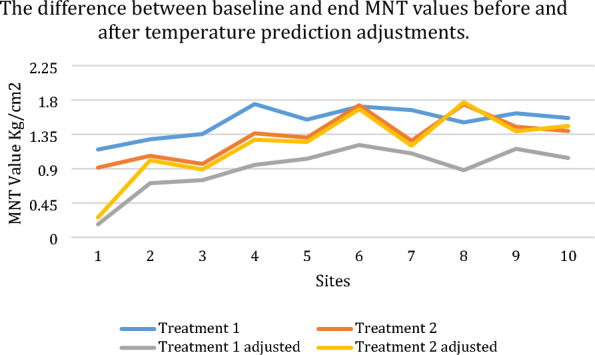
The effect size, represented by a comparison of mean difference of improvement between groups before and after temperature prediction adjustments (obtained from Unbalanced – ANOVA)

**Conclusion:** Direct and indirect microcurrent were unable to increase MNT values in the healthy ridden horse. Temperature affected MNT values.


**References**
Mayaki AM, Intan-Shameha AR, Noraniza MA, Mazlina M, Adamu L, Abdullah R. Clinical investigation of back disorders in horses: a retrospective study (2002–2017). Vet World. 2019; 12:377–81.Riccio B, Fraschetto C, Villanueva J, Cantatore F, Bertuglia, A. Two multicenter surveys on equine back-pain 10 years a part. Front Vet Sci., 2018; 5:195Haussler KK, Erb HN. Pressure algometry for the detection of induced back pain in horses: a preliminary study. Equine Vet J. 2006; 38:76–81.Dyson S, Ellis AD, Mackechnie‐Guire R, Douglas J, Bondi A, Harris P. The influence of rider: horse bodyweight ratio and rider‐horse‐saddle fit on equine gait and behaviour: A pilot study. Equine Vet Educ; 2020; 32:527–39McLean AN, McGreevy PD. Horse-training techniques that may defy the principles of learning theory and compromise welfare. J Vet Behav: Clinical applications and research. 2010; 5:187–95.Trager LR, Funk RA, Clapp KS, Dahlgren LA, Werre SR, Hodgson DR. et al., Extracorporeal shockwave therapy raises mechanical nociceptive threshold in horses with thoracolumbar pain. Equine Vet J. 2020; 52:250–7.Kang JI, Jeong DK. Choi H. Effects of microcurrent and cryotherapy on C-reactive protein levels and muscle tone of patients with rotator cuff reconstruction. J Phys Ther Sci. 2018. 30;37–41.Park JW, Kwak J, Lee S. Microcurrent electrical neuromuscular stimulation to improve myofascial neck pain and stiffness. Ann Phys Rehabil. 2018; 61:108.Hiroshige Y, Watanabe D, Aibara C, Kanzaki K, Matsunaga S. aWada M. Theefficacy of microcurrent therapy on eccentric contraction-induced muscle damage inrat fast-twitch skeletal muscle. Open J Appl Sci. 2018; 8:89.Rexing J, Dunning D, Siegel A.M, Knap K. Werbe B. Effects of cold compression, bandaging, and microcurrent electrical therapy after cranial cruciate ligament repair in dogs. Vet Surg. 2010; 39:54–58.Varcoe‐Cocks K, Sagar KN, Jeffcott LB, McGowan CM. Pressure algometry to quantify muscle pain in racehorses with suspected sacroiliac dysfunction. Equine Vet J. 2006; 38:558–62.Menke ES, Blom G, van Loon JP. Back W. Pressure algometry in Icelandic horses: interexaminer and intraexaminer reliability. J Equine Vet Sci. 2016; 36:26–31.Heus P, Van Oossanen G, Van Dierendonck MC. aBack W. A pressure algometer is a useful tool to objectively monitor the effect of diagnostic palpation by a physiotherapist in warmblood horses. J Equine Vet Sci. 2010; 30:310–21.Love EJ, Murrell J. aWhay HR. Thermal and mechanical nociceptive threshold testing in horses: a review. Vet Anaesth Analg. 2011; 3:3–14.


## PO. 32 Investigating the effect of four water depths on canine forelimb kinematics during walking on an underwater treadmill

### **Katherine Seychell**^1^ and Gillian Tabor^1^

#### ^1^Hartpury University, Gloucester, UK

##### **Correspondence:** Katherine Seychell (kate.seychell@gmail.com)

*Acta Veterinaria Scandinavica* 2023, **65(Suppl 1)**:PO. 32

**Background:** Given the multiple orthopaedic and neurological conditions affecting forelimb kinematics, it is vital to understand the impact of altering specific parameters on the underwater treadmill (UWTM), to improve clinical outcomes based on individual needs. The properties of water provide multiple advantages on canine limb kinematic and stride parameters [1–4]. However, research which can quantitatively inform evidence-based practice regarding forelimb kinematics utilising an UWTM, is limited. The aims of this study were to objectively obtain baseline forelimb range of movement (carpus, elbow and shoulder), stride length and frequency measurements in healthy canine subjects at differing water depths, to provide guidance and assist therapists selecting appropriate parameters for their canine patient.

**Materials and methods:** Kinematic analysis (2D) was utilised to assess range of movement of the canine shoulder, elbow, and carpus joints, alongside stride length and frequency. Eight medium to large breed dogs participated, free from musculoskeletal abnormalities, neurological or degenerative diseases. Reflective markers were placed on anatomical landmarks (dorsal border of spine of scapular, greater tubercle of the humerus, lateral epicondyle of the humerus, ulnar styloid process) and dogs walked at 2.4 m/s for 150-s at four water depths. Digital video cameras captured the data, and a video kinematic analysis tool was utilised to examine forelimb kinematics. A one-way repeated-measures ANOVA determined the effect of water depth on the range of movement of a canine’s forelimb, stride length and frequency. Bonferroni correction was applied, where applicable.

**Results:** Carpus and shoulder peak flexion (PF) significantly increased when walking at various depths on the UWTM (Table 1). Carpus peak flexion was significantly lower when walking on the dry, with flexion increasing at all other water depths and most peak flexion produced at the mid-ulnar depth. Shoulder PF increased with higher water depths, with most shoulder PF produced at mid-humerus water level. Water depth did not have a significant effect on peak flexion at the elbow, extension of all three forelimb joints, stride length or frequency.

**Table 1 (abstract PO. 32) Tabh:**
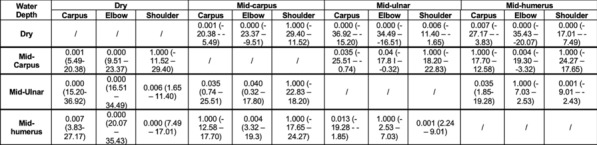
Significant values for pairwise comparisons for peak flexion at different water heights with confidence intervals in parenthesis

**Conclusion:** This investigation demonstrates the effect of differing water depths on range of movement of the forelimb joints (carpus, elbow and shoulder) and forelimb stride parameters. The findings illustrate the beneficial effects of adapting UWTM depth on range of movement, providing a greater insight into subsequent canine forelimb kinematics. The findings also provide therapists with valuable information for delivering hydrotherapy treatment to dogs with neuromusculoskeletal pathologies and provide further information on canine forelimb kinematics, which may benefit future research.


**References**
Levine D, Marcellin-Little D, Millis D, Tragauer V, Osborn JA. Effects of partial immersion in water on vertical ground reaction forces and weight distribution in dogs. Vet Res Pub. 2010; 71:1413–6.Marsolais GS. Dvorak G. Conzemius MG. Effects of postoperative rehabilitation on limb function after cranial cruciate ligament repair in dogs. J Am Vet Med Assoc. 2002; 220:1325–30.Speer K. Cavanaugh J. Warren R. Day L. Wickiewicz T. A role for hydrotherapy in shoulder rehabilitation. Am J Sports Med. 1993; 21:850–3.Barnicoat F, Wills A. P. Effect of water depth on limb kinematics of the domestic dog (Canis lupus familiaris) during underwater treadmill exercise. Comp Exerc Physiol. 2016; 12:119.


## Virtual Poster Presentations

## PO. 20 An investigation into the effect of canine footwear on locomotion

### Emily Cooke^1^, **Sarah Roberts**^1^

#### ^1^Animal Health, Behavior and Welfare Department, Harper Adams University, Edgmond, Shropshire, TF10 8NB

##### **Correspondence:** Sarah Roberts (broberts@harper-adams.ac.uk)

*Acta Veterinaria Scandinavica* 2023, **65(Suppl 1)**:PO. 20.

**Background:** Boots are used on dogs to protect feet from injury while providing support, often with dogs working on uneven surfaces, such as search and rescue dogs [1] or for neurological cases where paw abrasion can occur [2]. Gait, as a result of internal and external factors, such as the walking surface, the musculoskeletal system, and peripheral nervous system, must work in conjunction to adapt to this [3]. The effects of external factors, such as shoes, have been researched extensively within the human and equine fields. However, there is limited research on the impact of boots on canine locomotion.

**Materials and methods:** Ten healthy dogs were used in a cross-over design study. Participants were given time to habituate to equipment, researcher, and handler. Each dog performed five passes of the Gait4Mat per regimen, with at least three gait cycles in each pass. Three regimens were conducted on different days: ‘1’ without boots, ‘2’ with boot type A (PawZ boots), and ‘3’ with boot type B (Ruffwear Summit Trex boots). Participants returned on a separate day for each regimen, 24 h or more after the previous data collection.

**Results:** Mean GAIT4Dog Lameness Scores (GLS) were analysed using two-way ANOVA to establish the significance between boots, and fore-limb and hind-limbs. Paired t-tests were used, comparing gait parameters and assessing symmetry. There was no significant difference found between total pressure index and boot application (P > 0.05). A two-way ANOVA indicated that boot type did not have a significant difference on GLS (P > 0.05). However, there was a significantly higher GLS in the hind-limbs than the fore-limbs (P < 0.001) (Fig. 1); indicating canines off-loading weight to the hind-limbs when boots were applied, with boot type B demonstrating a significant greater response than boot type A (P = 0.019) when applied. The interaction (P = 0.019) between boot type and application on either FLs or HLs on GLS does indicate boots do have a significant effect on canine locomotion as weight redistribution is occurring.

**Figure 1 (abstract PO. 20) Figz:**
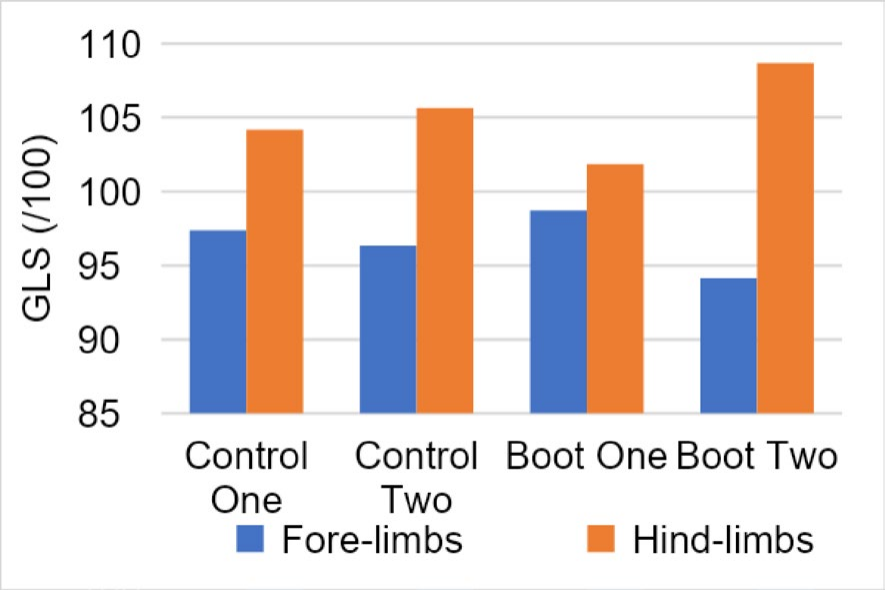
Comparison of fore-limb and hind-limb GLS for each treatment

**Figure 2 (abstract PO. 20) Figaa:**
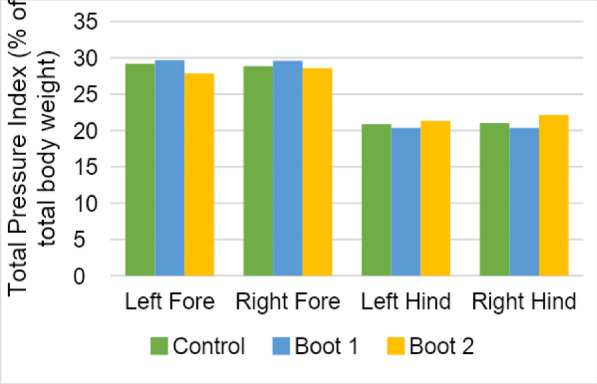
Comparison of fore-limb and hind-limb TPI for each treatment

**Conclusion:** Boot type does not alter limb loading in terms of total pressure index. However, GLS indicates altered locomotion. This is one of the first studies into the effect of dog boots on limb loading and canine locomotion. Therefore, further research should be conducted on alternative dog breeds with less natural variance in a more controlled environment.


**References**
Marcellin-Little DJ, Levine D. Devices for ambulation assistance in companion animals. In. Millis, D. and Levine, D. ed. Canine rehabilitation and physical therapy. 2^nd^ ed. Philadelphia: Saunders. 2014, pp.305–11.Sims C, Waldron R, Marcellin-Little DJ. Rehabilitation and physical therapy for the neurologic veterinary patient. Vet Clin North Am Small Anim Pract. 2015;45:123–43.Gillette RL, Angle TC. Canine locomotion analysis. In: Millis D. and Levine D. ed. Canine rehabilitation and physical therapy. 2^nd^ ed. Philadelphia: Saunders. 2014. pp.201–10.


## PO. 21 The awareness, understanding, and opinions of animal physiotherapy amongst veterinarians based in Hong Kong (SAR)

### **Charlotte Davies**^1^, Jo Ireland^2^

#### ^1^Physiotherapy Department, Joint Dynamics, 5th Floor Asia Standard Tower, 59-65 Queens Road Central, Central and Western District, Hong Kong; ^2^Institute of Infection, Veterinary and Ecological Sciences, Department of Equine Clinical Science, University of Liverpool, Leahurst Campus, Neston, Cheshire, CH64 7TE, United Kingdom

##### **Correspondence:** Charlotte Davies (charlotte.davies@jointdynamics.com.hk)

*Acta Veterinaria Scandinavica* 2023, **65(Suppl 1)**:PO. 21

**Background:** Within human healthcare, physiotherapy is a well-recognized allied profession [1]. Animal physiotherapy is an emerging profession in veterinary medicine, and studies in Western countries suggest awareness amongst veterinarians is increasing [2, 3]. Animal physiotherapy in Hong Kong has not previously been researched.

**Materials and methods:** In this cross-sectional study, an online questionnaire was designed to investigate Hong Kong veterinarians’ understanding of animal physiotherapy and perceived barriers to referral. All veterinary clinics listed on the Agriculture, Fisheries and Conservation Department government website (n = 183) and The Hong Kong Jockey Club were emailed a study invitation to distribute to all registered veterinarians within their clinic. Descriptive statistics were produced and associations between variables were evaluated using Mann–Whitney U, Kruskal–Wallis, Chi-squared and Fisher’s exact tests [4].

**Results:** Fifty responses were received, with 72.0% of respondents from small-animal practice (n = 36/50) and 22.0% from equine (n = 11/50). Overall, 84.0% of respondents (n = 42/50) were aware of animal physiotherapy as a profession, and 76.0% (n = 38/50) had referred ≥ 1 case for physiotherapy in the past year. Respondents had qualified as a veterinarian a median of 17 years previously, and neither time since graduation (P = 0.61) nor having worked in Western countries (P = 0.07) were associated with awareness of animal physiotherapy. Type of practice was associated with referral (P = 0.004): 86.1% of small-animal veterinarians (n = 31/36) and 63.6% of equine veterinarians (n = 7/11) had referred ≥ 1 case in the previous year, while none of the three respondents treating other species reported having done so. The most commonly reported barrier to referral was clients being unwilling to pay for physiotherapy (37.5%; n = 19/50), and veterinarians considered lack of owner awareness of animal physiotherapy (36.0%; n = 18/50) and difficulty in transporting patients 16.0% (n = 8/50) as barriers to attending physiotherapy. Problems that veterinarians most frequently believed physiotherapy could help were all musculoskeletal conditions (Table 1). There was uncertainty about whether some physiotherapy techniques or treatment modalities could be used for effective treatment of veterinary patients, with respondents frequently being unsure regarding the use of respiratory assistive devices (60.0%; n = 30/50) and chest clearance techniques (48.0%; n = 24/50).

**Table 1 (abstract PO. 21) Tabi:** Respondents’ considerations of whether animal physiotherapy could aid treatment from a list of pathologies in a survey of 50 veterinarians in Hong Kong

Problem	Yes Frequency (%)	No Frequency (%)	Unsure Frequency (%)
Joint stiffness/restrictions	50 (100%)	0 (0%)	0 (0%)
Muscle atrophy	49 (98%)	0 (0%)	1 (2%)
Musculoskeletal pain	48 (96%)	0 (0%)	2 (4%)
Spinal Injury	46 (92%)	3 (6%)	1(2%)
Ligament Injuries	46 (92%)	3 (6%)	1 (2%)
Muscle tears	45 (90%)	1 (2%)	4 (8%)
Tendon Injuries	45 (90%)	4 (8%)	1 (2%)
Degenerative joint disease	43 (86%)	7 (14%)	0 (0%)
Neuropathic pain	41 (82%)	5 (10%)	4 (8%)
Neurological issues	39 (78%)	5 (10%)	6 (12%)
Amputation	36 (72%)	8 (16%)	6 (12%)
Poor performance	34 (68%)	5 (10%)	11 (22%)
Fractures	30 (60%)	14 (28%)	6 (12%)
Oedema	28 (56%)	9 (18%)	13 (26%)
Obesity	27 (54%)	14 (28%)	9 (18%)
Haematomas	19 (38%)	14 (28%)	17 (34%)
Wound healing	16 (32%)	14 (28%)	20 (40%)
Cardiorespiratory issues	10 (20%)	22 (44%)	18 (36%)

**Conclusions**: Although most Hong Kong veterinarians in this study were aware of animal physiotherapy, there were several areas of uncertainty regarding pathologies and treatment modalities, suggesting physiotherapy may be underutilized for some conditions. Further research to determine the best ways to increase veterinarian understanding of specialist areas of physiotherapy and to broaden referral into these areas is warranted.


**Acknowledgements**


The authors gratefully acknowledge all participating veterinarians.


**References**
Vardhan S, Sharma M, Lamba D, Raj I. Awareness of physiotherapy among general practitioners in a district of Punjab. Int J Curr Res. 2018, 10:69154–6.Doyle A, Horgan N. Perceptions of animal physiotherapy amongst Irish veterinary surgeons. Ir Vet J. 2006, 59:85–9Ryan T, Finn A. Observations on a survey of veterinary students' perceptions of animal physiotherapy. Ir Vet J; 2000; 53:143–5.Boden L. Clinical studies utilising ordinal data: pitfalls in the analysis and interpretation of clinical grading systems. Eq Vet J; 2011; 43: 383–7.


## PO. 22 Effects of β-hydroxy-β-methylbutyrate on muscle mass of dogs who underwent orthopedic surgeries

### **Morito Ogasawara**^1^, Toshihiko Fushimi^2^

#### ^1^Animal Rehabilitation Medicine Laboratory, Tokyo, Japan; ^2^Fushimi Animal Hospital, Tochigi, Japan

##### **Correspondence:** Morito Ogasawara (armlab.japan@gmail.com)

*Acta Veterinaria Scandinavica* 2023, **65(Suppl 1)**:PO. 22

**Background:** β-hydroxy-β-methylbutyrate (HMB) is a metabolite of leucine that is known as one of the essential amino acids that activates the mammalian target of rapamycin complex 1 and increases protein synthesis [1, 2]. This study aimed to investigate the effects of HMB on muscle mass in dogs after orthopedic surgeries.

**Materials and methods:** 18 dogs that underwent hind limb orthopedic surgeries (9 MPL, 3 TTA, 3 TPLO, 2 TTTA, and 1 CBLO) were divided into 2 groups. 8 dogs were fed with HMB supplementation, and 10 were fed without HMB. Muscle mass of hind limbs was measured on days 0, 4, 10, and 30 postoperatively. Changing rates of muscle mass in 2 groups were compared by t-test, and the correlation between the changing rates of muscle mass and the daily protein intakes was assessed.

**Results:** Changing rates of muscle mass in the group with HMB were higher than in the group without HMB on Day10 (mean value of + 1.5% and -1.8% respectively, P < 0.05) and Day30 (mean value of + 6.8% and -2.1% respectively, P < 0.05). Also, daily protein intakes were potentially weakly correlated with the changing rates of muscle mass at Day10 (r = 0.206).

**Conclusion:** HMB supplementation could prevent the muscle mass loss of the dogs who underwent orthopedic surgeries, postoperatively. Mean intakes of daily protein were the same in 2 groups. That may indicate that HMB increases protein synthesis and help better recovery.


**References**
Yasutomi K, Yukino H, Ran U, Ryoji Y, Shinji M. Regulation of skeletal muscle function by amino acids. Nutrients 2020; 12:261; doi:10.3390.Beaudart C, Dawson A, Shaw S.C, Harvey N.C, Kanis J., Binkley N., et al.; Nutrition and physical activity in the prevention and treatment of sarcopenia: a systematic review. Osteoporos Int.2017;28:1817–33.


## PO. 23 Canine hypermobility: a positive correlation found between skin extensibility and joint range in dogs

### **Amy Kings**^1,2^

#### ^1^Hartpury College, Hartpury, United Kingdom; ^2^Win Clinic Ltd, Wellington, United Kingdom

##### Correspondence: Amy King (amy@ngphysio.co.uk)

*Acta Veterinaria Scandinavica* 2023, **65(Suppl 1)**:PO. 23

**Background:** The Beighton scoring system is a commonly used tool for identifying hypermobility [1–3]. Evidence shows that in a canine population there are links between joint laxity and pathologies such as hip dysplasia and patella luxation, but there is little evidence to connect the systemic presentation of hypermobility associated with variation in collagen histology [4]. Ueda et al. demonstrated a positive correlation between abnormal collagen fibril distribution and a high SEI (more than the 14.5% reference value) in dogs with patella luxation [5, 6]. This study aimed to investigate SEI as part of the clinical picture of hypermobility. To look for evidence of hypermobility we investigated for correlation between skin extensibility index (SEI) and shoulder abduction or stifle extension ranges in healthy dogs.

**Materials and methods:** A correlational study of 22 mixed breed dogs split into equal groups (n = 11) of SEI ≥ 14.5% indicating increased skin extensibility, or SEI < 14.5% without increased skin extensibility [6]. Dogs were aged between 18–84 months and weighed between 8.7-35 kg. Shoulder abduction and stifle extension of bilateral limbs were measured in conscious dogs by an ACPAT veterinary physiotherapist using a goniometer. SEI was measured using a previously established method as seen in Fig. 1.0.

**Results:** A correlation was found between SEI and both shoulder abduction (ρ = 0.512; P < 0.0001) and stifle extension (ρ = 0.600; P < 0.0001). T-tests showed statistically significant differences between the mean values across the two groups. Mean joint ranges and SEI scores are seen in Table 1.

**Figure 1 (abstract PO. 23) Figab:**
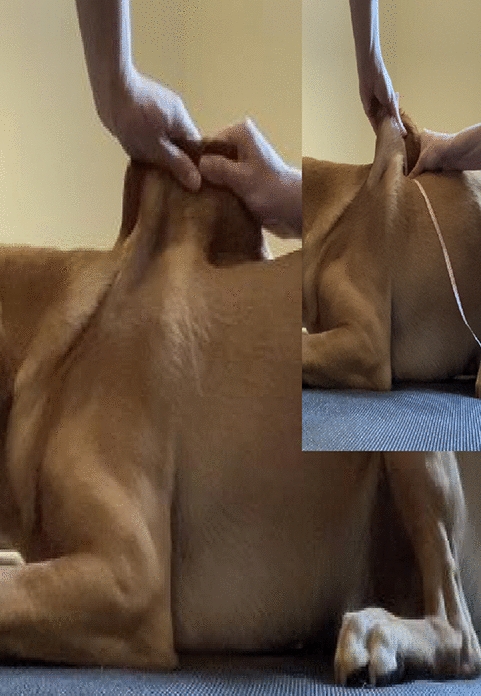
Assessment of skin extensibility index

**Table Tabj:** Table 1 (abstract PO. 23)

	Left stifle extension (°)	Right stifle Extension (°)	Combined Stifle Extension (°)	Left Shoulder Abduction (°)	Right Shoulder Abduction (°)	Combined Shoulder Abduction (°)	SEI (%)
All dogs	180 ± 5.56	179.82 ± 6.77	180.08 ± 6.13	39.48 ± 6.3°	41.48 ± 7.71	40.48 ± 7.04	13.20 ± 3.12
SEI ≥ 14.5% group	182.42 ± 5.14	182.42 ± 5.86	183.24 ± 5.45	43.39 ± 3.75	45.36 ± 6.10	44.38 ± 5.04	15.56 ± 0.93
SEI < 14.5% group	177.48 ± 4.43	176.36 ± 5.83	176.92 ± 5.08	37.61 ± 7.40	35.58 ± 5.99	36.59 ± 6.65	10.85 ± 2.71

**Conclusion:** Joint range and SEI are positively correlated, and significantly higher joint ranges are found in dogs with an SEI of ≥ 14.5%. These parameters could be used as part of an assessment to indicate the presence of hypermobility in dogs given the previously demonstrated association between abnormal collagen fiber distribution, SEI, and patella luxation in dogs. Further research in a juvenile population of dogs would improve the relevance of these findings to the early diagnosis of hypermobility conditions.


**References**
Hakim A, Grahame R. Joint hypermobility. Best Prac Res Clin Rheumatol; 2003; 17:989–1004.Clinch J, Deere K, Sayers A, Palmer S, Riddoch C, Tobias J et al. Epidemiology of generalized joint laxity (hypermobility) in fourteen‐year‐old children from the UK: A population‐based evaluation. Arthritis Rheum. 2011;63:2819–27.Bloom L, Byers P, Francomano C, Tinkle B, Malfait F. The international consortium on the Ehlers-Danlos syndromes. J Med Genet. Part C: Seminars in Medical Genetics. 2017;175:5–7.Madsen J, Oxlund H, Svalastoga E, Schwarz P. Collagen type III:I composition in hip joints of dogs susceptible to nip dysplasia. J Small Anim Pract. 1994;35:625–8.Ueda K, Kawai T, Senoo H, Shimizu A, Ishiko A, Nagata M. Histopathological and electron microscopic study in dogs with patellar luxation and skin hyperextensibility. J Vet Med Sci. 2018;80:1309–16Freeman L, Hegreberg G, Robinette J. Ehlers-Danlos syndrome in dogs and cats. Semin Vet Med Surg (Small Anim). 1987;2:221–7.Bowen J, Fatjó J, Serpell J, Bulbena-Cabré A, Leighton E, Bulbena A. First evidence for an association between joint hypermobility and excitability in a non-human species, the domestic dog. Sci Rep 2019;9:8629Briggs J, McCormack M, Hakim A, Grahame R. Injury and joint hypermobility syndrome in ballet dancers–a 5-year follow-up. Rheumatology. 2009;48:1613–14.Nathan J, Davies K, Swaine I. Hypermobility and sports injury. BMJ Open Sport & Exercise Medicine. 2018;4.


## PO. 24 Comparing veterinary professionals’ opinions on the use of physiotherapy for surgically managed cranial cruciate ligament disease patients

### Hannah Taylor^1^, **Sarah Roberts**^1^

#### ^1^Animal Health, Behavior and Welfare Department, Harper Adams University, Edgmond, Shropshire, TF10 8NB, UK

##### **Correspondence:** Sarah Roberts (broberts@harper-adams.ac.uk)

*Acta Veterinaria Scandinavica* 2023, **65(Suppl 1)**:PO. 24

**Background:** Cranial cruciate ligament disease (CCLD) is the fourth most common musculoskeletal condition in the UK canine population, estimated to affect 1.24% of dogs [1]. The management of CCLD has advanced with the development of osteotomy surgeries; this progression highlights the importance of post-operative physiotherapy [3]. However, the current implementation of post-operative physiotherapy in CCLD patients is lacking in research.

**Materials and methods:** A questionnaire was developed using Bristol Online Surveys and distributed by email to 1078 veterinary practices on the Royal College of Veterinary Surgeons (RCVS) ‘Find a Vet’ database, 207 members of the ‘National Association of Veterinary Physiotherapists’ and 156 members of the ‘Canine Hydrotherapy Association’. The questionnaire used categorical, multiple choice, list, ranking, and open questions to achieve the study objectives. Confidence intervals were performed on single categorical questions to calculate the range within which the general population would fall, to a 95% confidence level. Open questions were evaluated by content analysis to identify common ideas and themes. Descriptive statistics (profession, gender, years since qualification) were cross-tabulated against categorical and coded open questions to identify relationships between responses using chi-squared analysis and Fisher’s exact test.

**Results:** The questionnaire gained responses from 50 veterinarians, 35 veterinary physiotherapists, and 29 hydrotherapists. The majority of CCLD patients were reportedly treated surgically by Tibial Plateau Levelling Osteotomy (TPLO) 96% of respondents have referred CCLD patients for physiotherapy, with 41.67% referring frequently (Fig. 1). Surgically managed patients were reportedly referred for physiotherapy more frequently than conservatively managed patients. Significantly more patients treated with osteotomies (P < 0.01), particularly TPLO (P < 0.05), were referred for physiotherapy than those undergoing soft tissue techniques. A significant difference existed between veterinary physiotherapists’ current and recommended commencement of physiotherapy (P < 0.05), with 41.2% recommending beginning physiotherapy at an earlier stage (Fig. 2).

**Figure 1 (abstract PO. 24) Figac:**
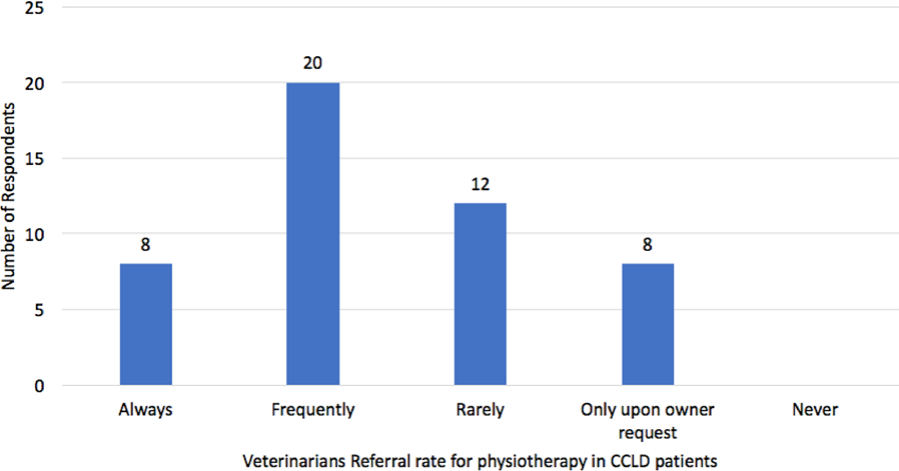
Bar Chart illustrating frequency of veterinary referral of CCLD patients for physiotherapy

**Figure 2 (abstract PO. 24) Figad:**
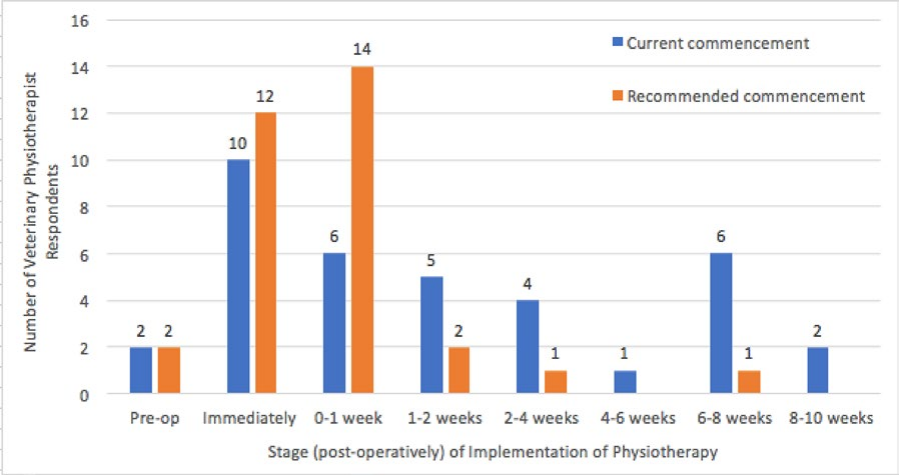
Bar Chart comparing when physiotherapy is commencing in surgically managed CCLD patients to when Veterinary Physiotherapists would recommend commencing physiotherapy

**Conclusion:** The large variation in the initiation of physiotherapy, physiotherapy techniques employed, and their perceived benefits suggests that the optimum management of CCLD patients has not yet been established. Further research is required to establish optimum management protocols; however, difficulty arises from different therapies commencing at different stages and individual variations in the rate of healing.


**References**
O'Neill DG, Church DB, McGreevy PD, Thomson PC, Brodbelt DC. Prevalence of disorders recorded in cats attending primary-care veterinary practices in England. Vet J.2014; 202:286–91.Witsberger TH, Villamil JA, Schultz LG, Hahn AW, Cook JL. Prevalence of and risk factors for hip dysplasia and cranial cruciate ligament deficiency in dogs. J Am Vet Med Assoc.; 2008; 232:1818–24.Monk M, Preston C, McGowan C. Effects of early intensive postoperative physiotherapy on limb function after tibial plateau leveling osteotomy in dogs with deficiency of the cranial cruciate ligament. Am J Vet Res.2006; 67:529–56.


## PO. 25 The effect of leg weights on stride length and range of motion in hind limbs of healthy dogs at the walk

### **Anna Eriksson Svensson**^1^, Ann Essner^2^, Wim Grooten^1^

#### ^1^Karolinska Institutet, Division of Physiotherapy, Department of Neurobiology, Care Sciences and Society, Stockholm, Sweden; ^2^Uppsala Universitet, Department of Women´s and Children´s Health, Uppsala, Sweden; ^1^Karolinska Institutet, Division of Physiotherapy, Department of Neurobiology, Care Sciences and Society, Stockholm, Sweden.

##### Correspondance: Ann Essner (ann.essner@evidensia.se)

*Acta Veterinaria Scandinavica* 2023, **65(Suppl 1)**:PO. 25

**Background:** Using leg weights (LW), both with and without weights is a training method in the re- and prehabilitation of dogs. Previous research indicates increased range of motion (ROM) in the hind legs when using LW corresponding to 0.5% body mass (BM) in dogs at trot [1]. The effect on ROM and stride length with LW at a walk and with lower weights is unclear, so as with empty LW. This study aimed to investigate the effects of empty LW and LW corresponding to 0.4% BM on stride length and ROM in hind limbs in healthy dogs walking on a treadmill.

**Materials and methods:** Six dogs with a mean body weight of 23.8 kg (SD 5.7) and a mean age of 4.5 years (SD 2.7) were recorded at a sampling frequency of 30 frames per second in the sagittal plane when walking at a comfortable speed on a treadmill without LW, with empty LW proximal to the tarsus (Fig. 1) and with the added mass of 0.4% BM in the Before filming, The dogs had been acclimated the treadmill and the leg weights before filming. The placement of markers was standardized (Fig. 1). The recordings were analyzed in the movement analysis software Kinovea. Differences were estimated with the Friedmans test and post-hoc Wilcoxon Signed Rank test (P < 0.05). The dog's stress level and perceived exertion were also estimated during the study using the FAS-scale [2] and a perceived exertion scale for dogs [3].

**Figure 1 (abstract PO. 25) Figae:**
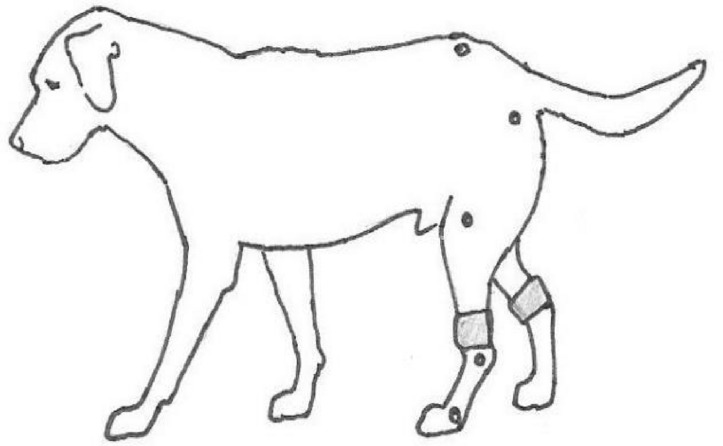
Placement of LW and markers in the dogs

**Results:** During a walk on a treadmill with empty LW, stride length in the hind limbs increased significantly (P = 0.027) compared to no LW applied. The mean difference was 1.7 cm (SD 3.5 without LW and 3.7 with empty LW). There was no difference between LW corresponding to 0.4% BM compared to no LW. Further, there was no significant change in ROM in tarsal, stifle or hip joints with empty or loaded LW. The methods in this study were well tolerated by all dogs.

**Conclusion:** Empty LW can be used to increase stride length in healthy dogs walking on a treadmill. Further studies are needed to evaluate the effect of LW on ROM since the sample size was small in the present study. The methods here were well tolerated by the dogs and can be used in a protocol in future studies.


**References**
Kilbourne B M, Carrier D R. Manipulated changes in limb mass and rotational inertia in trotting dogs and their effect on limb kinematics. J Exp. Zool. 2016;325A:665–74.Fear, anxiety and stress scale (FAS), www.fearfreepets.com.Swanson KDJ, Harper TAM, Mc Michael M, Fries RC, Lascola KM, Chandler C, et al. Development of a perceived exertion scale for dogs using selected physiologic parameters. J Small Anim Pract. 2019;60:247–53.


## PO. 26 The effect different water levels have on muscle activation of the canine biceps femoris and vastus lateralis muscles in an underwater treadmill

### Amy Charnock^1^, **Sarah Roberts**^1^

#### ^1^Animal Health, Behavior and Welfare Department, Harper Adams University, Edgmond, Shropshire, TF10 8NB, UK

##### **Correspondence:** Sarah Roberts (broberts@harper-adams.ac.uk)

*Acta Veterinaria Scandinavica* 2023, **65(Suppl 1)**:PO. 26.

**Background:** Aquatic therapy uses the properties of water for the rehabilitation and conditioning of the canine patient [1]. It is important that veterinary physiotherapists and hydrotherapists understand the physiological effects underwater treadmills have, so that rehabilitation plans can be suitably tailored for the individual. Gait adaptations caused by walking on an underwater treadmill have been extensively researched in dogs and horses, with these effects presumably altering muscle activation [2–4]. Currently, only one study has investigated the effects underwater treadmills have on canine muscle activity, which specifically looked at the Gluteus medius and longissimus dorsi muscles [5]. It has previously been identified that more research into canine muscle activity using surface electromyography is required and with the quadriceps femoris and hamstrings making up a large portion of the hindlimb muscle mass [6, 7].

**Materials and methods:** Nine healthy Labrador Retriever and Labrador Retriever crosses were included in the study. Prior to data collection, all dogs were habituated to the underwater treadmill. Surface electromyography was used to detect muscle activity by placing electrodes over the vastus lateralis and biceps femoris muscle bodies. The treadmill was set at a comfortable walking speed for each dog (1.6 ± 0.12 m/s) with no water and water set at the height of the tarsal, stifle, and hip joints. Unfortunately, due to water interference, data was unable to be recorded at hip height. As muscle activation occurred at different stages in the gait cycle, the data was cropped at three points where the muscles were activated. This data was then transformed and analyzed using ANOVA to test for a significant difference between water height and muscle activity.

**Results:** While water height influenced muscle activity, no significant difference was found for the biceps femoris muscle (P = 0.842), or the vastus lateralis muscle (P = 0.715). Results found that water height had opposite effects on muscle activity, with the biceps femoris muscle increasing and then decreasing in activity (Table 1), whilst the vastus lateralis muscle decreased and then increased in activity (Table 2).

**Table 1 (abstract PO. 26) Tabk:** Mean muscle activation for the biceps femoris muscle

Water Height	Mean Muscle Activity (V)
No Water	0.0923
Tarsus	0.0973
Stifle	0.0798

**Table 2 (abstract PO. 26) Tabl:** Mean muscle activation for the vastus lateralis muscle

Water Height	Mean Muscle Activity (V)
No Water	0.0283
Tarsus	0.0278
Stifle	0.0295

**Conclusion:** This study demonstrates that while water height affects muscle activity, it does not cause a significant difference. It highlighted the fact that the biceps femoris and vastus lateralis muscles respond differently to water height, in relation to the muscle's action in the gait cycle. Future research is required with larger samples to establish if there is a significant difference in muscle activation.


**References**
Houlding B. Canine hydrotherapy: where are we now? Vet Rec. 2011; 168:405–6.Barnicoat F, Wills AP. Effect of water depth on limb kinematics of the domestic dog (*Canis lupus familiaris*) during underwater treadmill exercise. Comp Exerc Physiol. 2016; 12:199–207.Nankervis KJ, Lefrancois K. A comparison of protraction-retraction of the distal limb during treadmill and water treadmill walking in horses. J Equine Vet Sci. 2018; 70:57–62.Mendez-Angulo JL. Effect of water on kinematics of healthy horses walked on an aquatic treadmill compared to conventional rehabilitation techniques [dissertation]. Minneapolis: University of Minnesota; 2012.Parkinson S, Wills AP, Tabor G, Williams JM. Effect of water depth on muscle activity of dogs when walking on a water treadmill. Comp Exerc Physiol. 2018; 14:79–89.Valentin S, Zsoldos RR. Surface electromyography in animals: a systematic review. J Electromyogr Kinesiol. 2016; 28:167–83.Riegger-Krugh C, Millis DL, Weigel JP. Chapter 5: Canine Anatomy. In: Millis, DL, Levine D, editors. Canine Rehabilitation and Physical Therapy. 2^nd^ edition. Philadelphia: Saunders; 2014; 41–78.


## PO. 27 Preliminary study to investigate effect of dog positioning on range of motion of elbow and hip joints in healthy dogs

### Justyna Mucha, **Sarah Roberts**^1^

#### ^1^Animal Health, Behavior, and Welfare Department, Harper Adams University, Edgmond, Shropshire, TF10 8NB, UK

##### **Correspondence:** Sarah Roberts (broberts@harper-adams.ac.uk)

*Acta Veterinaria Scandinavica* 2023, **65(Suppl 1)**:PO. 27

**Background**: Range of motion (ROM) measurements of joints are used by physiotherapists to support the development of treatment plans and monitor progression after intervention [1]. ROM measurements in dogs are performed in both standing and lateral recumbency [2]. There are no studies investigating the effect of dog positioning on ROM. This requires further investigation to consider whether standing and lateral recumbency positions can be used interchangeably to make appropriate comparisons between patients [3].

**Materials and methods:** An experimental approach was chosen as the design, adapted from Jaeger et al. [4]. Healthy Labrador Retrievers were selected using convenience sampling, with the sample size of four being determined by utilizing the coefficient of variation from the aforementioned study [4]. Flexion and extension ROM of one elbow and the ipsilateral hip joint were measured with a universal goniometer (Figure 1) in standing and lateral recumbency positions. The side of measurement was designed to be randomly chosen by a draw for the first participating dog and alternated for each consecutive participating dog. All joint positions were measured three times each by one tester.

**Figure 1 (abstract PO. 27) Figaf:**
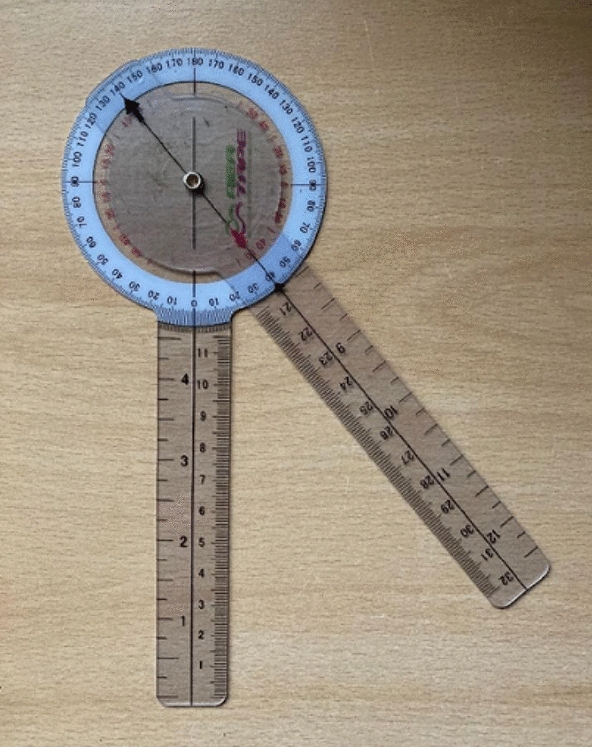
Universal goniometer

**Results:** The gross data measurements can be seen in Table 1. Mean, and standard deviation (SD) were calculated for data sets that were normally distributed, and median and interquartile ranges were calculated for data sets that were abnormally distributed. The parametric matched pairs t-test was used to determine if dog positioning had a statistically significant effect on the ROM measurements for data sets that were normally distributed, and the nonparametric Wilcoxon matched pairs test was used to determine if dog positioning had a statistically significant effect on the ROM measurements for data sets that were abnormally distributed. The results were considered statistically significant when the probability (P) was less than 0.05. Data from the elbow flexion measurements were highly skewed (1.05), and data from the elbow extension, hip flexion, and hip extension measurements were only slightly skewed (0.21, -0.09, and -0.11, respectively) (Table 2). None of the ROM measurements was statistically significantly affected by the dog positioning.

**Table 1 (abstract PO. 27) Tabm:** Elbow and hip range of motion measurements in flexion and extension taken in standing and lateral recumbency positions in four healthy dogs

Dog 1	Elbow Flexion Standing	Elbow Flexion Lateral Recumbency	Elbow Extension Standing	Elbow Extension Lateral Recumbency	Hip Flexion Standing	Hip Flexion Lateral Recumbency	Hip Extension Standing	Hip Extension Lateral Recumbency
Meas. 1	25	23	140	132	41	45	145	95
Meas. 2	25	23	148	135	46	51	145	104
Meas. 3	23	20	153	136	46	41	143	103
**Dog 2**								
Meas. 1	30	25	149	114	59	58	126	122
Meas. 2	32	27	149	130	60	54	125	120
Meas. 3	33	32	139	134	54	51	116	120
**Dog 3**								
Meas. 1	30	50	139	121	58	60	126	102
Meas. 2	32	39	138	135	50	64	127	100
Meas. 3	30	39	140	137	54	64	128	100
**Dog 4**								
Meas. 1	30	27	150	143	52	50	126	107
Meas. 2	28	25	122	133	50	68	130	110
Meas. 3	29	30	125	130	48	61	138	113

**Table 2 (abstract PO. 27) Tabn:** The statistical results of the effect of dog positioning on elbow and hip range of motion measurements in flexion and extension in four healthy dogs

Joint position	Probability	Mean (M)/ Median (Mdn)Standing (°)	Mean(M)/ Median (Mdn) Lateral Recumbency (°)	Standard Deviation(SD)/ Interquartile Range (IQR) Standing	Standard Deviation (SD)/ Interquartile Range (IQR) Lateral Recumbency
Elbow Flexion	0.875	29.8° (Mdn)	27.7° (Mdn)	27.8 – 30.9 (IQR)	26 – 31.7 (IQR)
Elbow Extension	0.145	141° (M)	131.7° (M)	± 6.75 (SD)	± 4.21 (SD)
Hip Flexion	0.278	51.5° (M)	55.6° (M)	± 5.71 (SD)	± 7.46 (SD)
Hip Extension	0.074	131.3° (M)	108° (M)	± 9.46 (SD)	± 9.52 (SD)

**Conclusions:** While in this study dog positioning did not have a statistically significant effect on flexion and extension ROM of the elbow and hip joints, due to the minimal sample size being utilized and large variations noted in gross data for standing measurements, a further larger study should be considered. Elbow flexion measurements were highly skewed (1.05), and data from the elbow extension, hip flexion, and hip extension measurements were only slightly skewed (0.21, -0.09, and -0.11, respectively) (Table 2). None of the ROM measurements was statistically significantly affected by the dog positioning.


**References**
Adair HS, Marcellin-Little DJ, Levine D; Validity and repeatability of goniometry in normal horses. Vet Comp Orthop Traumatol;2016; 29:314–9.Formenton MR, de Lima LG, Vassalo FG, Joaquim JGF, Rosseto LP, Fantoni DT. Goniometric assessment in French Bulldogs. Front Vet Sci. 2019; https://doi.org/10.3389/fvets.2019.00424Unver B, Karatosun V, Bakirhan S. Reliability of goniometric measurements of flexion in total knee arthroplasty patients: with special reference to the body position. J Phys Ther Sci.; 2009; 21:257–62.Jaegger G, Marcellin-Little D.,Levine D. Reliability of goniometry in Labrador Retrievers. Can J Comp Med Vet Sci.;2002;63;979–86.


## PO. 28 Surface electromyography of the Gluteus medius, Vastus lateralis, and gastrocnemius muscles in normal beagles during sit-to-stand and stand-to-sit motions

### **Kazuyuki Yoshikawa**^1^, Takio Kitazawa^2^, Tadashi Sano^2^, Takumi Ino^3^, Tomoya Miyasaka^3^

#### ^1^Japan Small Animal Medical Center, 1-10-4 Higashi Tokorozawa Wada, Tokorozawa-shi, Saitama, Japan 359-0025; ^2^Department of Veterinary Sciences, School of Veterinary Medicine, Rakuno Gakuen University, Ebetsu-shi, Hokkaido, Japan; ^3^Department of Physical Therapy, School of Health Sciences, Hokkaido University of Science, Teine-shi, Hokkaido, Japan

##### **Correspondence:** Kazuyuki Yoshikawa (kyoshikawa@jsamc.jp)

*Acta Veterinaria Scandinavica* 2023, **65(Suppl 1)**:PO. 28

**Background:** Sit-to-stand and stand-to-sit motions are essential for the daily life of animals. These motions are a therapeutic exercise following musculoskeletal and neurological disorders [1]. However, muscle activity of these motions has not been well examined yet. This study aimed to characterize muscle activities of the hindlimb extensors during sit-to-stand and stand-to-sit motions. For this purpose, we measured the Gluteus medius (GM), Vastus lateralis (VL), and Gastrocnemius (GC) muscle activities using surface electromyography (sEMG) because these are major extensor muscles of the hip, stifle, and tarsal joints.

**Materials and methods:** Six clinically sound beagles aged between 4 and 8 years were used. After being habituated to walking on a treadmill, we acquired data for 10 valid gait cycles set at 0.7 m/sec and 5 trials of sit-to-stand and stand-to-sit motions. Muscle activities were sampled using a sEMG (Noraxon, Scottsdale, AZ) with a kinematic motion analysis system [2–4]. The obtained muscle activity signals were rectified, smoothed, and filtered. The amplitude of sEMG during sit-to-stand and stand-to-sit motions was normalized by the average of the maximum amplitude in 10 gait cycles. The difference between the motions was compared using the Wilcoxon signed-rank test. Significance was set at P < 0.05. Since walking is also a general therapeutic exercise, the muscle activities during the sit-to-stand and stand-to-sit motions were compared with those of walking to clarify the difference between the motions.

**Results:** The maximum activities of three muscles during the sit-to-stand and stand-to-sit motions tended to be high compared with walking (Fig. 1). During the sit-to-stand, the %EMG of the VL was higher than that of the stand-to-sit. On the other hand, the %EMG of the GC during sit-to-stand was lower than that of the stand-to-sit. Although the %EMG of the three muscles was similar in the stand-to-sit motion, the VL muscle activity tended to be higher than that of the other muscles during sit-to-stand (Fig. 2). The peak of VL muscle activity appeared in the first half of the sit-to-stand motion.

**Conclusion:** The results indicated the characteristics of the hindlimb muscle activities during the sit-to-stand and stand-to-sit motions of dogs. The first half of the sit-to-stand motion strengthened the VL muscle activity, and the stand-to-sit enhanced the well-coordinated hindlimb extensor muscles compared to walking. Knowing the hind limb extensor muscle activities during sit-to-stand and stand-to-sit motions might help practitioners conduct precise rehabilitation for an individual patient.

**Figure 1 (abstract PO. 28) Figag:**
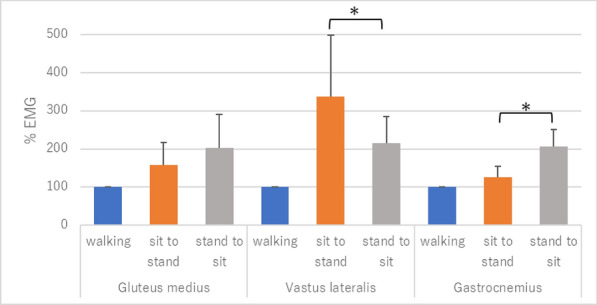
Comparison of activities of the Gluteus medius (GM), vastus lateralis (VL), and gastrocnemius (GC) muscles during walking, sit-to-stand, and stand-to-sit motions. The sit-to-stand and stand-to-sit represented %EMG was normalized by the maximum amplitude of walking. Asterisk indicates a significant difference (P < 0.05) between sit-to-stand and stand-to-sit

**Figure 2 (abstract PO. 28) Figah:**
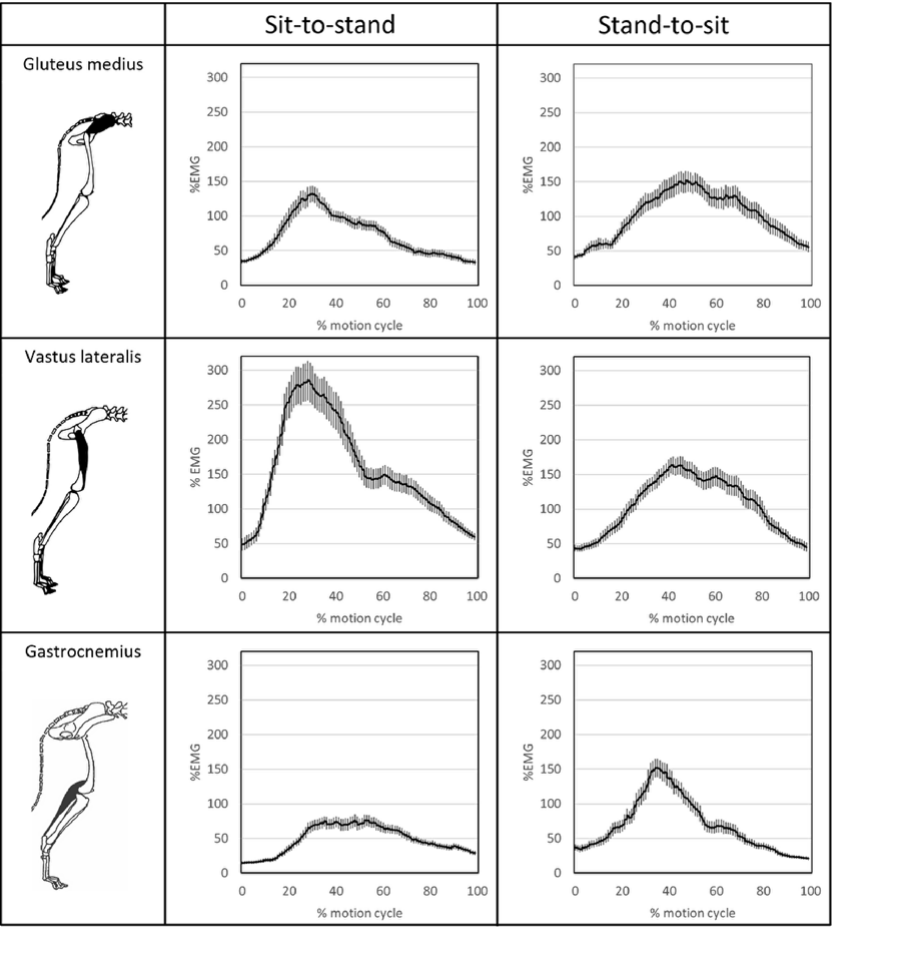
Muscle activities of the Gluteus medius (GM), vastus lateralis (VL), and gastrocnemius (GC) muscles during sit-to-stand and stand-to-sit. The plot shows the mean (solid line) ± SEM (vertical lines). The x-axis represents the % motion cycle. The y-axis represents the %EMG normalized by the maximum amplitude of walking

## References


Millis D, Levine D. Therapeutic Exercises: Joint Motion, Strengthening, Endurance, and Speed Exercises. In: Millis D, Levine D, editors. Canine rehabilitation and physical therapy. 2nd ed. Philadelphia: Elsevier; 2014; 506–25.Breitfuss, Franz M, Peham C, Bockstahler B. Surface electromyography of the vastus lateralis, biceps femoris, and gluteus medius muscle in sound dogs during walking and specific physiotherapeutic exercises. Vet Surg. 2015; 5: 588–95.McLean H, Millis D, Levine D. Surface electromyography of the vastus lateralis, biceps femoris and gluteus medius in dogs during stance, walking, trotting, and selected therapeutic exercises. Front Vet Sci. 2019; 6:211.Yoshikawa K, Tsubakishita S, Sano T, Ino T, Miyasaka T, Kitazawa T. Functional assessment of the gluteus medius, cranial part of the biceps femoris, and vastus lateralis in Beagle dogs based on a novel gait phase classification. J Vet Med Sci. 2021; 83: 116–24.

## PO. 29 Physiotherapy rehabilitation of bilateral chronic equine superficial digital flexor tendon injuries

### **Linda Walker**^1^

#### ^1^Peninsula Physiotherapy, Sidney, BC, Canada, V8M 1G2

##### **Correspondence:** Linda C. Walker (33lindawalker@gmail.com)

*Acta Veterinaria Scandinavica* 2023, **65(Suppl 1)**:PO. 29

**Background:** Superficial digital flexor tendon (SDFT) injuries continue to be one of the most frequent causes of orthopedic lameness and early retirement of performance horses [1].

This case study was designed to highlight the need for change in clinical treatment practices to include a physiotherapy ‘whole horse’ approach in order to more effectively treat SDFT strains. The goal of using a team approach by combining physiotherapy, farrier and veterinary treatments is to safely return the horse to ridden canter work without reinjury.

**Materials and methods:** The patient was a 15-year-old Warmblood gelding ridden in competitive show jumping which has sustained bilateral SDFT strains and a recurrence of strain in the right front SDFT (Fig. 1). He was treated biweekly by a physiotherapist (Diploma Animal Rehab, Equine) over a 5 month period. They included manual therapy/joint mobilizations [3], deep transverse frictions, myofascial release [4], continuous and pulsed ultrasound, core strengthening exercises [5] including resistance bands [6] and weighted hoof boots [7], proprioception/balance exercises[8], in hand and ridden pole exercises [8], a novel application of eccentric, concentric, and speed loading exercises [9–11], in hand and ridden hill work, and moving from firm to deeper footing. Concurrent veterinary treatment included platelet-rich plasma injection with immediate shock wave application followed by 2 subsequent shock wave treatments 2 weeks apart and a conservative walk-trot-canter-ridden protocol. Concurrent farrier work included corrective trimming to increase ease of roll-over and application of ‘suspensory’ aluminum shoes on four feet. Objective measures used were: range of motion using photographs, lameness examinations, diagnostic ultrasound, hoof x-rays, and the functional outcome of returning to walk, trot, and canter, ridden work. Physiotherapy treatment goals were to restore full range of motion in all joints and myofascial tissues, core strength, and conditioning, balance and proprioception, and SDFT elasticity during eccentric, concentric, and speed loading in order to return the horse to ridden flatwork without reinjury (Fig. 2).

**Results:** The patient was successfully returned to ridden canter work and will continue to be able to be ridden for dressage performance and flat work. Due to the extent of the soft tissue injury, the delay in the inclusion of conservative physiotherapy rehabilitation, and having sustained 2 injuries successively to the right SDFT, it is unreasonable to return the patient to jumping activities in the future.

**Conclusion:** Rehabilitation of recurrent SDFT injuries is more effective using combined ‘whole horse’ physiotherapy, veterinary and farrier treatments for a successful return to ridden work. It is recommended that a team approach be applied and further studied, to improve the health of performance horses with SDFT injuries.

**Figure 1 (abstract PO. 29) Figai:**
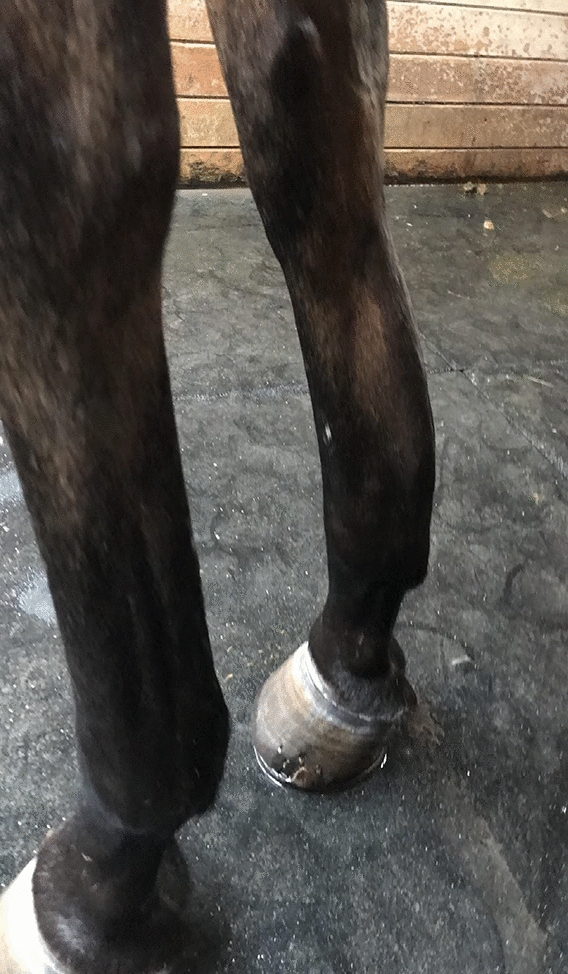
Physiotherapy initial assessment, 2.5 yrs post initial SDFT injury

**Figure 2 (abstract PO. 29) Figaj:**
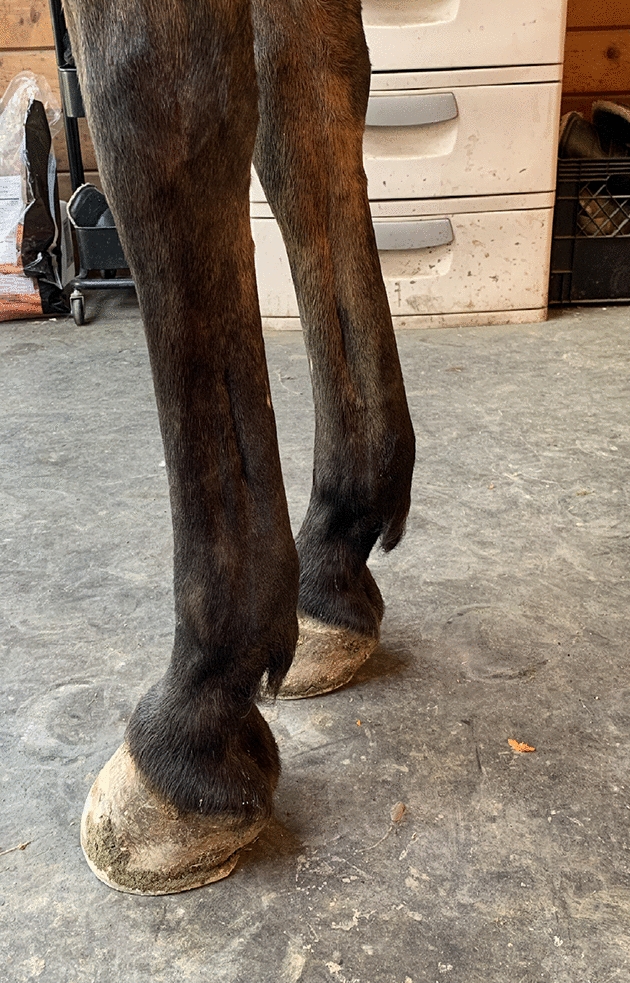
Patient, post 4 months of physiotherapy combined treatment


**Consent to publish**


Informed consent was obtained from the horse’s owner prior to the start of the study, and there was adherence to a high standard (best practice) of physiotherapy, farrier, and veterinary care.


**References**
Clegg PD. Musculoskeletal disease and injury, now and in the future. Part 2: Tendon and ligament injuries. Equine Vet J. 2012; 44:371-5.Goff L. Physiotherapy assessment for the equine athlete. Vet Clin North Am Equine Pract. 2016; 32:31-47.Haussler KK. Equine manual therapies in sport horse practice. Vet Clin North Am Equine Pract. 2018; 34:375-89.Elbrønd VS, Schultz RM. Deep myofascial kinetic lines in horses, Comparative Dissection Studies Derived from Humans. Open J Vet Med. 2021; 11:14.Clayton HM, Kaiser L, Lavagnino M, Stubbs N. Evaluation of intersegmental vertebral motion during performance of dynamic mobilization exercises in cervical lateral bending in horses. Am J Vet Res. 2012; 73:1153-9.Pfau T, Simons V, Rombach N, Stubbs N, Weller R. Effect of a 4-week elastic resistance band training regimen on back kinematics in horses trotting in-hand and on the lunge. Equine Vet J. 2017; 9:829-35.Jensen AM, Ahmed W, Elbrønd VS, Harrison AP. The efficacy of intermittent long-term bell boot application for the correction of muscle asymmetry in equine subjects. J Equine Vet Sci. 2018; 68:73-80.


## PO. 30 Questionnaire assessment of the ability of equestrians in the UK to detect lameness in horses

### Harley Debeuc^1^, **Sarah Roberts**^1^

#### ^1^Animal Health, Behavior and Welfare Department, Harper Adams University, Edgmond, Shropshire, TF10 8NB, UK

##### **Correspondence:** Sarah Roberts (broberts@harper-adams.ac.uk)

Acta Veterinaria Scandinavica 2023, **65(Suppl 1)**:PO. 30

**Introduction:** Lameness in horses is a gait abnormality; the limb is unloaded to relieve pain resulting from musculoskeletal injury or mechanical restrictions [1]. Gold standard lameness quantification is suggested as subjective analysis by veterinarians alongside gait analysis from an inertial sensor system [2,3], which can only be achieved when the owner or handler recognizes a problem. This study aimed to investigate the general UK equestrian population’s ability to detect lameness in horses, and any relationships between the ability to detect lameness and other lifestyle factors.

**Materials and methods:** Video footage was obtained from five lameness workups at an equine hospital; each horse was assessed in walk and trotting along hard surfaces. Lameness diagnoses were made using subjective gait analysis from experienced veterinarians and inertial gait analysis equipment. An online questionnaire was used to obtain responses from across the UK. Previous experience of lameness and years of experience with horses were analyzed one-way ANOVA then a post-hoc Bonferroni test; experience rating with horses using Kruskal Wallis one-way analysis of variance then post-hoc Mann Whitney; the nature of their involvement with horses, were compared to the number of correct lameness identifications scored out of the five videos using U-tests, a chi squared test, two sample T-test.

**Results:** 400 responses were obtained. A significant difference (SD) was observed in participants' involvement with horses and score of correct lameness identifications (χ^2^= 54.2007, d.f.=6, P=0.00001); clinicians scored higher than all other groups (Figure 1). SD existed (χ^2^= 15.08, d.f.=4, P=0.0045) in lame limb identification scores between the group with 0-9 years of experience and other groups (Figure 2). An SD was observed between a group of participants previously seeing lameness in horses and the group of participants who had not (χ^2^= 19.138, d.f.=1, P=0.000012) (Figure 3). An SD was observed between participants' experience rating and their score of correct lameness identifications (χ^2^= 16.56, d.f.=4, P=0.002), with a rating of ‘good’ getting a better score of correct lame limb identifications than a rating of ‘average’ or ‘poor’.

**Figure 1 (abstract PO. 30) Figak:**
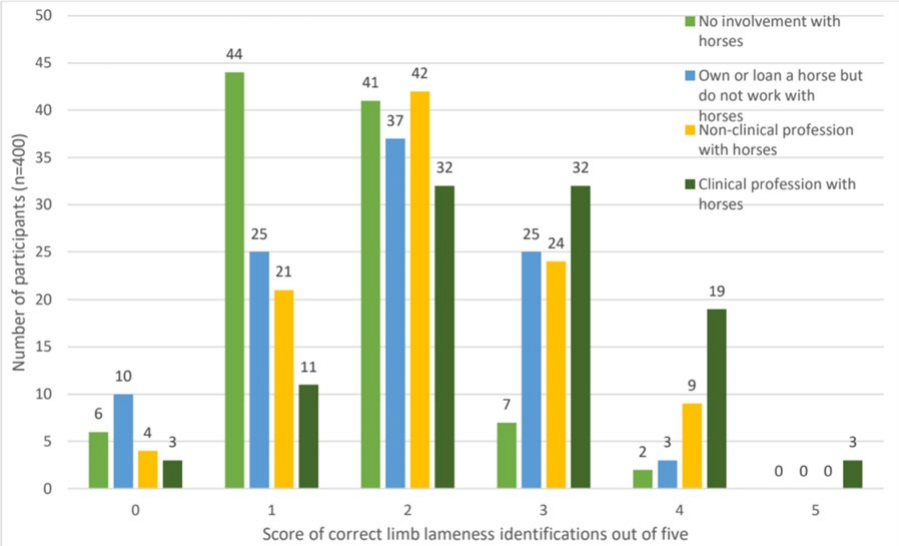
The effect of the participants' main involvement with horses on their number of correct limb lameness identifications in the questionnaire

**Figure 2 (abstract PO. 30) Figal:**
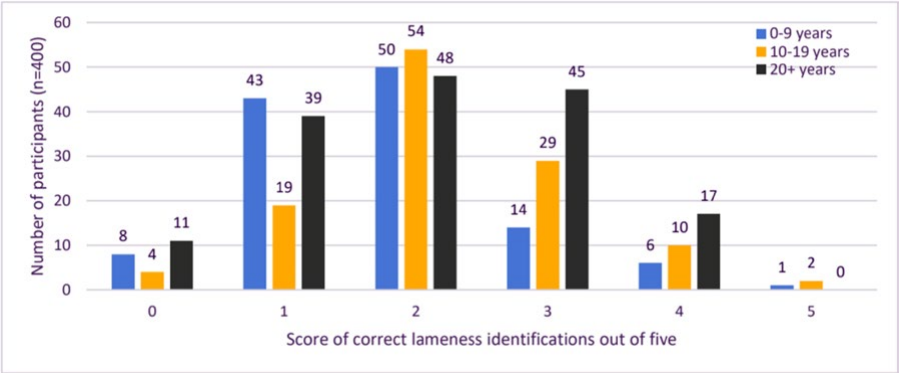
The effect of number of years of experience with horses on the number of correct lameness identifications in the questionnaire

**Figure 3 (abstract PO. 30) Figam:**
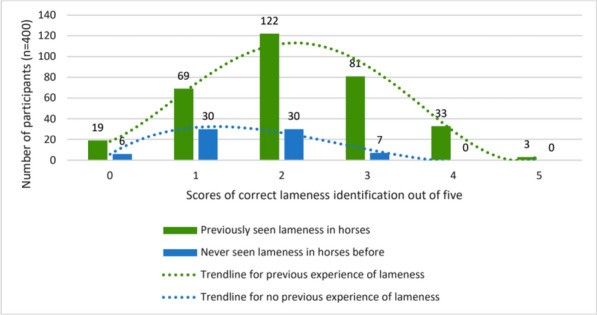
The effect of previously seeing lameness in horses on the number of correctly identified limb lameness' in the questionnaire. Dotted lines indicate trendlines in the data sets

**Conclusion:** An equestrian’s ability to detect lameness has potential implications for the welfare of UK horses; especially for pleasure or lower-level competition as they are often owned by those less experienced. With the lower grade lameness (2/10; 3/10) often being missed, and the findings of this study demonstrating those with the least experience being less able to identify lameness in general, it is of vital importance that supports, and training are provided to such owners to improve animal welfare. Veterinary physiotherapists could play a large role in this education.


**Acknowledgments**


This study was funded in part by the Animal Welfare Foundation (charity number 287118) www.animalwelfarefoundation.org.uk


**References**
Carslake H. 2016. Equine neurological and uscular conditions. In: McGowan C. and Goff L. ed. Animal physiotherapy: assessment, treatment and rehabilitation of animals, 2^nd^ Ed. UK: Wiley BlackwellMcCracken MJ, Kramer J, Keegan KG, Lopes M, Wilson DA, Reed SK, et al. Comparison of an inertial sensor system of lameness quantification with subjective lameness evaluation. Equine Vet J Suppl.2012; 44:652–6Donnell JR, Frisbie DD, King MR, Goodrich LR, Haussler KK. Comparison of subjective lameness evaluation, force platforms and an inertial sensor system to identify mild lameness in an equine osteoarthritis model. Vet J.; 2015; 206:136–42.


## PO. 31 An observational pilot study of foals’ early standing-up behavior

### **Nina Pirinen**^1^, Anna Mykkänen^1^, Catherine McGowan^2^, Heli Hyytiäinen.^1^

#### ^1^Department of Equine and Small Animal Medicine, Faculty of Veterinary Medicine, University of Helsinki, Helsinki, Finland; ^2^Department of Equine Clinical Science, Institute of Veterinary Science, University of Liverpool, Liverpool, UK

##### **Correspondence:** Nina Pirinen (nina.pirinen@helsinki.fi)

*Acta Veterinaria Scandinavica* 2023, **65(Suppl 1)**:PO. 31

**Introduction:** Foals are expected to stand up within two hours after birth: delays could be a sign of illness. The progress of standing-up behavior has not been reported previously, despite its possible clinical significance, thus this observational pilot study was performed.

**Materials and methods:** Two healthy colts (one Finnhorse (F)) and one Finnish Warm Blood (FWB)) were video recorded from birth to 12 h of age. Attempts to stand up and standing up were recorded as total numbers of behaviors using the CowLog- behavioral coding software [1].

**Results:** First attempts to stand occurred 3 (F) and 4 min (FWB) after birth. Foals tried 73 (F) and 50 (FWB) times before first actual standing up. For the second try, they needed 4 (F) and 3 (FWB) attempts. The attempts per standing up varied between 0 and 35 (F), and 0 and 25 (FWB), with a median of 2 and 4, respectively. During the first 12 h, foals attempted to stand altogether 157 (F) and 118 (FWB) times and they stood up 11 (F) and 10 (FWB) times.

**Conclusion:** Foals tried to stand shortly after birth. Many attempts were needed before foals were able to stand up the first time, but fewer attempts were needed for the following stands. There was a lot of variance in attempts needed between each stand, and between foals. Thus, further studies with more foals are needed with a longer monitoring period.


**Reference**
Pastell, M. CowLog – Cross-platform application for coding behaviors from video. J Open Res Softw. 2016;4:15.


