# Correction to: Proceedings of the 11th International Association of Veterinary Rehabilitation and Physical Therapy Symposium

**DOI:** 10.1186/s13028-024-00729-x

**Published:** 2024-03-14

**Authors:** Anja Pedersen, Anna Bergh, Linn Dadell, Anja Babra

**Affiliations:** Swedish University of Agricultural Sciences SLU, Uppsala, Sweden

**Correction to: Acta Veterinaria Scandinavica (2023) 65:55**

10.1186/s13028-023-00706-w

Following publication of the original article [1], we have been notified that the title of the meeting abstracts was not published correctly. It is shown below:

Proceedings of the 11th International Association of Veterinary Rehabilitation and Physical Therapy, and the Summit of the American Association of Rehabilitation Veterinarians and the American College of Veterinary Sports Medicine and Rehabilitation.

It should be as per below:

Proceedings of the 11th International Association of Veterinary Rehabilitation and Physical Therapy Symposium.

The original article was updated.
